# Synthesis of 1,2,3-triazoles using Grignard reactions through the protection of azides

**DOI:** 10.3389/fchem.2023.1237878

**Published:** 2023-07-31

**Authors:** Rina Namioka, Minori Suzuki, Suguru Yoshida

**Affiliations:** ^1^ Department of Biological Science and Technology, Faculty of Advanced Engineering, Tokyo University of Science, Tokyo, Japan; ^2^ Laboratory of Chemical Bioscience, Institute of Biomaterials and Bioengineering, Tokyo Medical and Dental University (TMDU), Tokyo, Japan

**Keywords:** azides, triazoles, protection, click chemistry, iodine–magnesium exchange, turbo Grignard reagent, phosphazide, phosphines

## Abstract

An efficient method to prepare organomagnesium intermediates having a protected azido group is reported. Protection of azido groups with di-(*tert*-butyl)(4-(dimethylamino)phenylphosphine (amphos) and following iodine–magnesium exchange realized the preparation of organomagnesium intermediates, which served in the synthesis of diverse azides by transformation with various electrophiles followed by deprotection with elemental sulfur. Furthermore, click reactions of azides with alkynes enabled synthesizing a wide variety of 1,2,3-triazoles.

## 1 Introduction

**FIGURE 1:**
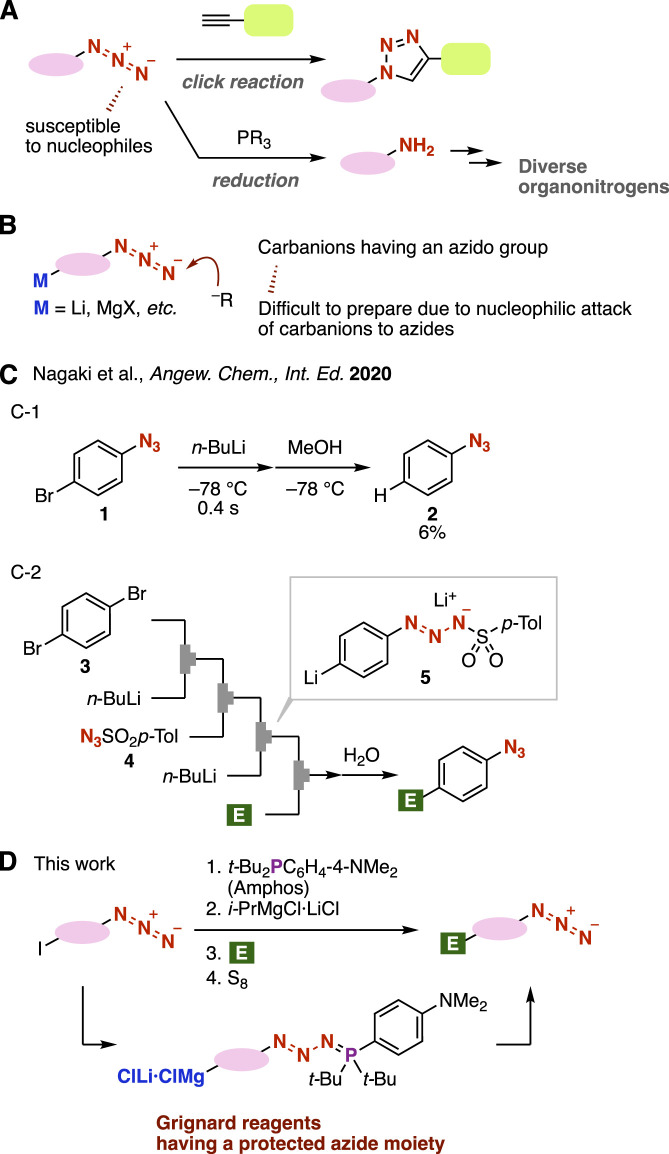
**(A)** Transformations of azides. **(B)** Carbanions having an azido group. **(C)** Nagaki’s work. **(D)** Overview of this work.

**FIGURE 2:**
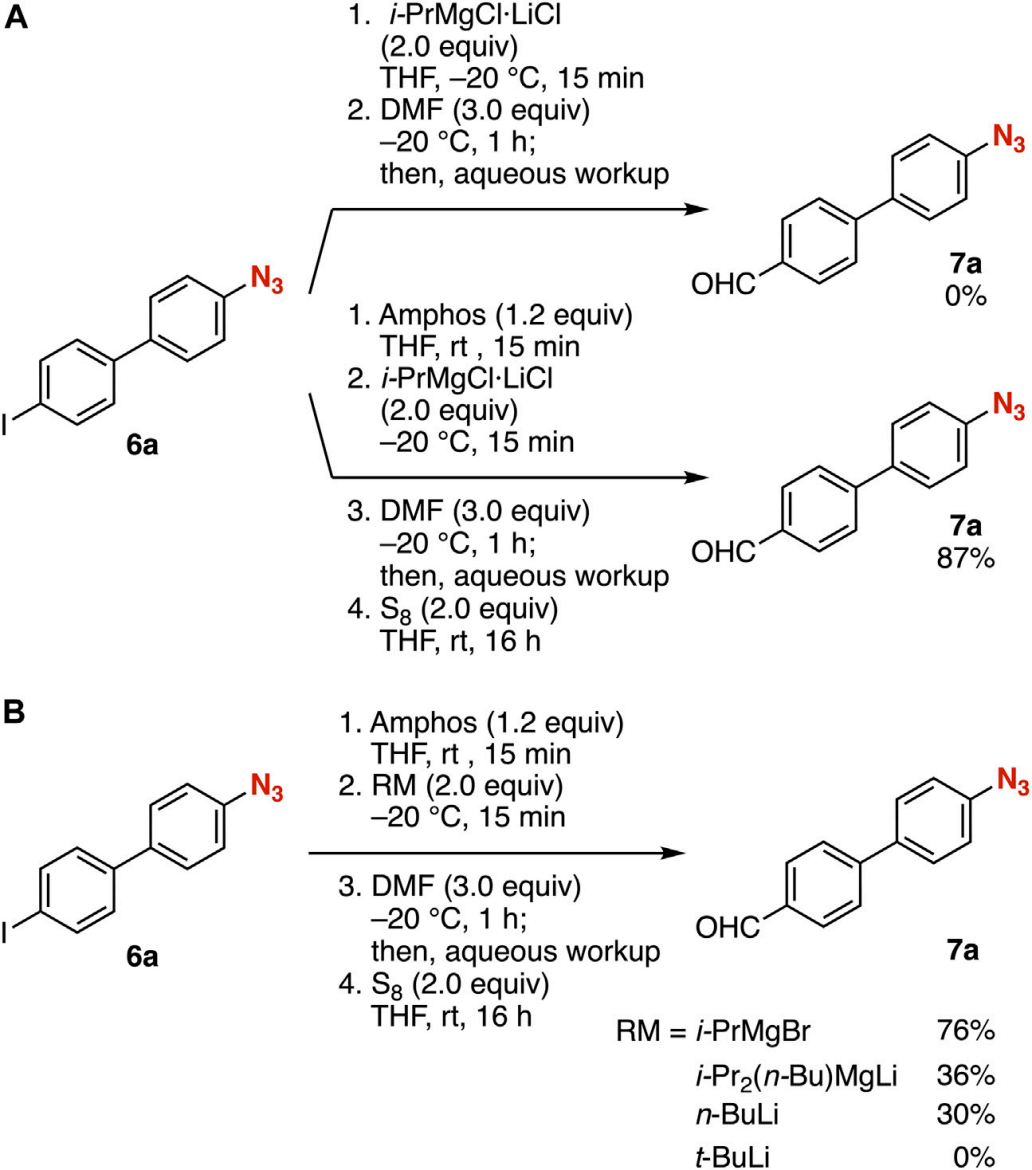
**(A)** Synthesis of **7a** from **6a** with or without the protection of the azido group. **(B)** Screening reagents for the iodine–metal exchange.

Azides are a significant class of compounds in a broad range of research fields, including synthetic organic chemistry, pharmaceutical sciences, and materials chemistry (Figure 1A) (Bräse and Banert, 2010; Banert, 2016; Yoshida, 2020). Triazole formations by copper-catalyzed azide–alkyne cycloaddition (CuAAC) (Rostovtsev et al., 2002; Tornøe et al., 2002; Meldal and Tornøe, 2008) or strain-promoted azide–alkyne cycloaddition (SPAAC) (Agard et al., 2004; Ning et al., 2008; Dommerholt et al., 2010) have served as click reactions. Azides are also frequently used in organonitrogen syntheses through the Staudinger reduction which takes place smoothly by the treatment of phosphines at ambient temperature (Staudinger and Meyer, 1919; Saxon and Bertozzi, 2000). Despite the importance of azides in synthetic organic chemistry, it is not always easy to synthesize azides owing to the electrophilic nature of azido groups which are susceptible to various nucleophiles, such as carbanions (Tanimoto and Kakiuchi, 2013). In particular, the preparation of carbanions having azido groups is, thus, a challenging issue (Figure 1B). For example, Nagaki and coworkers reported that treatment of 4-bromophenyl azide with *n*-butyllithium at −78°C under microflow conditions followed by protonation afforded phenyl azides in low yield (Figure 1C–1) (Ichinari et al., 2020), notably showing that the preparation of 4-azidophenyllithium is a challenging transformation, even under microflow conditions. An alternative preparation method for the 4-azidophenyllithium equivalent was successfully developed from 1,4-dibromobenzene (**3**) under microflow conditions through triazene formation with sulfonyl azide **4** and the subsequent bromine–lithium exchange, leading to aryllithium **5**, as a carbanion, having a masked azide moiety (Figure 1C–2) (Ichinari et al., 2020). Although this elegant method allowed us to synthesize a limited variety of 4-substituted phenyl azides, a new approach to prepare carbanions bearing masked azide moieties leading to a wide array of azides is sought after.

In this study, we conceived an idea of preparing carbanions having “protected” azido groups through the treatment of azides with di(tert-butyl)(4-(dimethylamino)phenylphosphine (amphos) (Figure 1D). Previously, we found that amphos smoothly reacts with azides to furnish phosphazides without denitrogenation, and phosphazides can be transformed into azides through deprotection with elemental sulfur (Meguro et al., 2018). Azide protection realized various transformations, such as selective click reactions of diazides and Grignard reactions using carbonyl compounds having azide moieties due to the good stability of phosphazides as protected azides (Aimi et al., 2021). Herein, we describe an efficient method to prepare organomagnesium intermediates by iodine–magnesium exchange with a turbo Grignard reagent after the phosphazide formation of iodine-substituted azides, enabling facile synthesis of diverse 1,2,3-triazoles by Grignard reactions and following CuAAC reactions.

## 2 Results and discussion

First, we attempted the iodine–magnesium exchange of 4-(4-iodophenyl)phenyl azide (**6a**) with an isopropylmagnesium chloride lithium chloride complex (Krasovskiy and Knochel, 2004; Bao et al., 2015) in THF at −20°C followed by the addition of *N*,*N*-dimethylformamide (DMF) (Figure 2A, route 1). As a result, the desired aldehyde **7a** was not obtained due to the decomposition of the azido group. In contrast, we succeeded in the synthesis of aldehyde **7a** from iodide **6a** in high yield via phosphazide formation (Figure 2A, route 2). Treatment of azide **6a** with amphos at room temperature followed by iodine–magnesium exchange with the isopropylmagnesium chloride lithium complex in THF at −20°C and subsequent addition of DMF resulted in efficient formylation. Following deprotection of the phosphazide moiety with elemental sulfur provided azide **7a** in good yield without damaging the azido group. The iodine–magnesium exchange with isopropylmagnesium bromide instead of the turbo Grignard reagent also proceeded efficiently (Figure 2B). Aldehyde **7a** was prepared in moderate yield when using *i*-Pr_2_(*n*-Bu)MgLi (Inoue et al., 2001) or *n*-butyllithium for the iodine–metal exchange. Metalation using *tert*-butyllithium resulted in a complex mixture of products.

**FIGURE 3:**
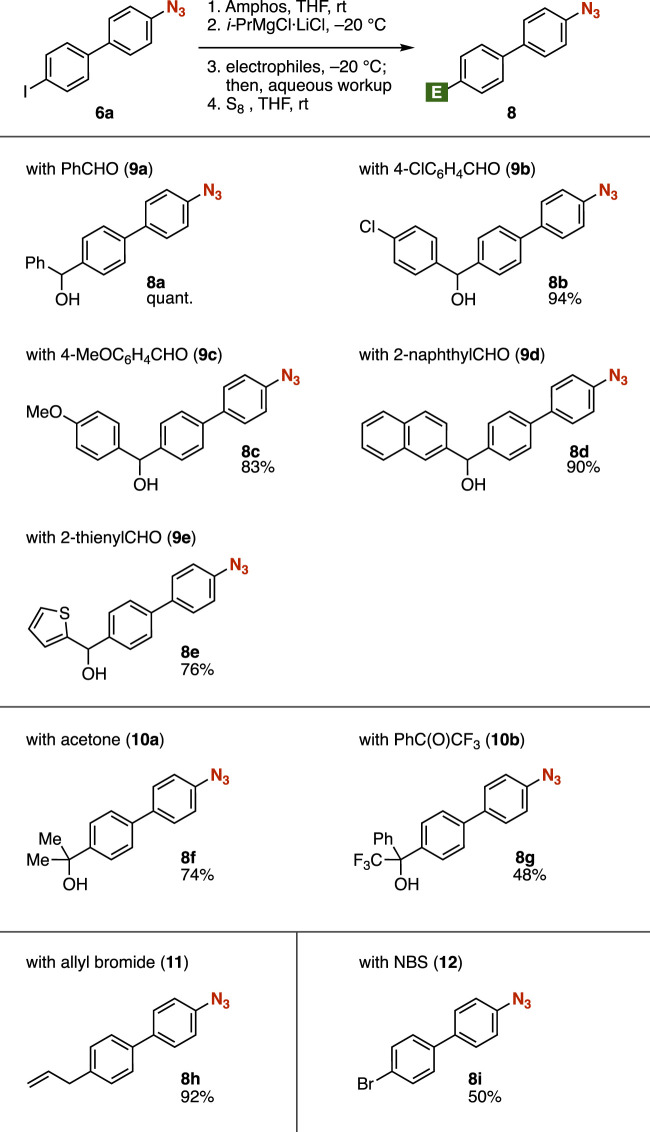
Synthesis of azides **8** from azide **6a** and various electrophiles. NBS, *N*-bromosuccinimide.

A wide range of azides **8** were successfully synthesized by the addition of electrophiles to the organomagnesium intermediate prepared *in situ* from azide **6a** (Figure 3). Various aldehydes **9** efficiently reacted with the organomagnesium intermediate, enabling us to synthesize the corresponding alcohols **8a**–**8e** in good yields, leaving azide, benzyl alcohol, chloro, methoxy, and thienyl moieties intact. Tertiary alcohol **8f** or **8g** was prepared from azide **6a** using acetone (**10a**) or α,α,α-trifluoroacetophenone (**10b**), respectively, as an electrophile. Allylation of the Grignard reagent prepared from **6a** took place to afford the azide **8h** after deprotection with elemental sulfur. Bromide **8i** was synthesized by bromination of the carbanion with *N*-bromosuccinimide (NBS), followed by treatment with S_8_.

**FIGURE 4:**
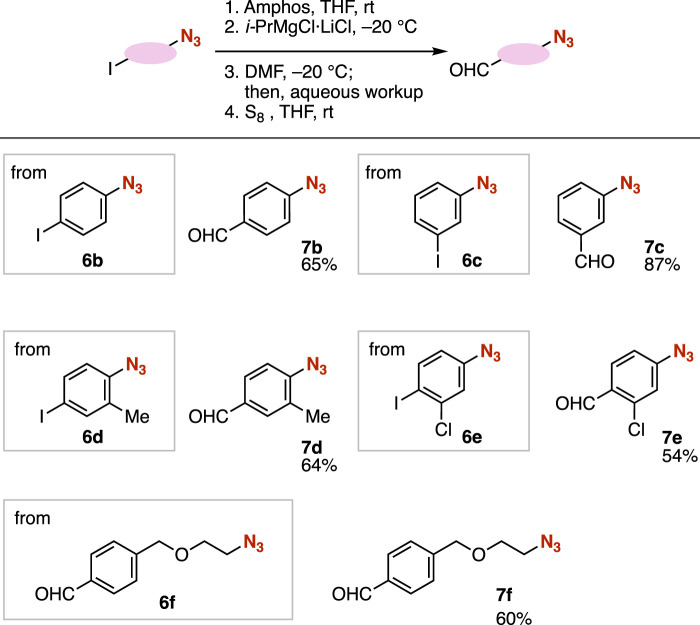
Synthesis of azides **7** from various azides **6**.

We succeeded in the synthesis of aldehydes **7b**–**7f** from a range of azides **6** through phosphazide formation, iodine–magnesium exchange, formylation with DMF, and deprotection with S_8_ (Figure 4). For example, 4-formyl- or 3-formylphenyl azide **7b** or **7c** were prepared from 4-iodo- or 3-iodophenyl azide (**6b** or **6c**), respectively. We accomplished the synthesis of trisubstituted benzene **7d** from 4-azido-3-methylphenyl iodide (**6d**) through the phosphazide formation of the *ortho* methyl-substituted phenyl azide moiety. When using 4-azido-2-chlorophenyl iodide (**6e**), protection of the azido group, iodine–magnesium exchange, formylation, and deprotection proceeded smoothly for furnishing aldehyde **7e** without damaging aldehyde, azide, and chloro moieties. Moreover, we achieved the preparation of the carbanion intermediate having an alkyl azide moiety from azide **6f** through phosphazide formation and subsequent iodine–magnesium exchange, which successfully served in formylation with DMF.

**FIGURE 5:**
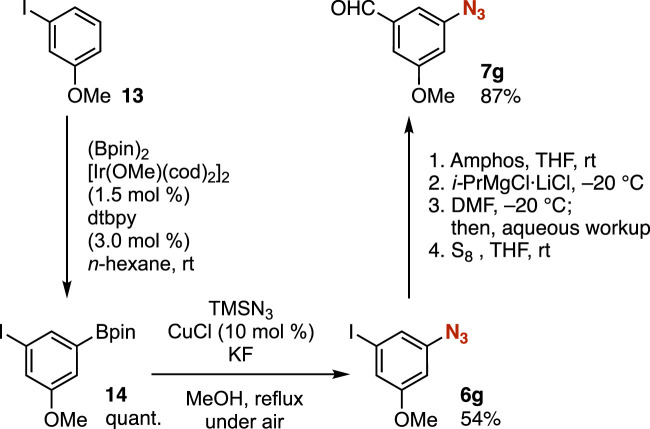
Synthesis of azide **7g**.

Azides bearing the iodo group can be synthesized by formal C–H azidation (Yoshida et al., 2014; Nishiyama et al., 2019) through Ir-catalyzed C–H borylation (Cho et al., 2002; Ishiyama et al., 2002; Mkhalid et al., 2010) and subsequent Cu-catalyzed azidation (Li et al., 2010). Thus, transformations of aryl iodides via the protection of the azido group allowed us to prepare a wide range of highly functionalized aryl azides from simple aryl iodides. For example, C–H borylation of *m*-iodoanisole catalyzed by iridium proceeded smoothly without damaging the iodo group (Figure 5). Subsequent azidation of the resulting arylboron **14**, catalyzed by copper, took place efficiently. Then, we succeeded in the transformation of aryl iodide **6g** via phosphazide formation and the iodine–magnesium exchange to provide benzaldehyde **7g,** leaving the methoxy, formyl, and azido groups untouched.

**FIGURE 6:**
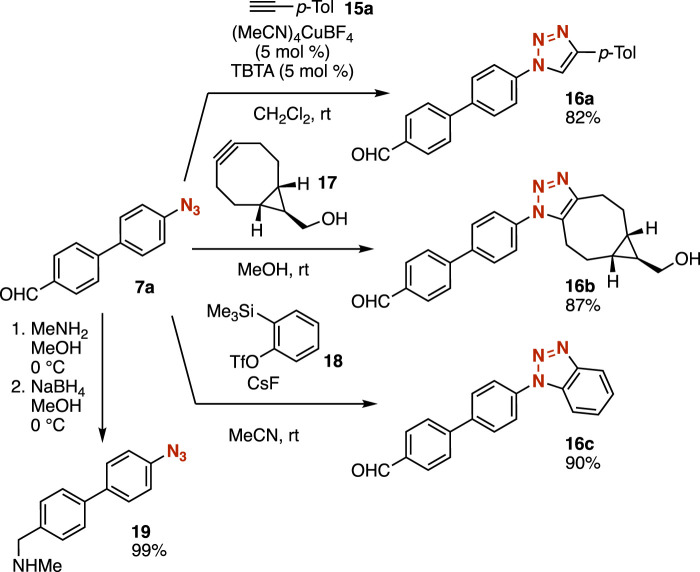
Transformations of azide **7a**.

A wide variety of 1,2,3-triazoles were easily synthesized from azide **7a** without damaging the formyl group (Figure 6). Indeed, we accomplished the synthesis of triazole **16a** in high yield by the CuAAC reaction of azide **7a** with terminal alkyne **15a** in the presence of a catalytic amount of (MeCN)_4_CuBF_4_ and tris[(1-benzyl-1*H*-1,2,3-triazol-4-yl)methyl]amine (TBTA) (Chan et al., 2004). Triazole formation of azide **7a** with cycloalkyne **17** proceeded smoothly to afford triazole **16b** in good yield without copper catalysis (Dommerholt et al., 2010). We succeeded in the cycloaddition of azide **7a** with benzyne generated from *o*-silylaryl triflate **18** to provide benzotriazole **16c** having an aldehyde moiety (Shi et al., 2008). Reductive amination of aldehyde **7a** also took place avoiding the reduction of the azido group. Thus, further transformations enabled us to diversify azides after reactions of azide-substituted carbanion equivalents.

**FIGURE 7:**
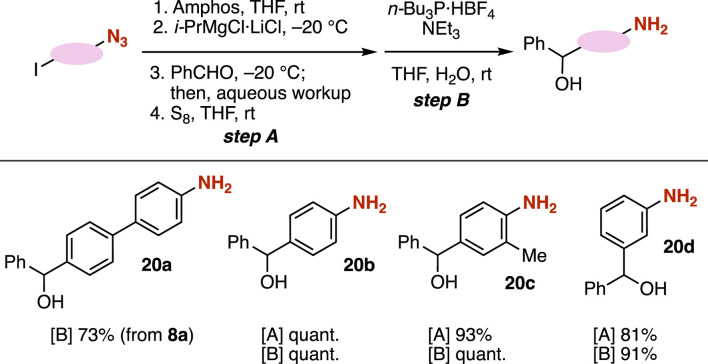
Transformations to anilines **20**.

Amino alcohols **20a**–**20d** were efficiently synthesized from azides by transformations of carbanions through the protection of azido groups followed by the Staudinger reduction (Figure 7). We achieved the synthesis of amino alcohol **20a** from azide **8a** with tri-(*n*-butyl)phosphonium tetrafluoroborate in the presence of triethylamine (Meguro et al., 2017). The Grignard reaction of azide-substituted carbanion equivalents with aldehyde **9a** and the following Staudinger reduction realized the preparation of a number of amino alcohols **20b**–**20d** in good yields. Considering the pivotal role of amines and alcohols in the preparation of azaheterocycles, the synthesis of amino alcohols from iodine-substituted azides is poised to make significant contributions to the field of synthetic organic chemistry.

**FIGURE 8:**
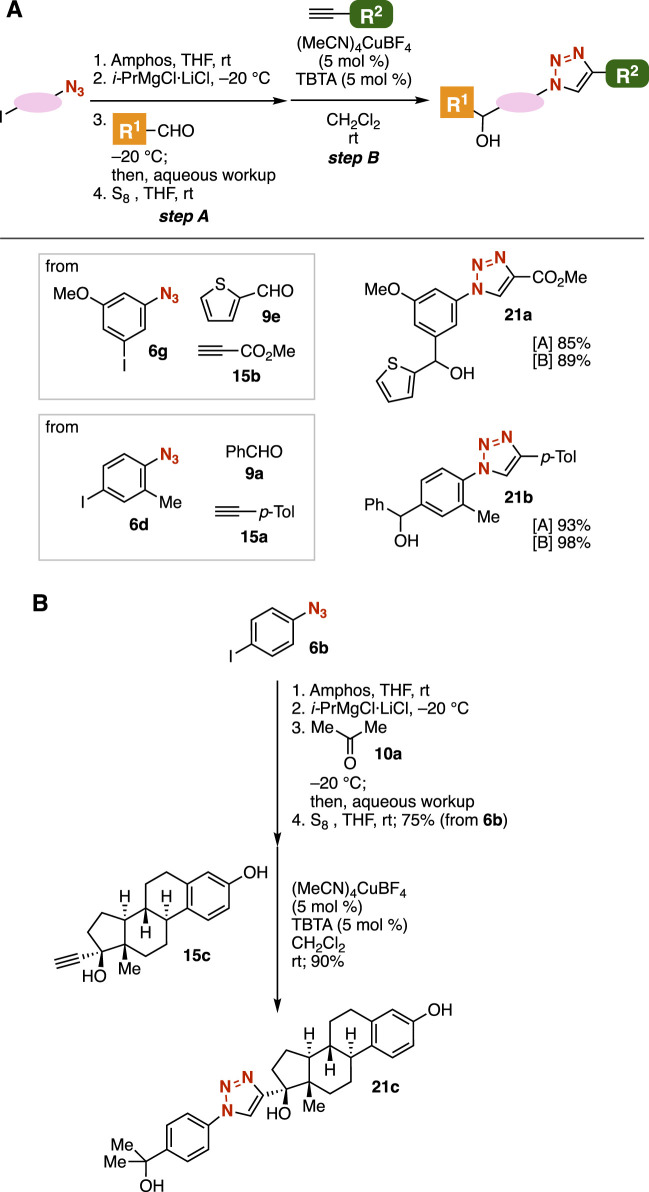
**(A)** Synthesis of triazoles **21a** and **21b**. **(B)** Synthesis of triazole **21c**.

Grignard reactions of azide-substituted carbanion equivalents and the subsequent CuAAC reaction enabled us to synthesize a broad variety of 1,2,3-triazoles from diverse azides, aldehydes, and terminal alkynes (Figure 8). After treatment of 3-iodo-5-methoxyphenyl azide (**6g**) with amphos followed by the iodine–magnesium exchange, the Grignard reaction with 2-thienyl aldehyde (**9e**) and deprotection with elemental sulfur resulted in the efficient synthesis of the corresponding alcohol in good yield (Figure 8A). Then, we succeeded in the preparation of triazole **21a** by the CuAAC reaction with alkyne **15b** bearing an ester moiety. This approach is clearly advantageous over a synthetic route without azide protection, as esters can readily react with carbanions like Grignard reagents. Consequently, the synthesis of triazole **21a** from azide **6g**, aldehyde **9e**, and alkyne **15b** was achieved in short steps. Furthermore, triazole **21b** was efficiently prepared from azide **6d**, aldehyde **9a**, and alkyne **15a**. We achieved the synthesis of triazole **21c** bearing an estradiol scaffold from 4-iodophenyl azide (**6b**), acetone (**10a**), and ethinyl estradiol (**15c**) by a simple protocol through phosphazide formation.

## 3 Materials and method

For general experimental and instrumental methods, synthetic procedures, and full compound characterization, see the Supplementary Materials.

### 3.1 Synthesis of aldehyde **7a** from aryl iodide **6a**

To a solution of 4-azido-4'-iodo-1,1'-biphenyl (**6a**) (96.8 mg, 0.301 mmol) dissolved in THF (4.0 mL) was added di(*tert*-butyl)(4-(dimethylamino)phenyl)phosphine (amphos) (95.9 mg, 0.361 mmol, and 1.2 equiv) at room temperature. After stirring for 15 min at the same temperature, we slowly added *i*PrMgCl・LiCl (1.3 M, THF solution, 0.50 mL, 0.650 mmol, and 2.2 equiv) to it at −20°C. After stirring for 30 min at the same temperature, we also slowly added *N*,*N*-dimethylformamide (70.0 µL, 0.904 mmol, and 3.0 equiv) to the solution. After stirring for 1 h at −20°C, we slowly added water (5 mL) to it. The mixture was extracted with EtOAc (10 mL × 3). The combined organic extract was washed with brine (10 mL) and dried with Na_2_SO_4_. After filtration, the filtrate was concentrated under reduced pressure. We added S_8_ (19.7 mg, 0.614 mmol, and 2.0 equiv) to the residue dissolved in THF (4.0 mL) at room temperature. After stirring for 16 h at the same temperature, the mixture was concentrated under reduced pressure. The residue was purified by preparative TLC (*n*-hexane/EtOAc = 1/1) to give 4-(4-azidophenyl)benzaldehyde (**7a**) (55.6 mg, 0.249 mmol, and 83%) as a pale yellow solid.

### 3.2 4-Azido-4′-iodo-1,1′-biphenyl (**6a**)

Pale yellow solid; Mp 122–124°C; TLC *R*_f_ 0.65 (*n*-hexane/EtOAc = 10/1); ^1^H NMR (CDCl_3_, 400 MHz): δ 7.07–7.13 (AA’BB’, 2H), 7.25–7.33 (AA’BB’, 2H), 7.51–7.57 (AA’BB’, 2H), and 7.74–7.79 (AA’BB’, 2H); ^13^C{^1^H} NMR (CDCl_3_, 101 MHz): δ 93.1, 119.5, 128.2, 128.6 (two signals overlapped), 136.7, 137.9, and 139.6; IR (Nujol, cm^–1^): 810, 1,296, 1,306, 1,377, 1,388, 1,463, 1,478, 2,106, 2,139, 2,855, 2,924, and 2,953; and HRMS (FAB) *m/z*: [M]^·+^ calcd for C_12_H_8_IN_3_^·+^ 320.9763; found 320.9779.

## 4 Conclusion

In conclusion, we succeeded in the preparation of organomagnesium intermediates having protected azido groups. Various azides were successfully synthesized by the Grignard reaction of carbanions having phosphazide moieties with various electrophiles followed by deprotection with elemental sulfur. Since a broad range of organonitrogens, such as amines and triazoles, are easily prepared from azides, reactions involving carbanion equivalents with azide moieties, followed by subsequent transformations, are poised to significantly contribute to organonitrogen synthesis. Our laboratory is currently engaged in further studies, including the preparation and transformations of carbanions with phosphazide moieties.

## Data Availability

The original contributions presented in the study are included in the article/Supplementary Materials; further inquiries can be directed to the corresponding author.
