# Supplementary material for: Synthesis of 1,2,3-triazoles using Grignard reactions through the protection of azides
Source: Front Chem. 2023 Jul 31;11:1237878. doi: 10.3389/fchem.2023.1237878 (PMC10424848; doi:10.3389/fchem.2023.1237878)

Supporting Information

Synthesis of 1,2,3-Triazoles by Grignard Reactions through the Protection of Azides

Rina Namioka,[a] Minori Suzuki,[a,b] Suguru Yoshida[a]*

[a]*Department of Biological Science and Technology, Faculty of Advanced Engineering, Tokyo University of Science, 6-3-1 Niijuku, Katsushika-ku Tokyo 125-8585*
[b]*Laboratory of Chemical Bioscience, Institute of Biomaterials and Bioengineering, Tokyo Medical and Dental University (TMDU), 2-3-10 Kanda-Surugadai, Chiyoda-ku, Tokyo 101-0062, Japan*

Contents

General Information S1
Experimental Procedures S2
Characterization Data of New Compounds S4
References for Supporting Information S10
^{1}H and ^{13}C NMR Spectra of Compounds S11

General Information

All reactions were performed with dry glassware under atmosphere of argon, unless otherwise noted. Analytical thin-layer chromatography (TLC) was performed on precoated (0.25 mm) silica-gel plates (Merck Chemicals, Silica Gel 60 F254, Cat. No. 1.05715). Column chromatography was conducted using silica-gel (Kanto Chemical Co., Inc., Silica Gel 60N, spherical neutral, particle size 40–50 µm, Cat. No. 37562-85 or particle size 63–210 µm, Cat. No. 37565-85). Preparative TLC (PTLC) was performed on silica gel (Wako Pure Chemical Industries Ltd., Wakogel B-5F, Cat. No. 230-00043). Melting points (Mp) were measured on an OptiMelt MPA100 (Stanford Research Systems), and are uncorrected. ^{1}H NMR spectra were obtained with a Bruker AVANCE 400 spectrometer at 400 MHz. ^{13}C NMR spectra were obtained with a Bruker AVANCE 400 spectrometer at 101 MHz. ^{19}F NMR spectra were obtained with a Bruker AVANCE 400 spectrometer at 376 MHz. All NMR measurements were carried out at 25 °C. $CDCl_3$ (Kanto Chemical Co. Inc., Cat. No. 07663-23) was used as a solvent for obtaining NMR spectra. Chemical shifts (δ) are given in parts per million (ppm) downfield from the solvent peak (δ 7.26 for ^{1}H NMR in $CDCl_3$, δ 77.0 for ^{13}C NMR in $CDCl_3$) as an internal reference with coupling constants (*J*) in hertz (Hz). The abbreviations s, d, t, q, and m signify singlet, doublet, triplet, quartet, and multiplet, respectively. High-resolution mass spectra (HRMS) were measured on a JEOL JMS-T100CS "AccuTOF CS" mass spectrometer under positive or negative electrospray ionization (ESI) conditions or JMS-700 (JEOL, Tokyo, Japan) mass spectrometer under electron impact ionization (EI) conditions.

Unless otherwise noted, materials obtained from commercial suppliers were used without further purification. 1-Azido-4-bromobenzene (**1**),[S1] 1-azido-4-iodobenzene (**6b**),[S2] and 1-azido-3-iodobenzene (**6c**)[S2] were prepared according to the reported methods. According to the procedure for the preparation of ethyl 1-azido-3-iodobenzoate, 1-azido-3-iodo-5-methoxybenzene (**6g**)[S3] was prepared from *m*-iodoanisole.

Experimental Procedures

A typical procedure for Grignard reactions through the protection of azides

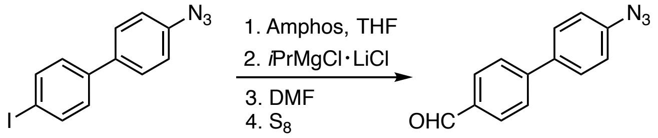

To a solution of 4-azido-4'-iodo-1,1'-biphenyl (**6a**) (96.8 mg, 0.301 mmol) dissolved in THF (4.0 mL) was added di(*tert*-butyl)(4-(dimethylamino)phenyl)phosphine (Amphos) (95.9 mg, 0.361 mmol, 1.2 equiv) at room temperature. After stirring for 15 min at the same temperature, to this was slowly added *i*PrMgCl▪LiCl (1.3 M, THF solution, 0.50 mL, 0.650 mmol, 2.2 equiv) at –20 °C. After stirring for 30 min at the same temperature, to this was slowly added *N*,*N*-dimethylformamide (70.0 µL, 0.904 mmol, 3.0 equiv). After stirring for 1 h at –20 °C, to this was slowly added water (5 mL). The mixture was extracted with EtOAc (10 mL × 3). The combined organic extract was washed with brine (10 mL) and dried with Na_2SO_4. After filtration, the filtrate was concentrated under reduced pressure. To the residue dissolved in THF (4.0 mL) was added S_8 (19.7 mg, 0.614 mmol, 2.0 equiv) at room temperature. After stirring for 16 h at the same temperature, the mixture was concentrated under reduced pressure. The residue was purified by preparative TLC (*n*-hexane/EtOAc = 1/1) to give 4-(4-azidophenyl)benzaldehyde (**7a**) (55.6 mg, 0.249 mmol, 83%) as a pale yellow solid.

A typical procedure for the copper-catalyzed triazole formation

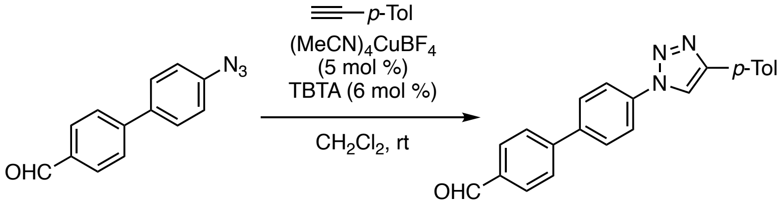

To a solution of 4-(4-azidophenyl)benzaldehyde (**7a**) (11.0 mg, 49.3 µmol) in CH_2Cl_2 (0.50 mL) were added *p*-ethynyltoluene (**15a**) (7.6 µL, 60 µmol), $(MeCN)_4CuBF_4$ (0.8 mg, 3 µmol), and TBTA (1.5 mg, 2.8 µmol) at room temperature. After stirring for 18 h at the same temperature, the mixture was concentrated under reduced pressure. The residue was purified by preparative TLC (*n*-hexane/EtOAc = 2/1) to give 4'-(4-(*p*-tolyl)-1*H*-1,2,3-triazol-1-yl)-[1,1'-biphenyl]-4-carbaldehyde (**16a**) (13.7 mg, 40.4 µmol, 82%) as a colorless solid.

Synthesis of 4'-((5aS,6S*,6aR*)-6-(hydroxymethyl)-5,5a,6,6a,7,8-hexahydrocyclopropa[5,6]cycloocta[1,2-d][1,2,3]triazol-1(4H)-yl)-[1,1'-biphenyl]-4-carbaldehyde (**16b**)*

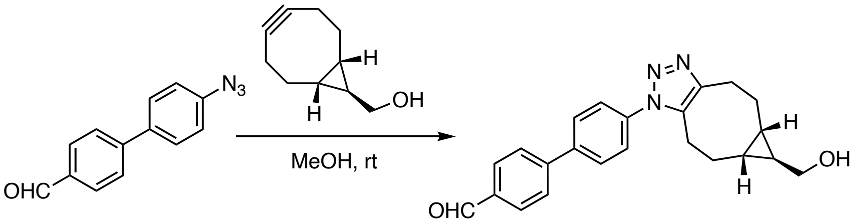

To a solution of 4-(4-azidophenyl)benzaldehyde (**7a**) (10.9 mg, 48.9 µmol) in MeOH (0.50 mL) was added (1α,8α,9α)-bicyclo[6.1.0]non-4-yn-9-ylmethanol (8.7 mg, 58 µmol) at room temperature. After stirring for 24 h at the same temperature, the mixture was concentrated under reduced pressure. The residue was purified by preparative TLC (*n*-hexane/EtOAc = 1/1) to give 4'-((5*aS**,6*S**,6*aR**)-6-(hydroxymethyl)-5,5a,6,6a,7,8-hexahydrocyclopropa[5,6]cycloocta[1,2-*d*][1,2,3]triazol-1(4*H*)-yl)-[1,1'-biphenyl]-4-carbaldehyde (**16b**) (15.9 mg, 42.6 µmol, 87%) as a pale yellow solid.

*Synthesis of 4'-(1H-benzo[d][1,2,3]triazol-1-yl)-[1,1'-biphenyl]-4-carbaldehyde (**16c**)*

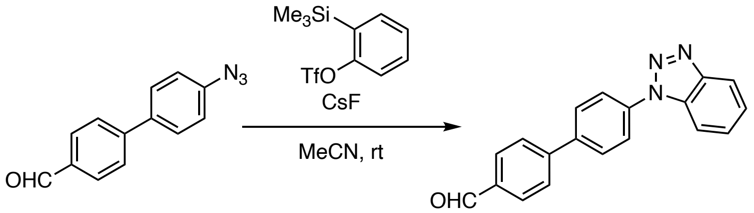

To a solution of 4-(4-azidophenyl)benzaldehyde (**7a**) (22.3 mg, 0.100 mmol) in acetonitrile (2.0 mL) were added *o*-(trimethylsilyl)phenyl triflate (**18**) (44.6 mg, 0.149 mmol) and cesium fluoride (43.5 mg, 0.286 mmol) at room temperature. After stirring for 24 h at the same temperature, to this was added water (5 mL). The mixture

was extracted with EtOAc (10 mL × 3). The combined organic extract was washed with brine (10 mL) and dried with Na_2SO_4. After filtration, the filtrate was concentrated under reduced pressure. The residue was purified by preparative TLC (*n*-hexane/EtOAc = 1/1) to give 4'-(1*H*-benzo[*d*][1,2,3]triazol-1-yl)-[1,1'-biphenyl]-4-carbaldehyde (**16c**) (27.0 mg, 90.2 μmol, 90%) as a pale yellow solid.

*Synthesis of 1-(4'-azido-[1,1'-biphenyl]-4-yl)-N-methylmethanamine (**19**)*

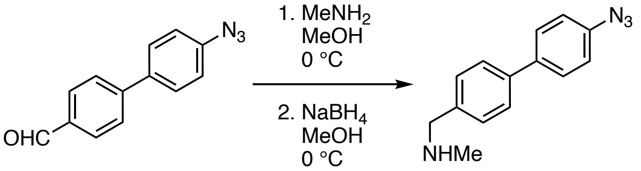

To a solution of 4-(4-azidophenyl)benzaldehyde (**7a**) (22.0 mg, 98.7 μmol) in methanol (0.90 mL) was added methylamine (90 μL, 40% aqueous solution, 99 μmol) at room temperature. After stirring for 30 min at the same temperature, to the mixture was added sodium borohydride (1.9 mg, 50 μmol) at 0 °C. After stirring for 1 h at the same temperature, to this was added water (5 mL). The mixture was extracted with EtOAc (10 mL × 3). The combined organic extract was washed with brine (10 mL) and dried with Na_2SO_4. After filtration, the filtrate was concentrated under reduced pressure. The residue was purified by preparative TLC (EtOAc only) to give 1-(4'-azido-[1,1'-biphenyl]-4-yl)-*N*-methylmethanamine (**19**) (23.2 mg, 97.4 μmol, 99%) as a pale yellow solid.

A typical procedure for the reduction of azides

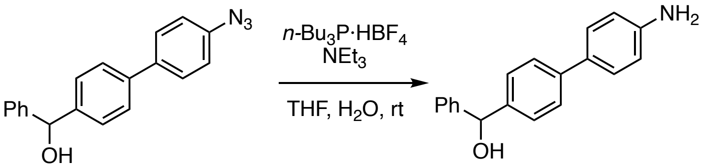

To a solution of (4'-azido-[1,1'-biphenyl]-4-yl)(phenyl)methanol (**8a**) (15.2 mg, 50.5 μmol), triethylamine (8.4 μL, 60 μmol, 1.2 equiv) dissolved in THF (0.20 mL) and water (20 μL) was added tributylphosphonium tetrafluoroborate (14.5 mg, 50.0 μmol, 1.0 equiv) at room temperature. After stirring for 15 h at the same temperature, the mixture was concentrated under reduced pressure. The residue was purified by preparative TLC (CH_2Cl_2/MeOH = 10/1) to give (4'-amino-[1,1'-biphenyl]-4-yl)(phenyl)methanol (**20a**) (10.2 mg, 37.1 μmol, 73%) as a pale yellow solid.

Characterization Data of New Compounds

4'-Azido-[1,1'-biphenyl]-4-carbaldehyde (**7a**),[S4] 4-azidobenzaldehyde (**7b**),[S5] 3-azidobenzaldehyde (**7c**),[S6] 4-azido-3-methylbenzaldehyde (**7d**),[S7] 3-azido-5-methoxybenzaldehyde (**7g**),[S4] (4'-azido-[1,1'-biphenyl]-4-yl)(phenyl)methanol (**8a**),[S5] 4-azido-4'-bromo-1,1'-biphenyl (**8i**),[S8] (4-aminophenyl)(phenyl)methanol (**20b**),[S5] and (3-aminophenyl)(phenyl)methanol (**20d**)[S5] were identical in spectra data with those reported in the literature

4-Azido-4'-iodo-1,1'-biphenyl (**6a**)

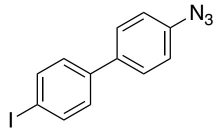

Pale yellow solid; Mp 122–124 °C; TLC R_f 0.65 (*n*-hexane/ EtOAc = 10/1); ^{1}H NMR ($CDCl_3$, 400 MHz) δ 7.07–7.13 (AA'BB', 2H), 7.25–7.33 (AA'BB', 2H), 7.51–7.57 (AA'BB', 2H), 7.74–7.79 (AA'BB', 2H); $^{13}C\{^1H\}$ NMR ($CDCl_3$, 101 MHz): δ 93.1, 119.5, 128.2, 128.6 (two signals overlapped), 136.7, 137.9, 139.6; IR (Nujol, cm^{-1}) 810, 1296, 1306, 1377, 1388, 1463, 1478, 2106, 2139, 2855, 2924, 2953; HRMS (FAB) *m/z*: $[M]^{\cdot+}$ Calcd for $C_{12}H_8IN_3^{\cdot+}$ 320.9763; Found 320.9779.

1-Azido-4-iodo-2-methylbenzene (**6d**)

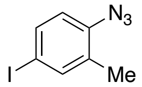

Brown oil; TLC R_f 0.62 (*n*-hexane/EtOAc = 5/1); ^{1}H NMR ($CDCl_3$, 400 MHz): δ 2.15 (s, 3H), 6.85 (d, 1H, *J* = 8.3 Hz), 7.46–7.55 (m, 2H); $^{13}C\{^1H\}$ NMR ($CDCl_3$, 101 MHz): δ 17.0, 88.1, 119.7, 132.0, 135.9, 138.5, 139.8; IR (NaCl, cm^{-1}) 804, 850, 876, 1103, 1158, 1199, 1276, 1294, 1389, 1482, 2073, 2116; HRMS (EI) *m/z*: $[M]^{\cdot+}$ Calcd for $C_7H_6N_3I^{\cdot+}$ 258.9606; Found 258.9604.

4-Azido-2-chloro-1-iodobenzene (**6e**)

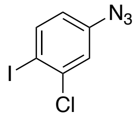

Brown oil; TLC R_f 0.69 (*n*-hexane/EA = 10/1); ^{1}H NMR ($CDCl_3$, 400 MHz): δ 6.69 (dd, 1H, *J* = 8.5, 2.6 Hz), 7.16 (d, 1H, *J* = 2.6 Hz), 7.81 (d, 1H, *J* = 8.5 Hz); $^{13}C\{^1H\}$ NMR ($CDCl_3$, 101 MHz): δ 92.3, 118.9, 120.0, 139.8, 140.9, 141.6; IR (Nujol, cm^{-1}) 804, 1010, 1102, 1243, 1299, 1459, 1579, 2109, 2159; HRMS (EI) *m/z*: $[M]^{\cdot+}$ Calcd for $C_6H_3IClN_3^{\cdot+}$ 278.9060; Found 278.9061.

1-((2-Azidoethoxy)methyl)-4-iodobenzene (**6f**)

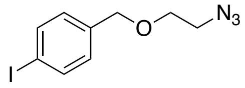

Colorless oil; TLC R_f 0.74 (*n*-hexane/EtOAc =2/1); ^{1}H NMR ($CDCl_3$, 400 MHz): δ 3.41 (t, 2H, *J* = 5.0 Hz), 3.65 (t, 2H, *J* = 5.0 Hz), 4.54 (s, 2H), 7.08–7.15 (AA'BB', 2H), 7.66–7.72 (AA'BB', 2H); $^{13}C\{^1H\}$ NMR ($CDCl_3$, 101 MHz): δ 50.8, 69.1, 72.6, 93.3, 129.5, 137.4, 137.6; IR (NaCl, cm^{-1}) 821, 1007, 1059, 1120, 1286, 1302, 1345, 1359, 1484, 2102, 2862; HRMS (EI) *m/z*: $[M]^{\cdot+}$ Calcd for $C_9H_{10}IN_3O^{\cdot+}$ 302.9869; Found 302.9866.

1-Azido-3-iodo-5-methoxybenzene (**6g**)

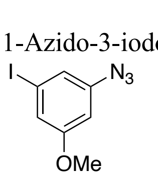

Yellow solid; TLC R_f 0.68 (*n*-hexane/EtOAc = 5/1); ^{1}H NMR ($CDCl_3$, 400 MHz): δ 3.80 (s, 3H), 6.51 (dd, 1H, *J* = 2.0, 2.0 Hz), 7.00 (dd, 1H, *J* = 2.0, 1.4 Hz), 7.04 (dd, 1H, *J* = 2.0, 1.4 Hz); $^{13}C\{^1H\}$ NMR ($CDCl_3$, 101 MHz): δ 55.6, 94.5, 105.0, 119.9, 120.4, 142.3, 161.0; IR (NaCl, cm^{-1}) 824, 1045, 1228, 1291, 1316, 1422, 1455, 1567, 1591, 2109, 2939; HRMS (EI) *m/z*: $[M]^{\cdot+}$ Calcd for $C_7H_6IN_3O^{\cdot+}$ 274.9557; Found 274.9556.

4-Azido-2-chlorobenzaldehyde (**7e**)

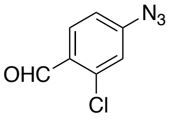

Pale yellow solid; Mp 54–55 °C; TLC R_f 0.76 (*n*-hexane/EtOAc = 5/1); ^{1}H NMR ($CDCl_3$, 400 MHz): δ 7.04 (ddd, 1H, *J* = 8.4, 2.1, 0.7 Hz), 7.10 (d, 1H, *J* = 2.1 Hz), 7.93 (d, 1H, *J* = 8.4 Hz), 10.37 (d, 1H, *J* = 0.7 Hz); ^{13}C{^{1}H} NMR ($CDCl_3$, 101 MHz): δ 118.0, 120.6, 129.2, 131.0, 139.5, 147.0, 188.3; IR (Nujol, cm^{-1}) 1256, 1262, 1279, 1306, 1591, 1683,1688, 1698, 2122; HRMS (ESI) *m/z*: [M+Na]$^+$ Calcd for $C_7H_3ClN_3NaO^+$ 179.9965; Found 179.9961.

4-((2-Azidoethoxy)methyl)benzaldehyde (**7f**)

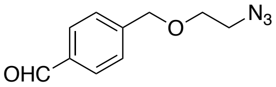

Yellow oil; TLC R_f 0.36 (*n*-hexane/EtOAc = 3/1); ^{1}H NMR ($CDCl_3$, 400 MHz): δ 3.46 (t, 2H, *J* = 4.9 Hz), 3.72 (t, 2H, *J* = 4.9 Hz), 4.67 (s, 2H), 7.50–7.56 (AA'BB', 2H), 7.86–7.91 (AA'BB', 2H), 10.02 (s, 1H); ^{13}C{^{1}H} NMR ($CDCl_3$, 101 MHz): δ 50.8, 69.5, 72.5, 127.6, 129.9, 135.8, 144.8, 191.9; IR (Nujol, cm^{-1}) 816, 1103, 1118, 1166, 1211, 1305, 1610, 1698, 1703, 2105; HRMS (EI) *m/z*: [M]$^{\cdot+}$ Calcd for $C_{10}H_{11}N_3O_2^{\cdot+}$ 206.0930; Found 206.0931.

(4'-Azido-[1,1'-biphenyl]-4-yl)(4-chlorophenyl)methanol (**8b**)

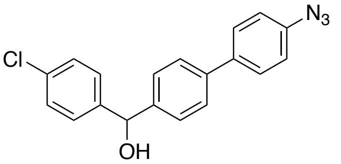

Pale yellow solid; Mp 104–105 °C; TLC R_f 0.35 (*n*-hexane/EtOAc = 5/1); ^{1}H NMR ($CDCl_3$, 400 MHz): δ 2.43 (br s, 1H), 5.82 (s, 1H), 7.05–7.10 (AA'BB', 2H), 7.28–7.36 (m, 4H), 7.37–7.42 (AA'BB', 2H), 7.49–7.57 (m, 4H); ^{13}C{^{1}H} NMR ($CDCl_3$, 101 MHz): δ 75.4, 119.5, 127.07, 127.10, 127.9, 128.4, 128.7, 133.4, 137.4, 139.3, 139.7, 142.1, 142.6; IR (Nujol, cm^{-1}) 811, 1012, 1032, 1095, 1173, 1292, 1368, 1488, 1515, 1598, 2093, 2125; HRMS (ESI) *m/z*: [M–H]$^-$ Calcd for $C_{19}H_{13}ClN_3O^-$ 334.0747; Found 334.0750.

(4'-Azido-[1,1'-biphenyl]-4-yl)(4-methoxyphenyl)methanol (**8c**)

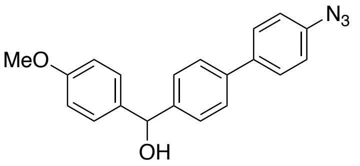

Pale yellow solid; Mp 112–113 °C; TLC R_f 0.29 (*n*-hexane/EtOAc = 5/1); ^{1}H NMR ($CDCl_3$, 400 MHz): δ 2.25–2.38 (br, 1H), 3.78 (s, 3H), 5.83 (s, 1H), 6.83–6.91 (AA'BB', 2H), 7.04–7.10 (AA'BB', 2H), 7.24–7.34 (AA'BB', 2H), 7.39–7.47 (AA'BB', 2H), 7.47–7.58 (m, 4H); ^{13}C{^{1}H} NMR ($CDCl_3$, 101 MHz): δ 55.3, 75.5, 113.9, 119.4, 126.5, 126.9, 127.9, 128.3, 136.0, 137.5, 139.1, 139.2, 143.2, 159.1; IR (Nujol, cm^{-1}) 1009, 1033, 1168, 1245, 1295, 1581, 1610, 2090, 2125, 3427; HRMS (ESI) *m/z*: [M+Na]$^+$ Calcd for $C_{20}H_{17}N_3NaO_2^+$ 354.1219; Found 354.1217.

(4'-Azido-[1,1'-biphenyl]-4-yl)(naphthalen-2-yl)methanol (**8d**)

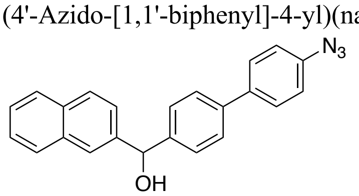

Pale yellow solid; Mp 143–144 °C; TLC R_f 0.69 (*n*-hexane/EtOAc = 2/1); ^{1}H NMR ($CDCl_3$, 400 MHz): δ 2.36 (d, 1H, *J* = 3.4 Hz), 6.06 (d, 1H, *J* = 3.4 Hz), 7.06–7.11 (AA'BB', 2H), 7.44–7.59 (m, 9H), 7.79–7.89 (m, 3H), 7.93 (s, 1H); ^{13}C{^{1}H} NMR ($CDCl_3$, 101 MHz): δ 76.1, 119.4, 124.7, 125.0, 126.1, 126.3, 127.0, 127.2, 127.7, 128.1, 128.4, 128.5, 132.9, 133.2, 137.5, 139.2, 139.5, 141.0, 142.8; IR (Nujol, cm^{-1}) 816, 1016, 1030, 1272, 1295, 1600, 2089, 2119, 3407; HRMS (ESI) *m/z*: [M+Na]$^+$ Calcd for $C_{23}H_{17}N_3NaO^+$ 374.1269; Found 374.1271.

(4'-Azido-[1,1'-biphenyl]-4-yl)(thiophen-2-yl)methanol (**8e**)

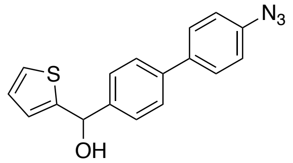

Pale yellow solid; Mp 96–97 °C; TLC R_f 0.40 (*n*-hexane/EtOAc = 5/1); ^{1}H NMR ($CDCl_3$, 400 MHz): δ 2.35–2.65 (br, 1H), 6.14 (s, 1H), 6.94–7.01 (m, 2H), 7.09–7.15 (AA'BB', 2H), 7.31 (dd, 1H, J = 5.0, 1.3 Hz), 7.52–7.64 (m, 6H); ^{13}C{^{1}H} NMR ($CDCl_3$, 101 MHz): δ 72.2, 119.5, 125.0, 125.6, 126.8, 126.9, 127.0, 128.4, 137.5, 139.3, 139.8, 142.3, 148.0; IR (Nujol, cm^{-1}) 813, 1261, 1271, 1295, 2092, 2112, 3335, 3348, 3358, 3364, 3374, 3394, 3417; HRMS (ESI) *m/z*: [M+Na]$^+$ Calcd for $C_{17}H_{13}N_3NaOS^+$ 330.0677; Found 330.0678.

2-(4'-Azido-[1,1'-biphenyl]-4-yl)propan-2-ol (**8f**)

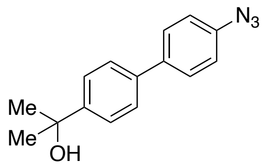

Pale yellow solid; Mp 89–90 °C; TLC R_f 0.46 (*n*-hexane/EtOAc = 5/1); ^{1}H NMR ($CDCl_3$, 400 MHz): δ 1.63 (s, 6H), 1.75–1.82 (br, 1H), 7.07–7.13 (AA'BB', 2H), 7.52–7.62 (m, 6H); ^{13}C{^{1}H} NMR ($CDCl_3$, 101 MHz): δ 31.8, 72.4, 119.4, 125.0, 126.7, 128.3, 137.5, 138.5, 139.0, 148.3; IR (Nujol, cm^{-1}) 866, 957, 1097, 1130, 1165, 1295, 1600, 2093, 3307; HRMS (EI) *m/z*: [M]$^{\cdot+}$ Calcd for $C_{15}H_{15}N_3O^{\cdot+}$ 253.1215; Found 253.1215.

1-(4'-Azido-[1,1'-biphenyl]-4-yl)-2,2,2-trifluoro-1-phenylethan-1-ol (**8g**)

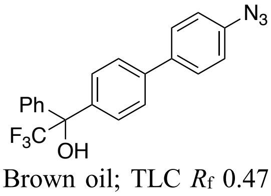

Brown oil; TLC R_f 0.47 (*n*-hexane/EtOAc = 5/1); ^{1}H NMR ($CDCl_3$, 400 MHz): δ 2.95 (br s, 1H), 7.07–7.12 (AA'BB', 2H), 7.35–7.40 (m, 3H), 7.50−7.59 (m, 8H); ^{13}C{^{1}H} NMR ($CDCl_3$, 101 MHz): δ 29.7, 79.3 (q, J_{C-F} = 28.7 Hz), 119.5, 123.9, 126.6, 125.3 (q, J_{C-F} = 287 Hz), 127.4 (q, J_{C-F} = 1.6 Hz), 128.0 (q, J_{C-F} = 1.2 Hz), 128.4, 128.8, 137.0, 138.4, 139.2, 139.6, 140.4; ^{19}F NMR ($CDCl_3$, 376 MHz) δ –74.3 (s); IR (NaCl, cm^{-1}) 1010, 1102, 1133, 1243, 1263, 1296, 1435, 1439, 1459, 1157, 1564, 1568, 1580, 2106, 2148, 2159; HRMS (ESI) *m/z*: [M–H]$^-$ Calcd for $C_{20}H_{13}F_3N_3O^-$ 368.1002; Found 368.1011.

4-Allyl-4'-azido-1,1'-biphenyl (**8h**)

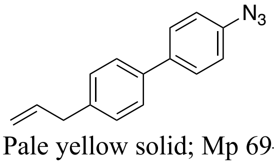

Pale yellow solid; Mp 69–70 °C; TLC R_f 0.66 (*n*-hexane/EA = 5/1); ^{1}H NMR ($CDCl_3$, 400 MHz): δ 3.43 (d, 2H, J = 6.7 Hz), 5.02–5.18 (m, 2H), 5.91–6.08 (m, 1H), 7.04–7.13 (AA'BB', 2H), 7.22–7.30 (AA'BB', 2H), 7.46–7.53 (AA'BB', 2H), 7.53–7.62 (AA'BB', 2H); ^{13}C{^{1}H} NMR ($CDCl_3$, 101MHz): δ 39.9, 116.1, 119.4, 126.9, 128.3, 129.1, 137.3, 137.8, 138.0, 138.9, 139.4; IR (Nujol, cm^{-1}) 801, 827, 834, 915, 993, 1115, 1130, 1293, 1402, 1515, 1602, 1640, 2093, 2125, 2261, 2415; HRMS (EI) *m/z*: [M]$^{\cdot+}$ Calcd for $C_{15}H_{13}N_3^{\cdot+}$ 235.1109; Found 235.1110 .

4'-(4-(*p*-Tolyl)-1*H*-1,2,3-triazol-1-yl)-[1,1'-biphenyl]-4-carbaldehyde (**16a**)

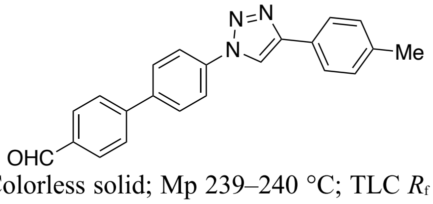

Colorless solid; Mp 239–240 °C; TLC R_f 0.76 (CH_2Cl_2/MeOH = 10/1); ^{1}H NMR ($CDCl_3$, 400 MHz): δ 2.42 (s, 3H), 7.25–7.33 (AA'BB', 2H), 7.77–7.86 (m, 6H), 7.91–7.97 (AA'BB', 2H), 7.99–8.05 (AA'BB', 2H), 8.23 (s, 1H), 10.10 (s, 1H); ^{13}C{^{1}H} NMR ($CDCl_3$, 101 MHz): δ 21.4, 117.0, 120.9, 125.8, 127.7, 127.8, 128.8, 129.7,

130.5, 135.6, 137.1, 138.5, 140.1, 145.5, 148.7, 191.9; IR (NaCl, cm^{-1}) 811, 1042, 1092, 1681, 1697, 2928, 3105; HRMS (ESI) *m/z*: $[M+Na]^+$ Calcd for $C_{22}H_{17}N_3NaO^+$ 362.1269; Found 362.1269.

4'-((5*aS**,6*S**,6*aR**)-6-(Hydroxymethyl)-5,5a,6,6a,7,8-hexahydrocyclopropa[5,6]cycloocta[1,2-*d*][1,2,3]triazol-1(4*H*)-yl)-[1,1'-biphenyl]-4-carbaldehyde (**16b**)

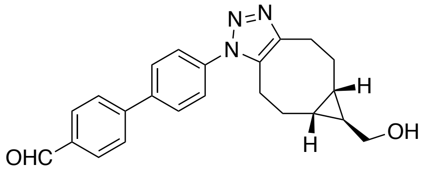

Pale yellow solid; Mp 153–155 °C; TLC R_f 0.57 (CH_2Cl_2/MeOH = 10/1); ^1H NMR ($CDCl_3$, 400 MHz): δ 0.77–1.00 (m, 3H), 1.38–1.50 (m, 3H), 2.39–2.56 (m, 2H), 2.66–2.70 (m, 1H), 2.89–3.00 (m, 2H), 3.45–3.61 (m, 2H), 7.50–7.56 (AA'BB', 2H), 7.77–7.85 (m, 4H), 7.99–8.06 (AA'BB', 2H), 10.11 (s, 1H); $^{13}C\{^1H\}$ NMR ($CDCl_3$, 101 MHz): δ 22.5 (two signals overlapped), 23.4, 25.8, 27.3, 27.6, 28.1, 66.4, 126.4, 127.9, 128.4, 130.5, 134.6, 135.7, 136.7, 140.9, 145.56, 145.63, 191.8; IR (NaCl, cm^{-1}) 821, 841, 1004, 1029, 1095, 1170, 1215, 1500, 1527, 1605, 1683, 1698, 2857, 2925, 3383; HRMS (ESI) *m/z*: $[M+Na]^+$ Calcd for $C_{23}H_{23}N_3NaO_2^+$ 396.1688; Found 396.1691.

4'-(1*H*-Benzo[*d*][1,2,3]triazol-1-yl)-[1,1'-biphenyl]-4-carbaldehyde (**16c**)

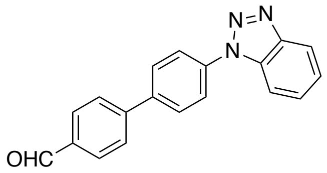

Pale yellow solid; TLC R_f 0.56 (*n*-hexane/EtOAc = 1/1); ^1H NMR ($CDCl_3$, 400 MHz): δ 7.48 (ddd, 1H, J = 1.0, 8.3, 8.3 Hz), 7.61, (ddd, 1H, J = 8.3, 1.4 Hz) 7.80–7.87 (m, 3H), 7.87–7.92 (AA'BB', 2H), 7.93–7.98 (AA'BB', 2H), 8.00–8.06 (AA'BB', 2H), 8.19 (dd, 1H, J = 8.3, 1.4 Hz) 10.11 (s, 1H); $^{13}C\{^1H\}$ NMR ($CDCl_3$, 101 MHz): δ 110.3, 120.6, 123.2, 124.6, 127.8, 128.5, 128.9, 130.5, 132.2, 135.6, 137.1, 140.0, 145.6, 146.7, 191.9; IR (Nujol, cm^{-1}) 811, 1007, 1062, 1166, 1176, 1218, 1525, 1560, 1605, 1698; HRMS (ESI) *m/z*: $[M+Na]^+$ Calcd for $C_{19}H_{13}N_3NaO^+$ 322.0955; Found 322.0956.

1-(4'-Azido-[1,1'-biphenyl]-4-yl)-*N*-methylmethanamine (**19**)

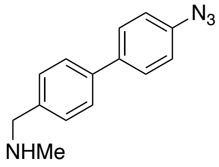

Pale yellow solid; Mp 104–105 °C; TLC R_f 0.34 (EtOAc); ^1H NMR ($CDCl_3$, 400 MHz): δ 2.48 (s, 3H), 3.79 (s, 2H), 7.06–7.12 (AA'BB', 2H), 7.36–7.43 (AA'BB', 2H), 7.50–7.55 (AA'BB', 2H), 7.55–7.61 (AA'BB', 2H); $^{13}C\{^1H\}$ NMR ($CDCl_3$, 101MHz): δ 36.1, 55.7, 119.4, 126.9, 128.4, 128.7, 137.7, 138.8, 139.0, 139.4; HRMS (ESI) *m/z*: $[M+Na]^+$ Calcd for $C_{14}H_{15}N_4^+$ 239.1300; Found 239.1300.

(4'-Amino-[1,1'-biphenyl]-4-yl)(phenyl)methanol (**20a**)

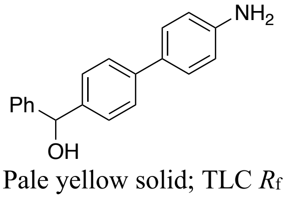

Pale yellow solid; TLC R_f 0.56 (CH_2Cl_2/MeOH = 10/1); ^1H NMR ($CDCl_3$, 400 MHz): δ 2.23 (br s, 1H), 3.73 (br s, 2H), 5.88 (s, 1H), 6.71–7.77 (AA'BB', 2H), 7.26–7.30 (AA'BB'C, 1H), 7.32–7.45 (m, 8H), 7.48–7.53 (AA'BB', 2H); $^{13}C\{^1H\}$ NMR ($CDCl_3$, 101MHz): δ 76.1, 115.4, 126.49, 126.53, 126.9, 127.6, 128.0, 128.5, 131.1, 140.5, 141.8, 143.8, 145.9; IR (NaCl, cm^{-1}) 803, 1182, 1498, 1611, 1621, 3341, 3353, 3364, 3373; HRMS (ESI) *m/z*: $[M+Na]^+$ Calcd for $C_{19}H_{17}NNaO^+$ 298.1202; Found 298.1208.

(4-Azido-3-methylphenyl)(phenyl)methanol (**S1**)

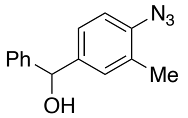

Pale yellow solid; Mp 64–65 °C; TLC R_f 0.31 (*n*-hexane/EtOAc = 5/1); ^{1}H NMR ($CDCl_3$, 400 MHz): δ 2.15–2.22 (br, 4H), 5.80 (br s, 1H), 7.08 (d, 1H, *J* = 8.2 Hz), 7.18 (d, 1H, *J* = 1.8 Hz), 7.24 (dd, 1H, *J* = 8.2, 1.8 Hz), 7.26–7.30 (m, 1H), 7.31–7.39 (m, 4H); $^{13}C\{^1H\}$ NMR ($CDCl_3$, 101 MHz): δ 17.4, 75.8, 118.0, 125.3, 126.4, 127.7, 128.6, 129.3, 129.7, 137.7, 140.2, 143.7; IR (NaCl, cm^{-1}) 811, 1023, 1036, 1096, 1160, 1293, 1452, 1497, 2078, 2122, 3353; HRMS (ESI) *m/z*: $[M–H]^-$ Calcd for $C_{14}H_{12}N_3O^+$ 238.0980; Found 238.0981.

(4-Amino-3-methylphenyl)(phenyl)methanol (**20c**)

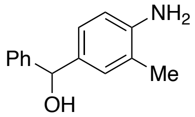

Pale yellow solid; Mp 123–124 °C; TLC R_f 0.47 (*n*-hexane/EtOAc = 1/1); ^{1}H NMR ($CDCl_3$, 400 MHz): δ 2.11–2.23 (br, 4H), 3.59 (br s, 2H), 5.74 (br s, 1H), 6.62 (d, 1H, *J* = 8.0 Hz), 7.01 (d, 1H, *J* = 8.0 Hz), 7.04 (s, 1H), 7.22–7.38 (AA'BB'C, 1H), 7.30–7.36 (AA'BB'C, 2H), 7.36–7.42 (AA'BB'C, 2H); $^{13}C\{^1H\}$ NMR ($CDCl_3$, 101 MHz): δ 17.5, 76.1, 114.8, 122.4, 125.6, 126.3, 127.2, 128.4, 129.0, 134.2, 144.1, 144.2; IR (NaCl, cm^{-1}) 801, 806, 1027, 1265, 1455, 1495, 1505, 1621, 2849, 2918, 3334; HRMS (ESI) *m/z*: $[M+Na]^+$ Calcd for $C_{14}H_{15}NNaO^+$ 236.1046; Found 236.1054.

(3-Azido-5-methoxyphenyl)(thiophen-2-yl)methanol (**S2**)

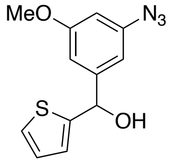

Pale yellow solid; Mp 90–91 °C; TLC R_f 0.30 (*n*-hexane/EtOAc = 5/1); ^{1}H NMR ($CDCl_3$, 400 MHz): δ 2.47 (d, 1H, *J* = 3.7 Hz), 3.80 (s, 3H), 6.00 (d, 1H, *J* = 3.7 Hz), 6.48 (dd, 1H, *J* = 2.2, 2.2 Hz), 6.75 (dd, 1H, *J* = 2.2, 0.5 Hz), 6.79 (dd, 1H, *J* = 2.2, 0.5 Hz), 6.91–6.97 (m, 2H), 7.28 (dd, 1H, *J* = 5.0, 1.3 Hz); $^{13}C\{^1H\}$ NMR ($CDCl_3$, 101 MHz): δ 35.5, 71.9, 104.4, 108.6, 109.2, 125.2, 125.8, 126.8, 141.5, 146.2, 147.3, 160.9; IR (NaCl, cm^{-1}) 1037, 1057, 1239, 1433, 1455, 1464, 1595, 2111, 3341; HRMS (ESI) *m/z*: $[M–H]^-$ Calcd for $C_{12}H_{10}N_3O_2S^+$ 260.0494; Found 260.0494.

Methyl 1-(3-(hydroxy(thiophen-2-yl)methyl)-5-methoxyphenyl)-1*H*-1,2,3-triazole-4-carboxylate (**21a**)

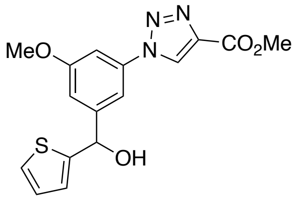

Pale yellow solid; Mp 149–150 °C; TLC R_f 0.57 (CH_2Cl_2/MeOH = 10/1); ^{1}H NMR ($CDCl_3$, 400 MHz): δ 2.68 (d, 1H, *J* = 3.2 Hz), 3.90 (s, 3H), 3.99 (s, 3H), 6.13 (d, 1H, *J* = 3.2 Hz), 6.95–7.00 (m, 2H), 7.11–7.15 (m, 1H), 7.28–7.32 (m, 2H), 7.37–7.40 (m, 1H), 8.51 (s, 1H); $^{13}C\{^1H\}$ NMR ($CDCl_3$, 101 MHz): δ 52.6, 55.9, 71.5, 105.9, 110.3, 113.2, 125.5, 125.7, 126.2, 126.9, 137.3, 140.5, 146.7, 146.9, 160.8, 161.0; IR (NaCl, cm^{-1}) 1037, 1146, 1168, 1222, 1435, 1608, 1728, 2950, 3136, 3413; HRMS (ESI) *m/z*: $[M+Na]^+$ Calcd for $C_{16}H_{15}N_3NaO_4S^+$ 368.0681; Found 368.0681.

(3-Methyl-4-(4-(*p*-tolyl)-1*H*-1,2,3-triazol-1-yl)phenyl)(phenyl)methanol (**21b**)

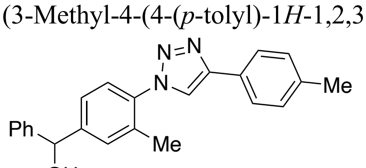

Pale yellow solid; Mp 89–90 °C; TLC R_f 0.54 (CH_2Cl_2/MeOH = 10/1); ^{1}H NMR ($CDCl_3$, 400 MHz): δ 2.24 (s, 3H), 2.40 (s, 3H), 2.41–2.60 (br, 1H), 5.88–5.91 (br, 1H), 7.23–7.45 (m, 10H), 7.78 (d, 2H, *J* = 8.2 Hz), 7.88 (s,

1H); $^{13}C\{^1H\}$ NMR ($CDCl_3$, 101 MHz): δ 18.1, 21.4, 75.7, 120.8, 124.9, 125.7, 126.1, 126.6, 127.5, 128.0, 128.8, 129.4, 129.6, 133.9, 135.6, 138.3, 143.4, 145.6, 147.7; IR (NaCl, cm^{-1}) 806, 817, 994, 1040, 1229, 1265, 1452, 1495, 1510, 3378; HRMS (ESI) *m/z*: $[M+Na]^+$ Calcd for $C_{23}H_{21}N_3NaO^+$ 378.1580; Found 378.1582.

2-(4-Azidophenyl)propan-2-ol (**S3**)

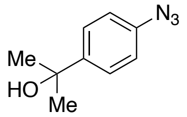

Yellow oil; TLC R_f 0.35 (*n*-hexane/EtOAc = 5/1); ^1H NMR ($CDCl_3$, 400 MHz): δ 1.57 (s, 6H), 1.73–1.83 (br, 1H), 6.95–7.04 (AA'BB', 2H) 7.43–7.52 (AA'BB', 2H); $^{13}C\{^1H\}$ NMR ($CDCl_3$, 101 MHz): δ 31.8, 72.3, 118.8, 126.0, 138.4, 145.9; IR (NaCl, cm^{-1}) 833, 1133, 1170, 1286,1294, 1508, 2090, 2126, 2976, 3381; HRMS (ESI) *m/z*: $[M]^{\cdot+}$ Calcd for $C_6H_{11}N_3O^{\cdot+}$ 177.0902; Found 177.0902.

(8*R*,9*S*,13*S*,14*S*,17*S*)-17-(1-(4-(2-Hydroxypropan-2-yl)phenyl)-1*H*-1,2,3-triazol-4-yl)-13-methyl-7,8,9,11,12,13,14,15,16,17-decahydro-6*H*-cyclopenta[*a*]phenanthrene-3,17-diol (**21c**)

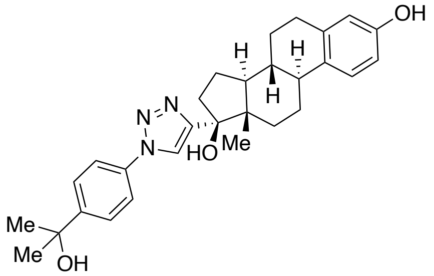

Pale yellow solid; Mp 153–155 °C; TLC R_f 0.35 (CH_2Cl_2/MeOH = 10/1); ^1H NMR ($CDCl_3$, 400 MHz): δ 0.76–0.86 (m, 1H), 1.08 (s, 3H), 1.19–1.74 (m, 11H), 1.86 (br s, 1H), 1.87–2.04 (m, 3H), 2.11–2.23 (m, 2H), 2.46–2.57 (m, 1H), 2.75 (s, 1H), 2.78–2.90 (m, 2H), 3.49 (br s, 1H), 4.68–5.01 (br, 1H), 6.55 (d, 1H, *J* = 2.7 Hz), 6.59 (dd, 1H, *J* = 8.4, 2.7 Hz), 7.05 (d, 1H, *J* = 8.4 Hz), 7.61–7.68 (AA'BB', 2H), 7.69–7.76 (AA'BB', 2H), 7.88 (s, 1H); $^{13}C\{^1H\}$ NMR ($CDCl_3$, 101 MHz): δ 14.3, 23.5, 26.3, 27.3, 29.7, 31.9, 33.0, 38.1, 39.5, 43.3, 47.4, 48.5, 72.4, 82.5, 112.6, 115.2, 119.4, 120.2, 125.9, 126.5, 132.6, 135.7, 138.3, 149.9, 153.3, 154.3; IR (NaCl, cm^{-1}) 1232, 1286, 1504, 1511, 1514, 2870, 2929, 2973, 3156, 3348, 3354, 3360, 3367, 3374, 3388, 3507; HRMS (ESI) *m/z*: $[M+Na]^+$ Calcd for $C_{29}H_{35}N_3NaO_3^+$ 472.2600; Found 472.2601.

References for Supporting Information

S1 T. B. Silva, K. N. K. Ji, F. P. Pauli, R. M. S. Galvão, A. F. M. Faria, M. L. Bello, J. A. L. C. Resende, V. R. Campos, L. da S. M. Forezi, F. de C. da Silva, R. X. Faria, V. F. Ferreira, *Bioorganic Chem.* **2021**, *116*, 105250.

S2 N. Faucher, Y. Ambroise, J.-C. Cintrat, E. Doris, F. Pillon, B. Rousseau, *J. Org. Chem*. **2002**, *67*, 932.

S3 S. Yoshida, Y. Misawa, T. Hosoya, *Eur. J. Org. Chem.* **2014**, 3991.

S4 T. Aimi, T. Meguro, A. Kobayashi, T. Hosoya, S. Yoshida, *Chem. Commun*. **2021**, *57*, 6062.

S5 D. Ichinari, Y. Ashikari, K. Mandai, Y. Aizawa, J.-i. Yoshida, *Angew. Chem., Int. Ed.* **2020**, *59*, 1567.

S6 P. M. Lahti, B. Esat, Y. Liao, P. Serwinski, J. Lan, R. Walton, *Polyhedron* **2001**, *20*, 1647.

S7 I. N. Zhmurova, A. A. Tukhar, R. I. Yurchenko, *Zhurnal Obshchei Khimii* **1969**, *39*, 2201.

S8 A. R. Hajipour, F. Mohammadsaleh, *Tetrahedron Lett.* **2014**, *55*, 6799.

^{1}H and ^{13}C NMR Spectra of Compounds

^{1}H NMR (400 MHz) and ^{13}C NMR (101 MHz) spectra of 4-azido-4'-iodo-1,1'-biphenyl (**6a**) ($CDCl_3$)

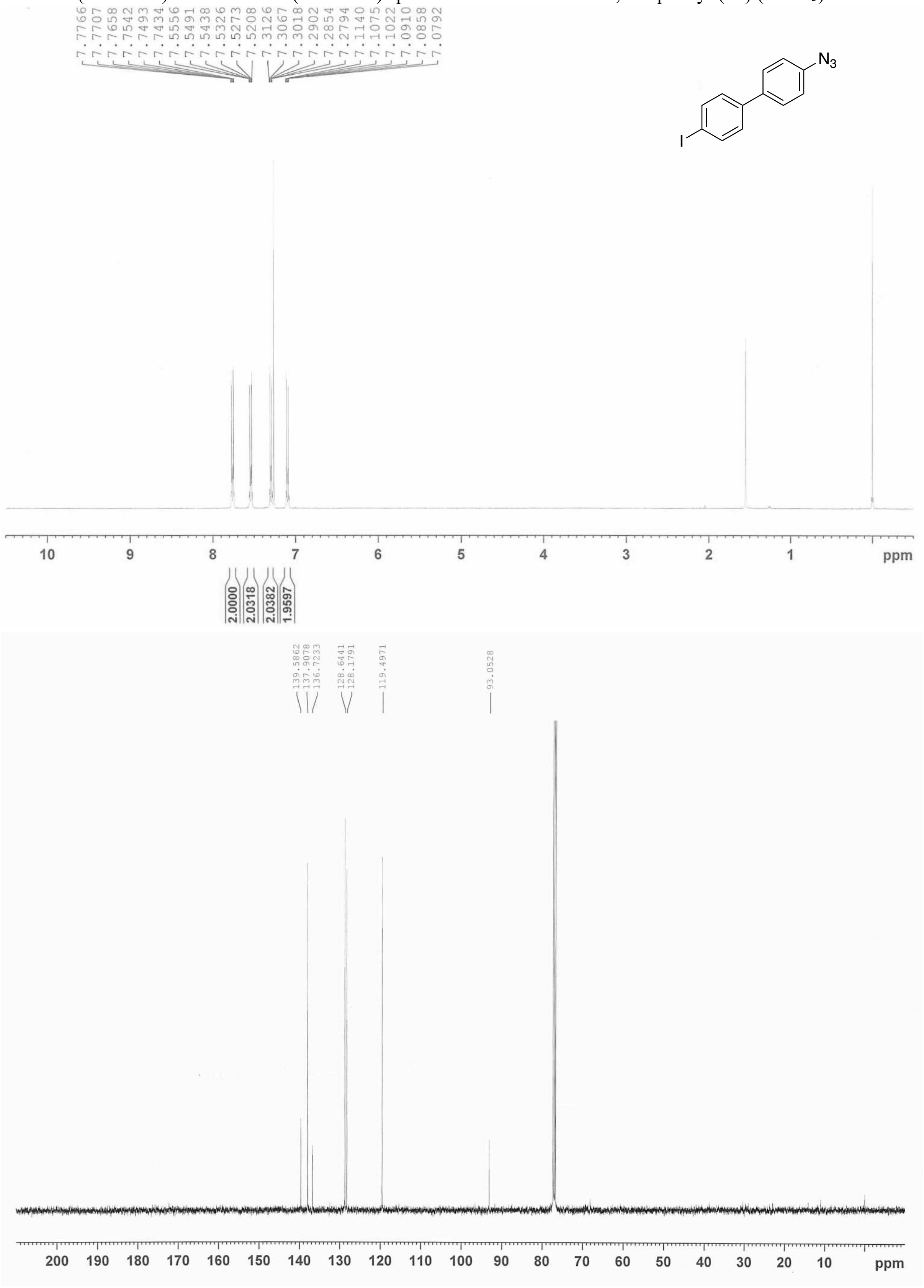

^{1}H NMR (400 MHz) and ^{13}C NMR (101 MHz) spectra of 1-azido-4-iodo-2-methylbenzene (**6d**) ($CDCl_3$)

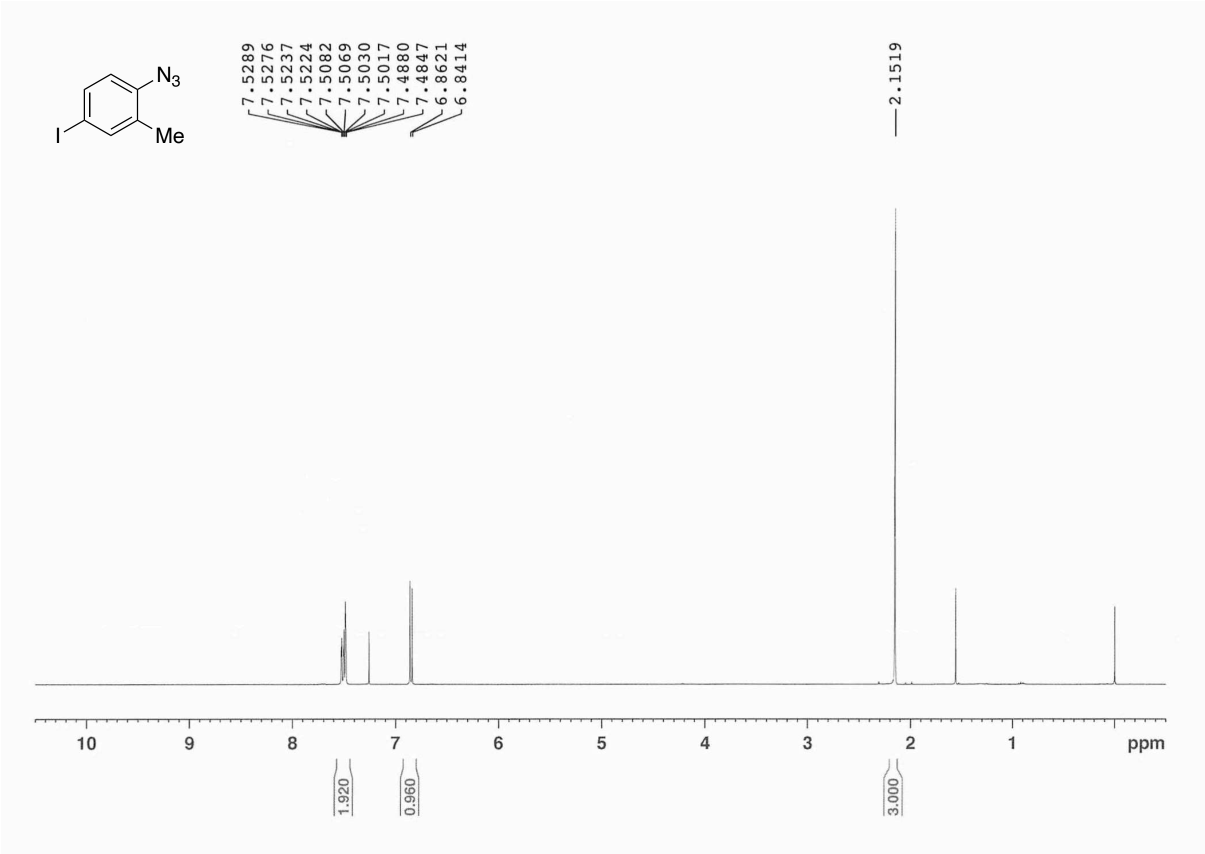

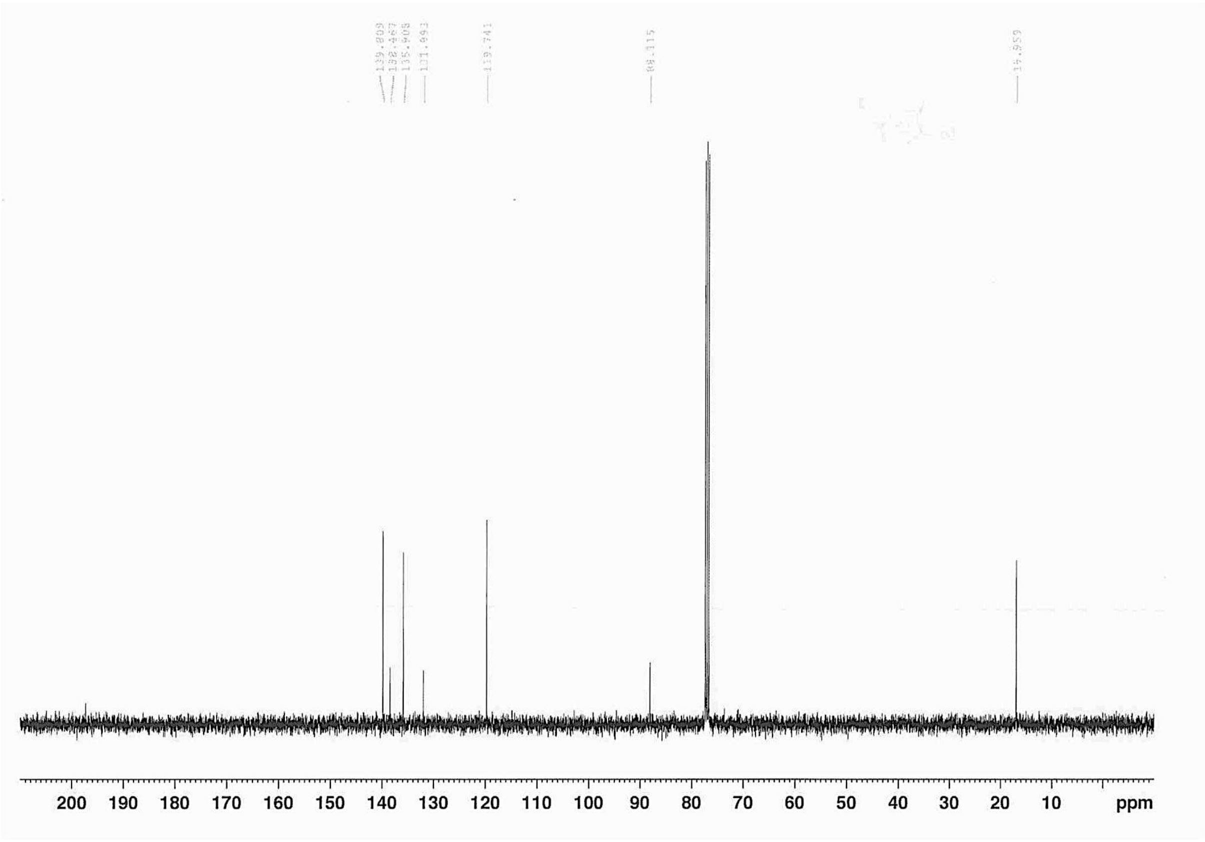

^{1}H NMR (400 MHz) and ^{13}C NMR (101 MHz) spectra of 4-azido-2-chloro-1-iodobenzene (**6e**) ($CDCl_3$)

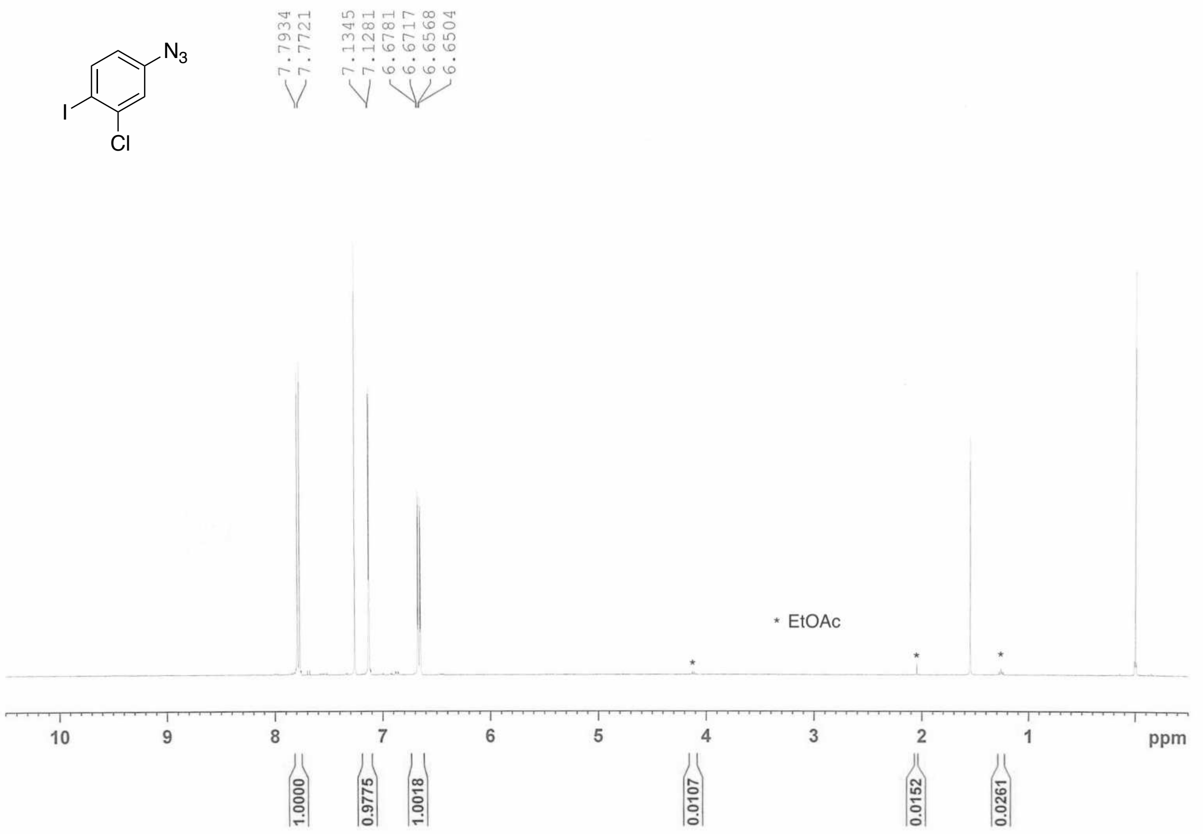

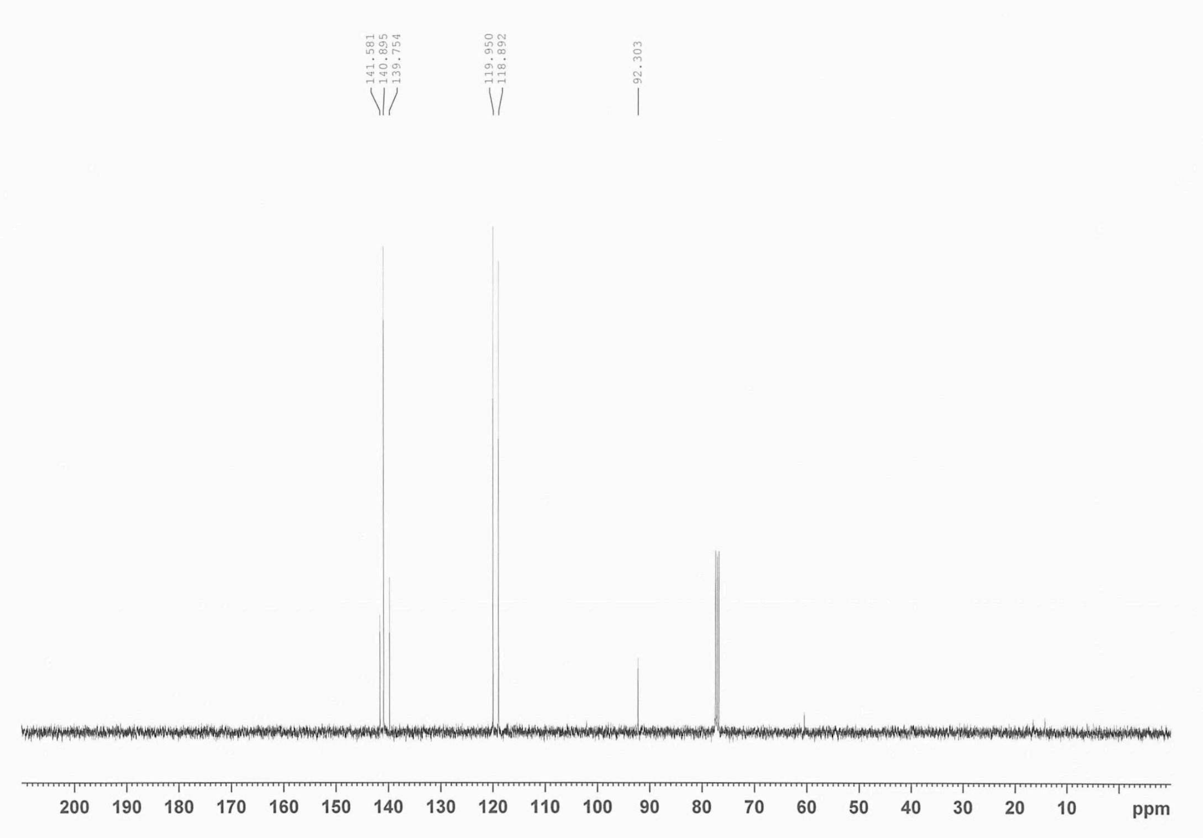

^{1}H NMR (400 MHz) and ^{13}C NMR (101 MHz) spectra of 1-((2-azidoethoxy)methyl)-4-iodobenzene (**6f**) ($CDCl_3$)

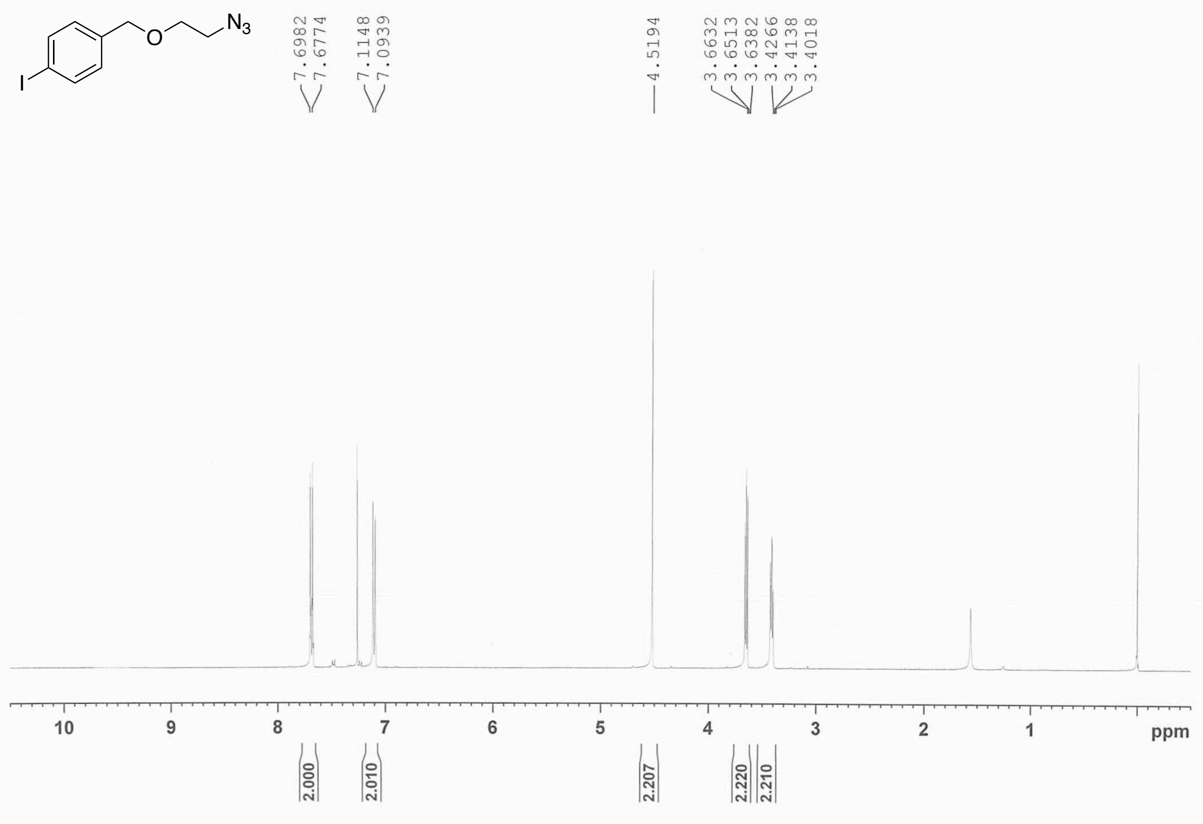

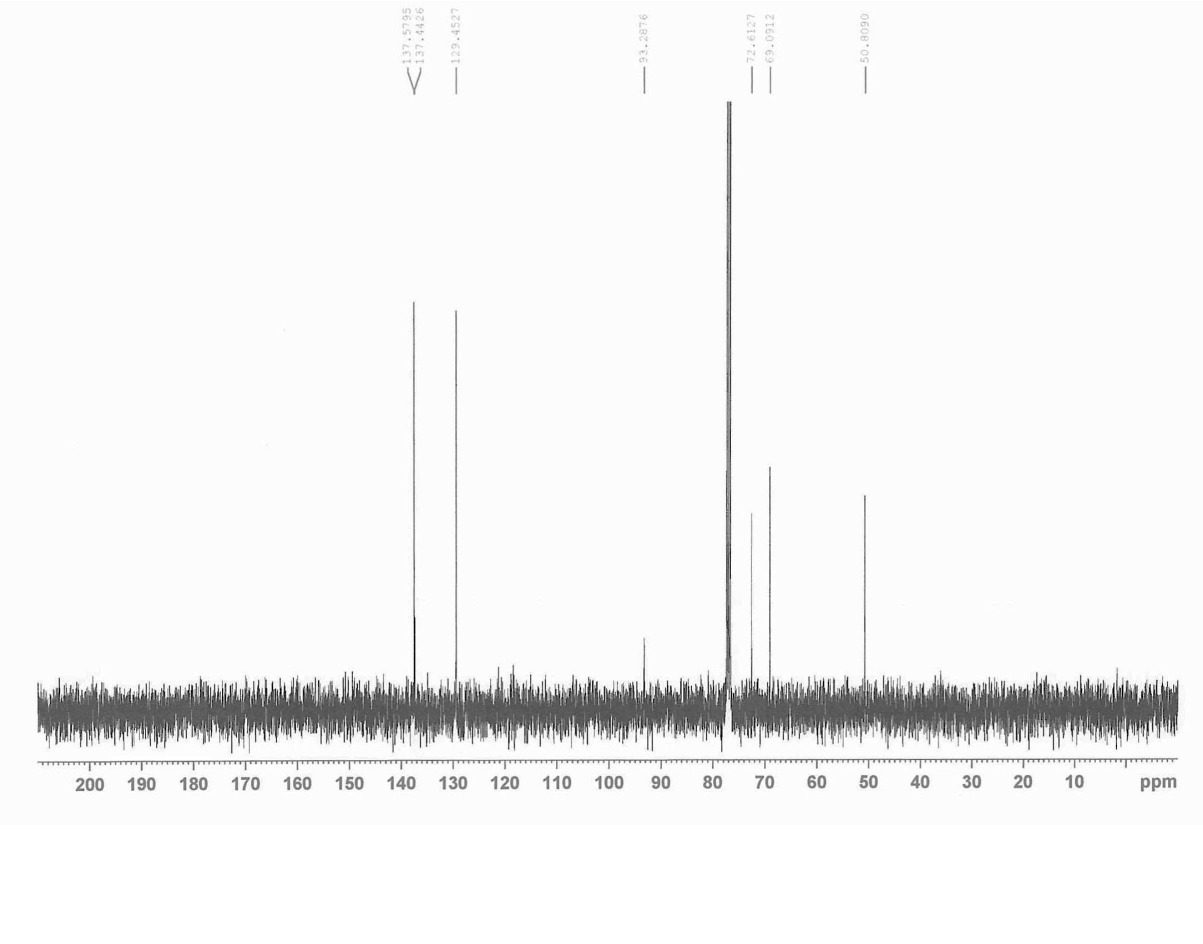

^{1}H NMR (400 MHz) and ^{13}C NMR (101 MHz) spectra of 1-azido-3-iodo-5-methoxybenzene (**6g**) ($CDCl_3$)

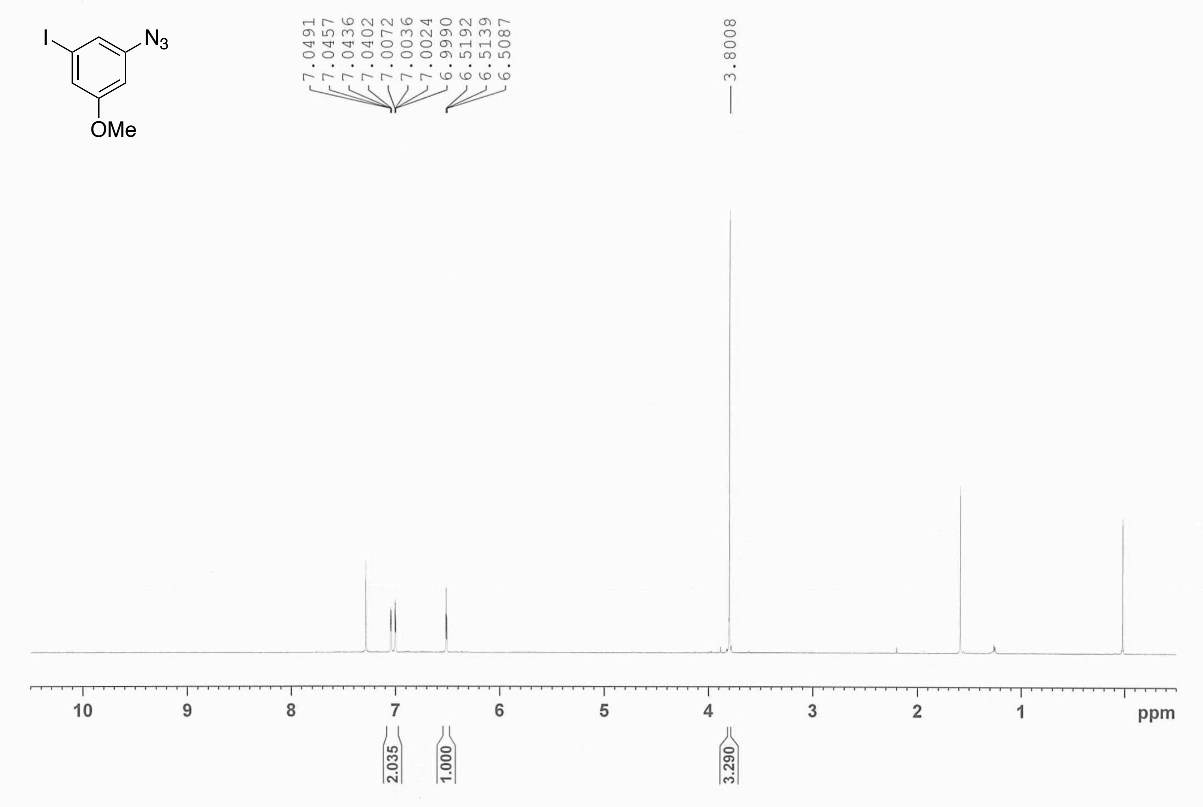

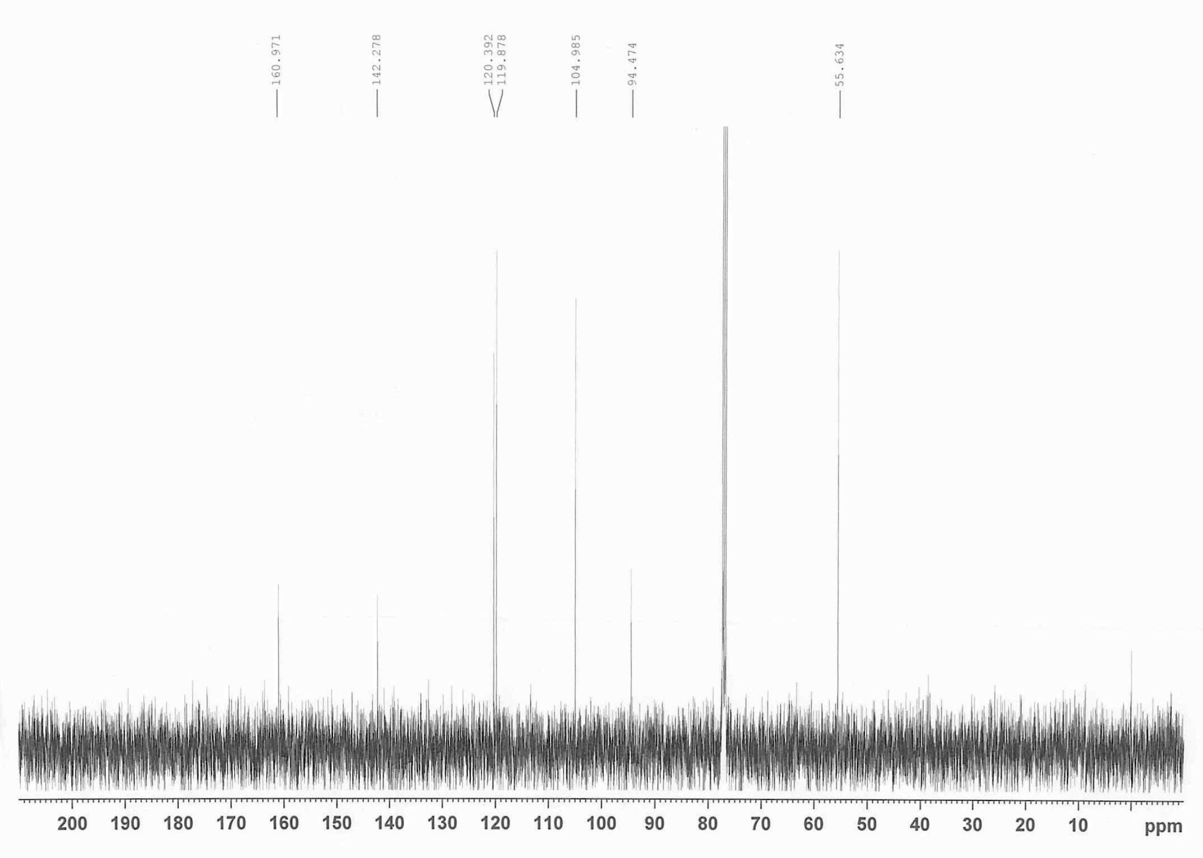

^{1}H NMR (400 MHz) and ^{13}C NMR (101 MHz) spectra of 4-azido-2-chlorobenzaldehyde (**7e**) ($CDCl_3$)

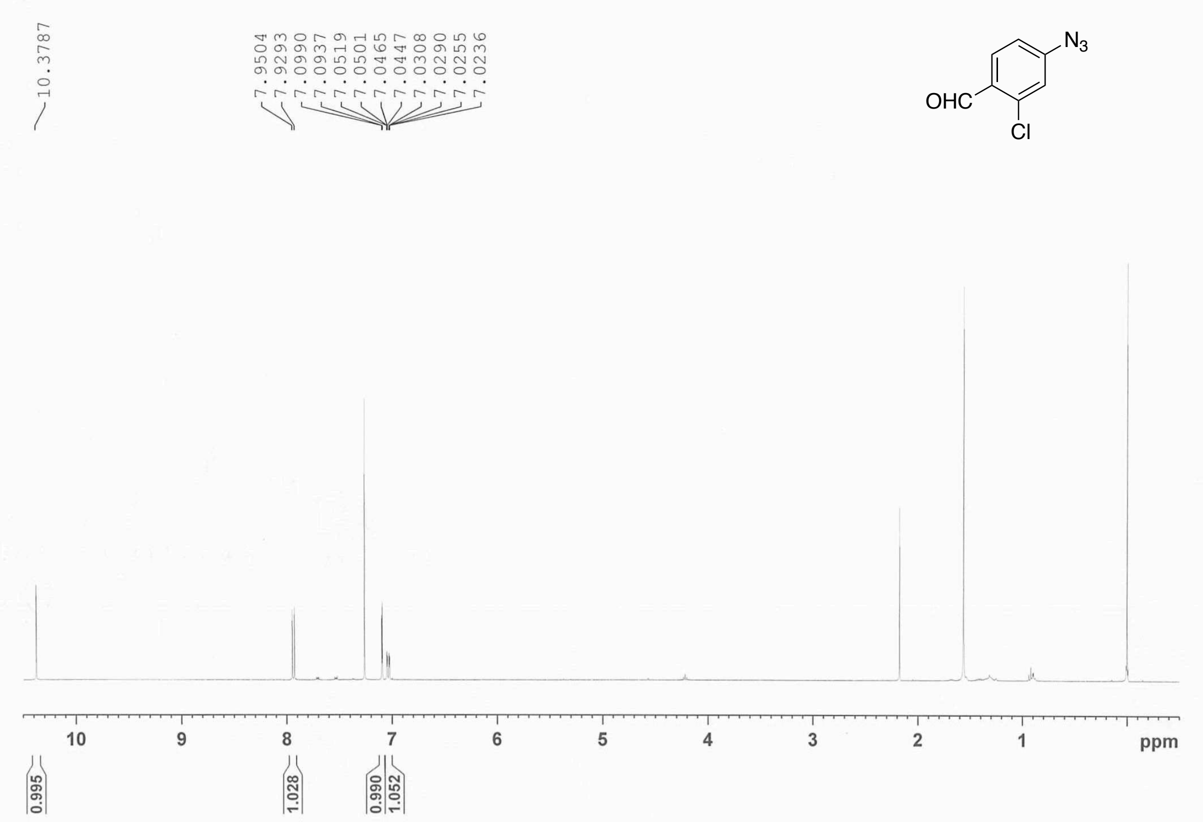

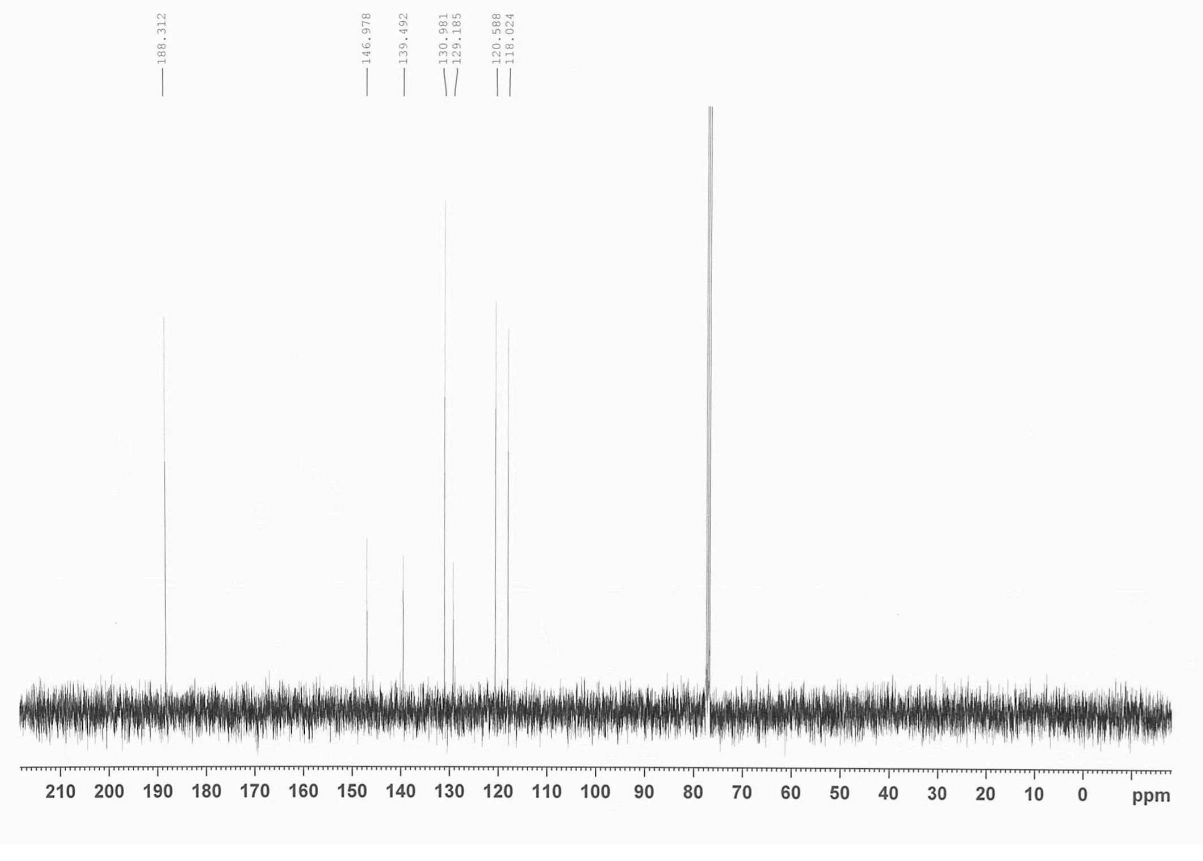

^{1}H NMR (400 MHz) and ^{13}C NMR (101 MHz) spectra of 4-((2-azidoethoxy)methyl)benzaldehyde (**7f**) ($CDCl_3$)

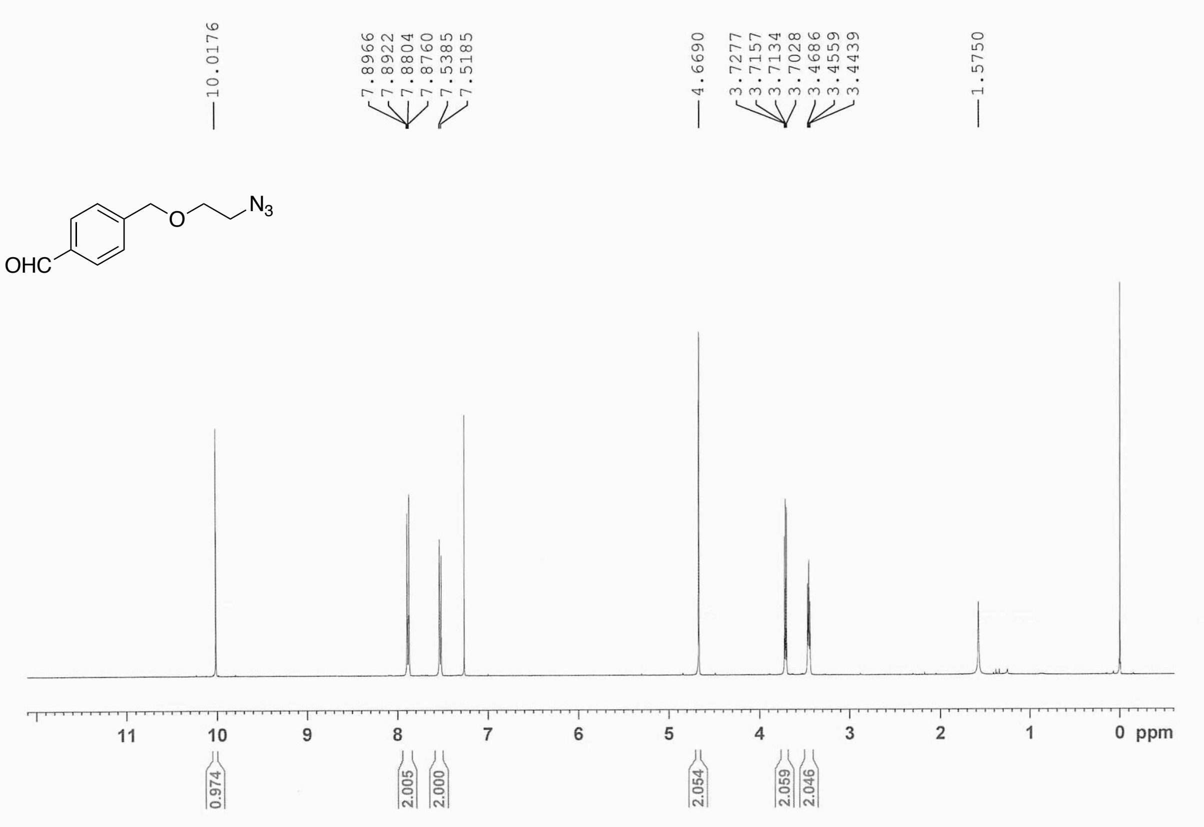

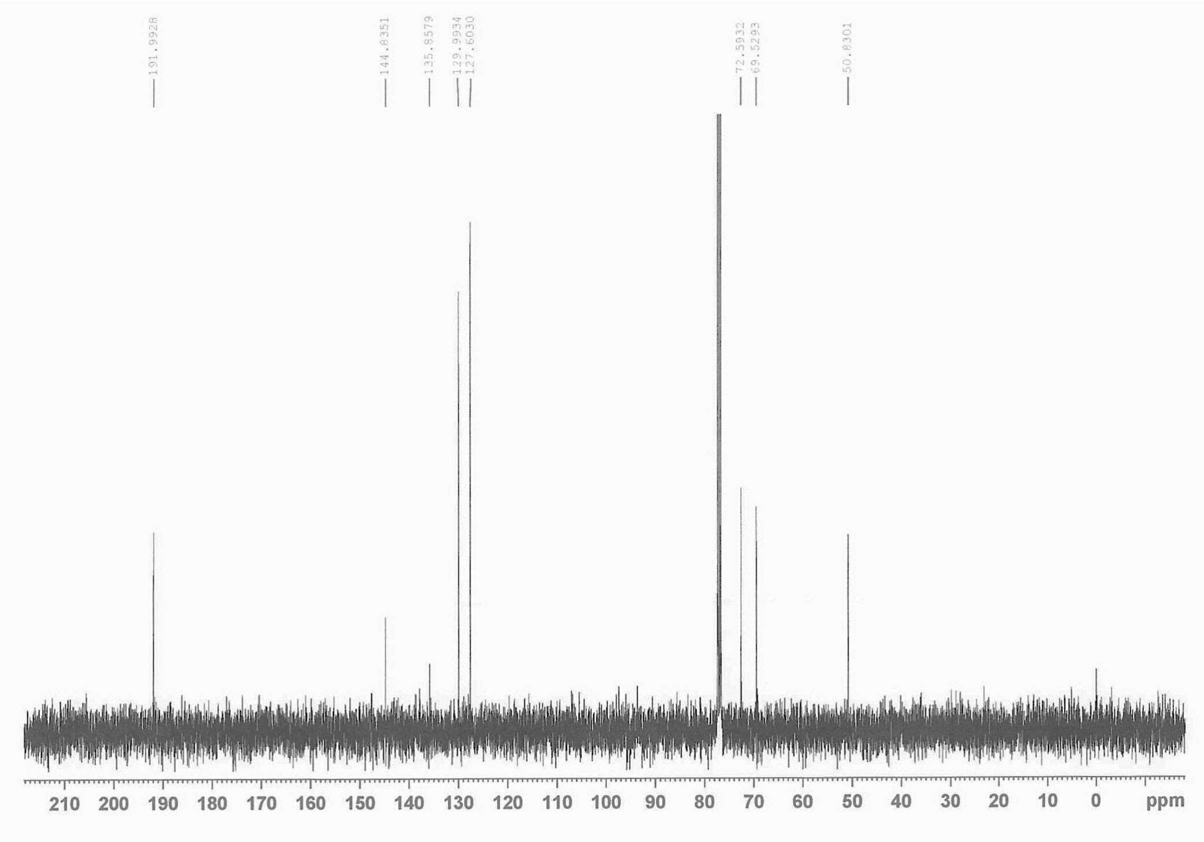

^{1}H NMR (400 MHz) and ^{13}C NMR (101 MHz) spectra of (4'-azido-[1,1'-biphenyl]-4-yl)(4-chlorophenyl)methanol (**8b**) ($CDCl_3$)

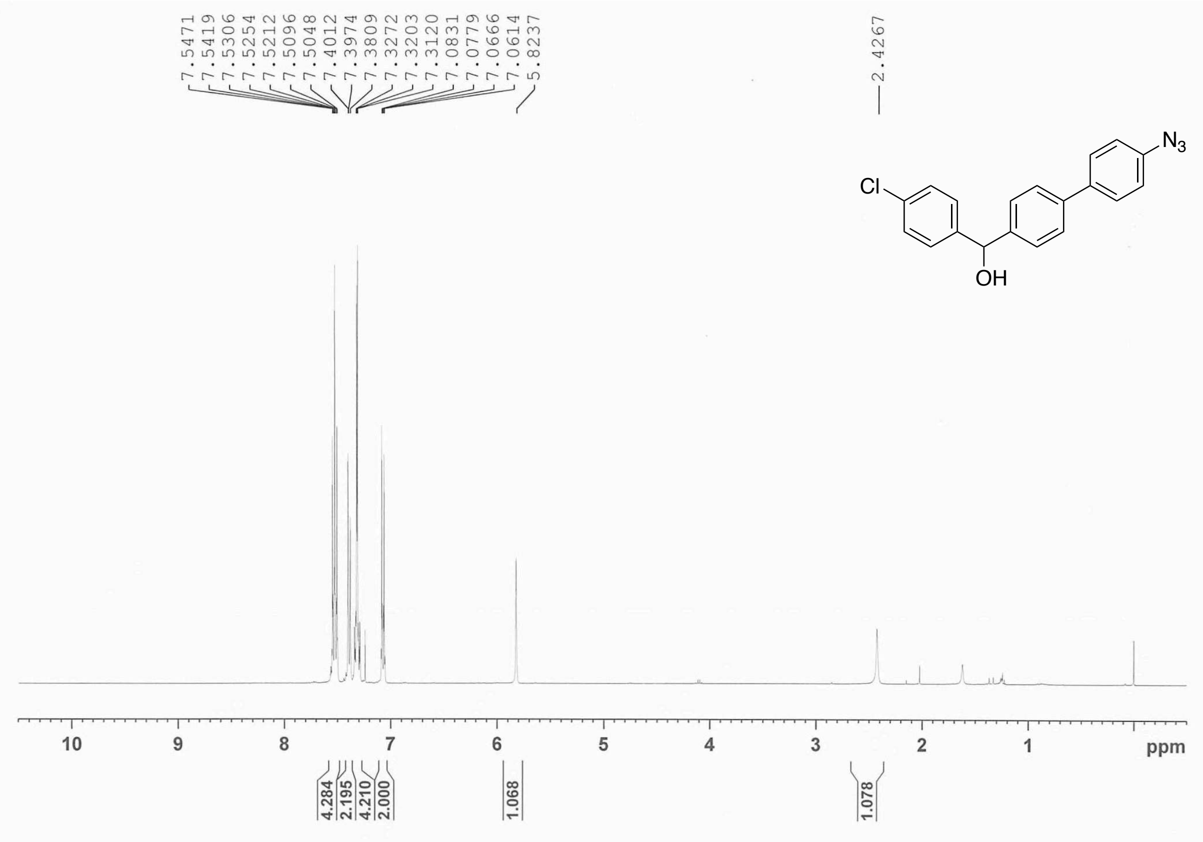

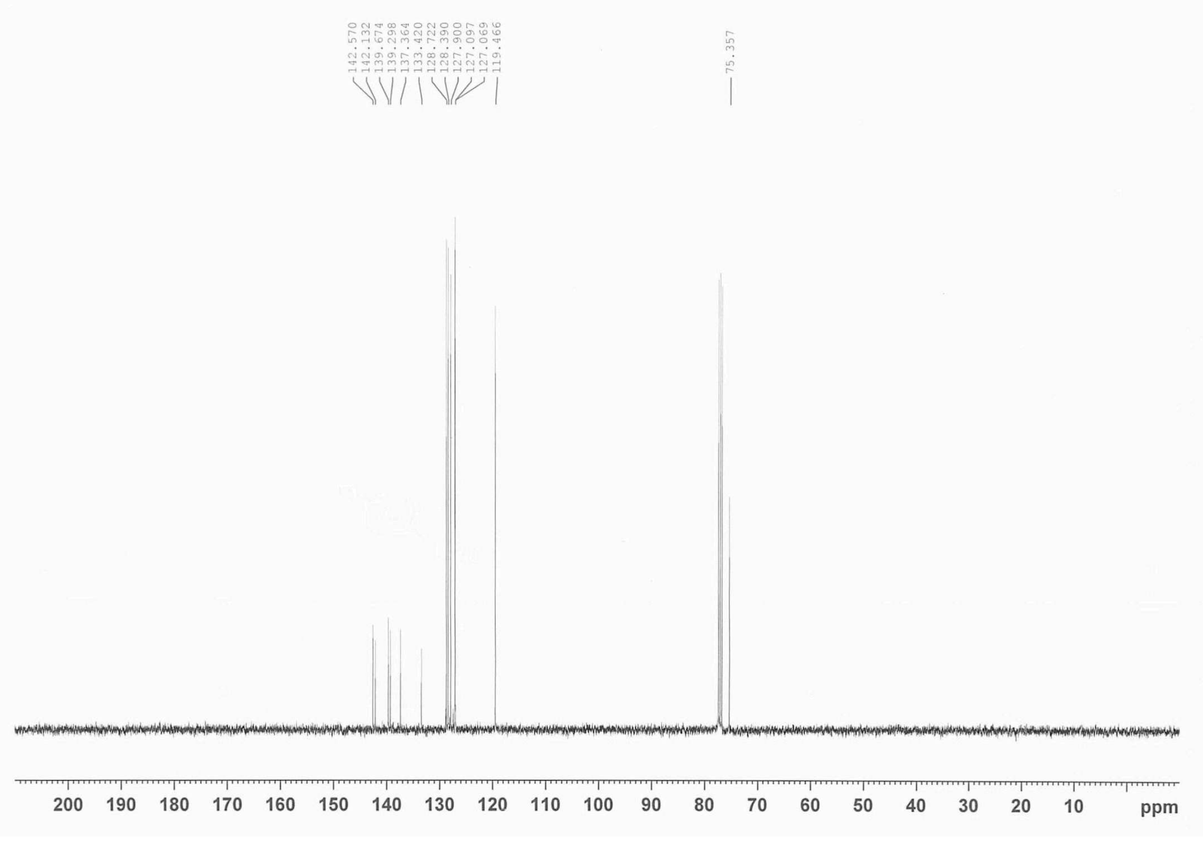

^{1}H NMR (400 MHz) and ^{13}C NMR (101 MHz) spectra of (4'-azido-[1,1'-biphenyl]-4-yl)(4-methoxyphenyl)methanol (**8c**) ($CDCl_3$)

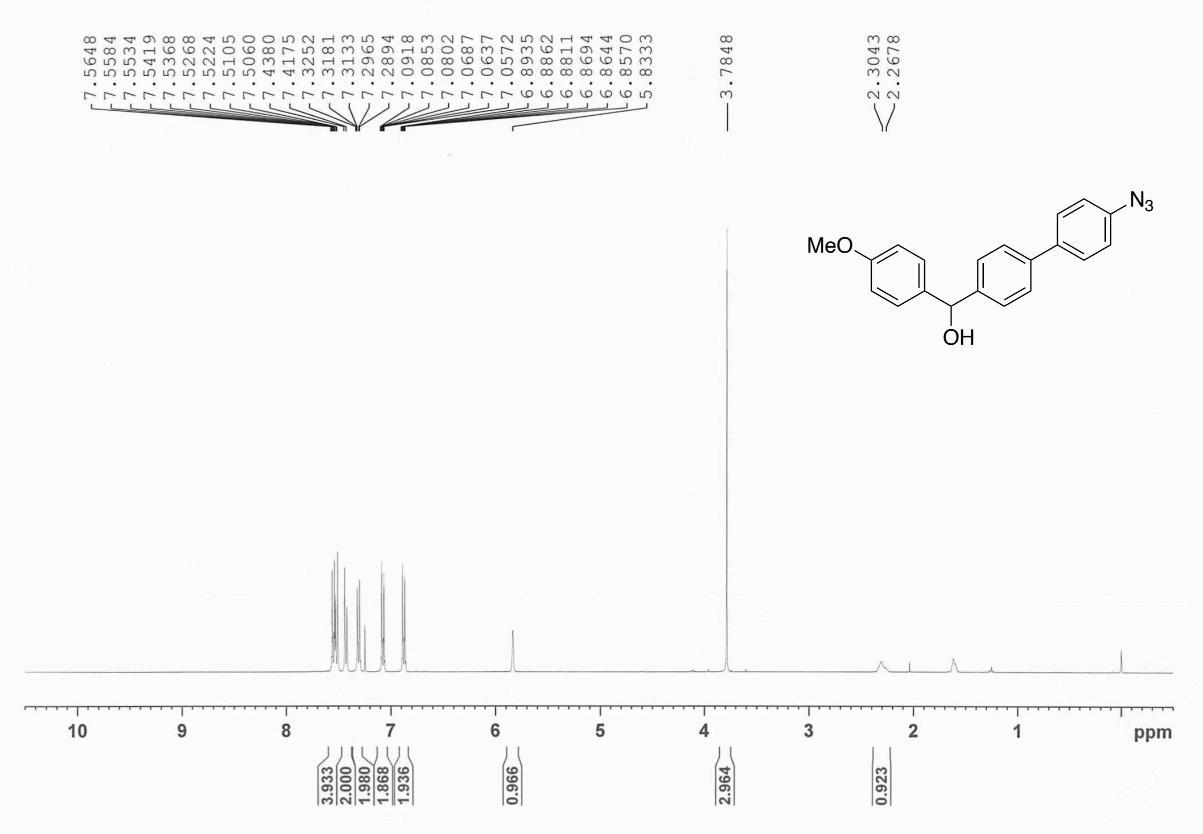

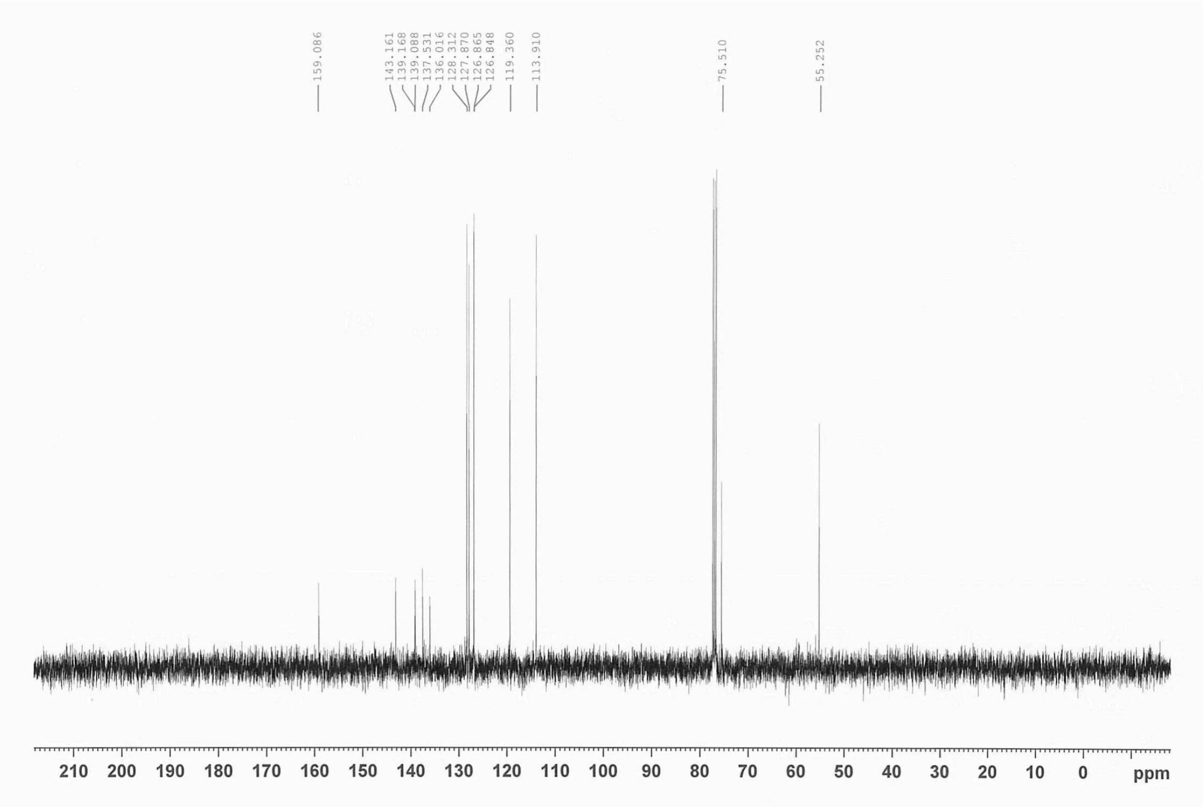

^{1}H NMR (400 MHz) and ^{13}C NMR (101 MHz) spectra of (4'-azido-[1,1'-biphenyl]-4-yl)(naphthalen-2-yl)methanol (**8d**) ($CDCl_3$)

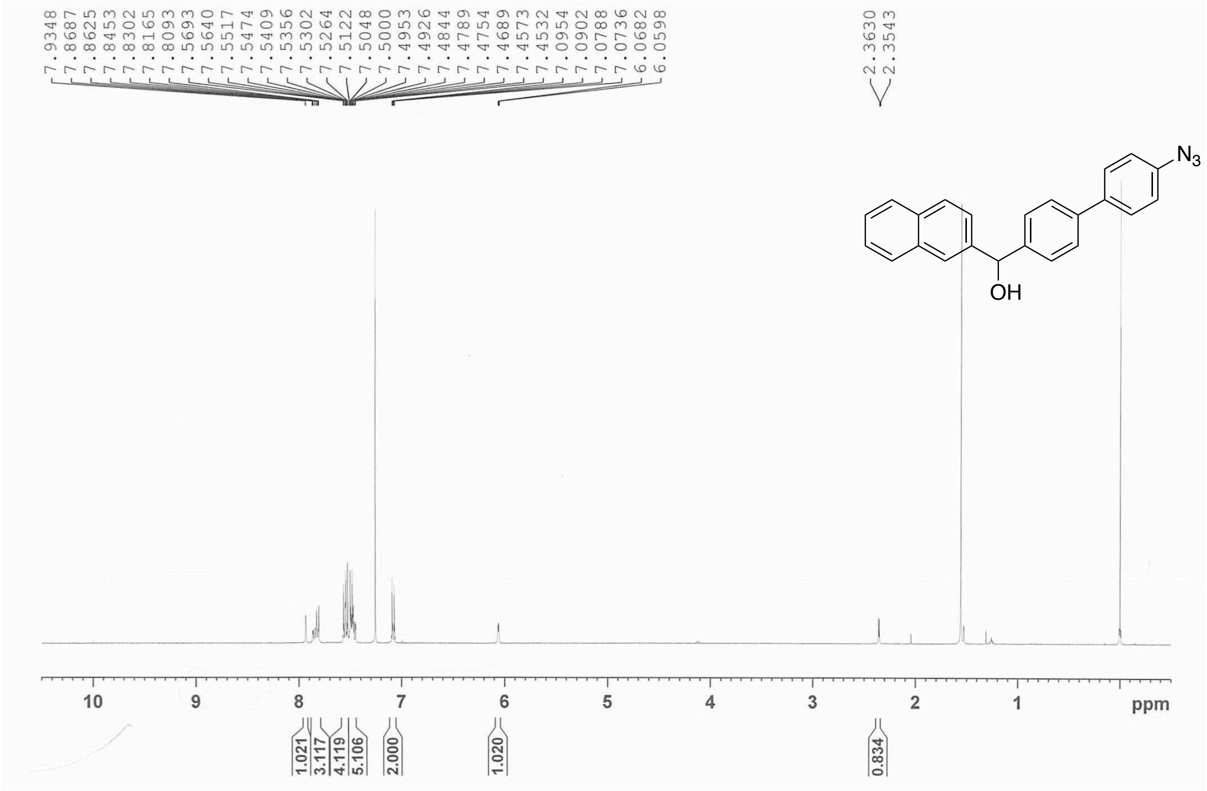

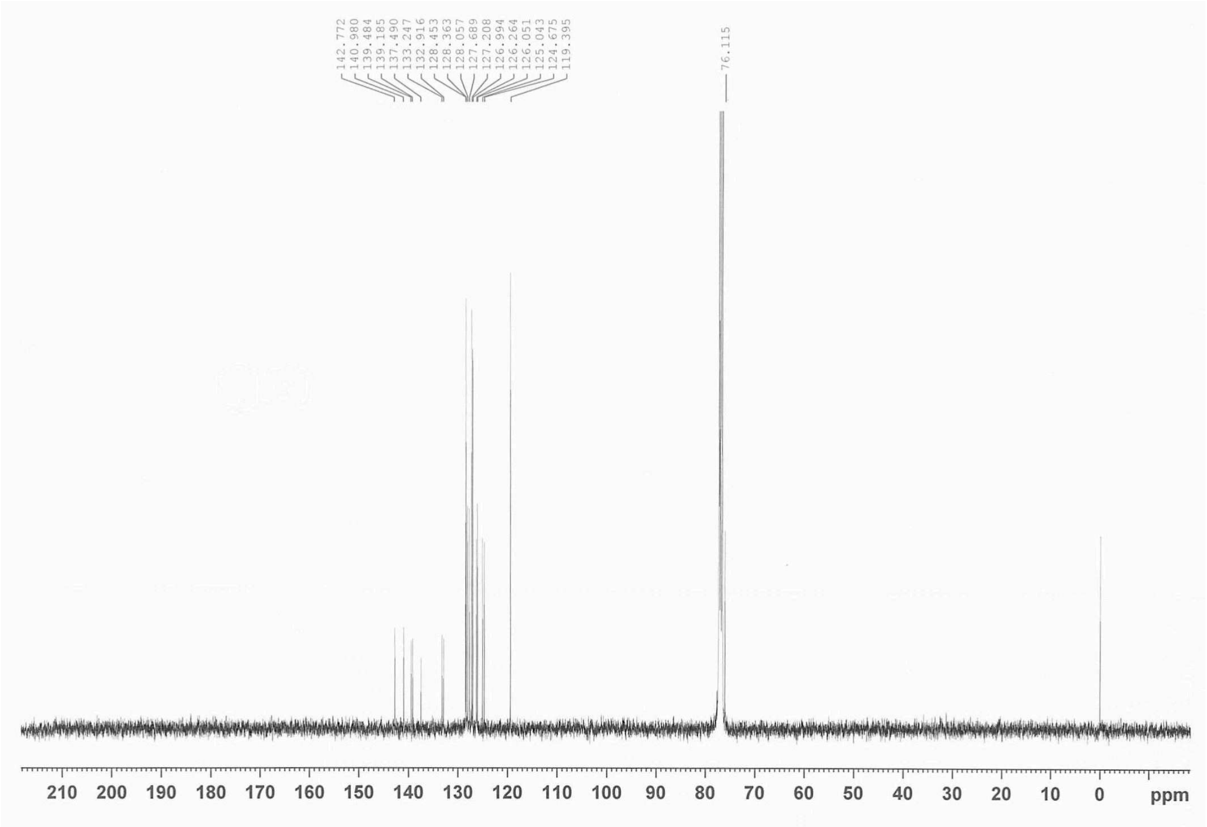

^{1}H NMR (400 MHz) and ^{13}C NMR (101 MHz) spectra of (4'-azido-[1,1'-biphenyl]-4-yl)(thiophen-2-yl)methanol (**8e**) ($CDCl_3$)

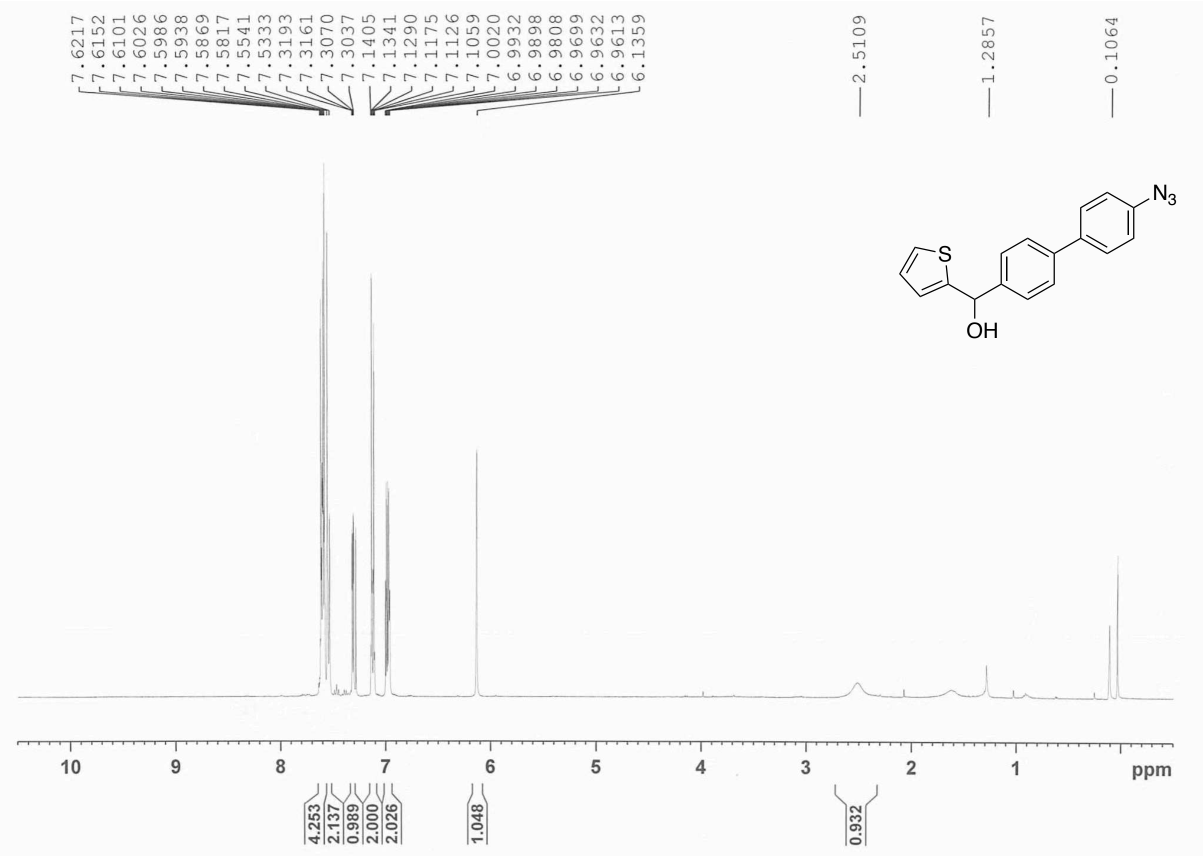

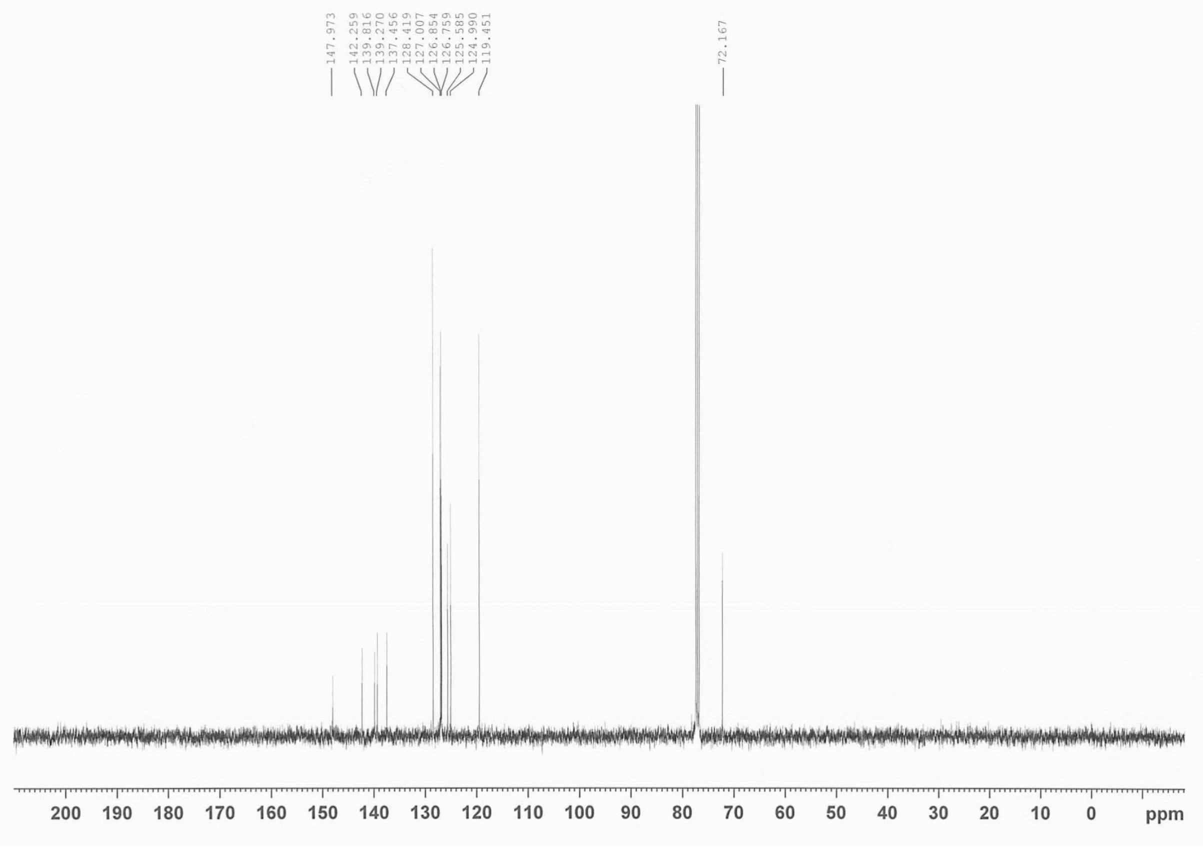

^{1}H NMR (400 MHz) and ^{13}C NMR (101 MHz) spectra of 2-(4'-Azido-[1,1'-biphenyl]-4-yl)propan-2-ol (**8f**) ($CDCl_3$)

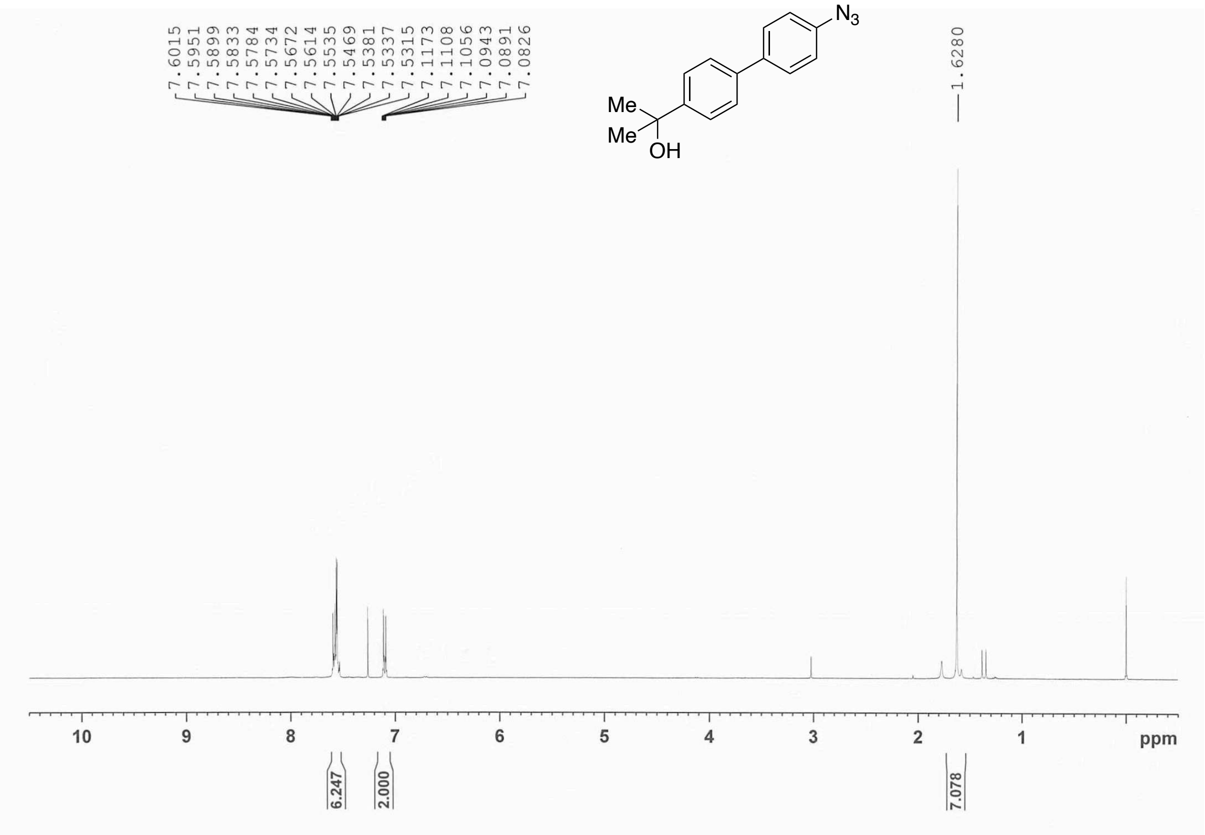

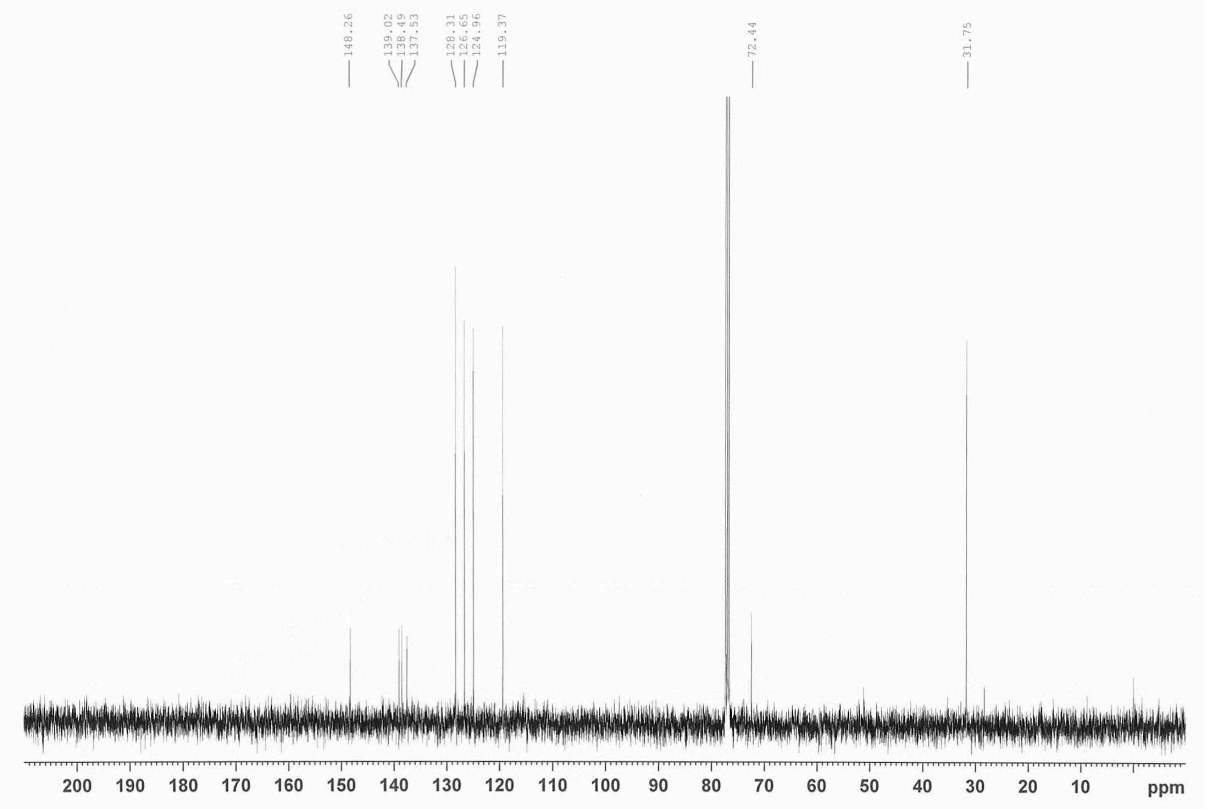

^{1}H NMR (400 MHz) and ^{13}C NMR (101 MHz) spectra of 1-(4'-azido-[1,1'-biphenyl]-4-yl)-2,2,2-trifluoro-1-phenylethan-1-ol (**8g**) ($CDCl_3$)

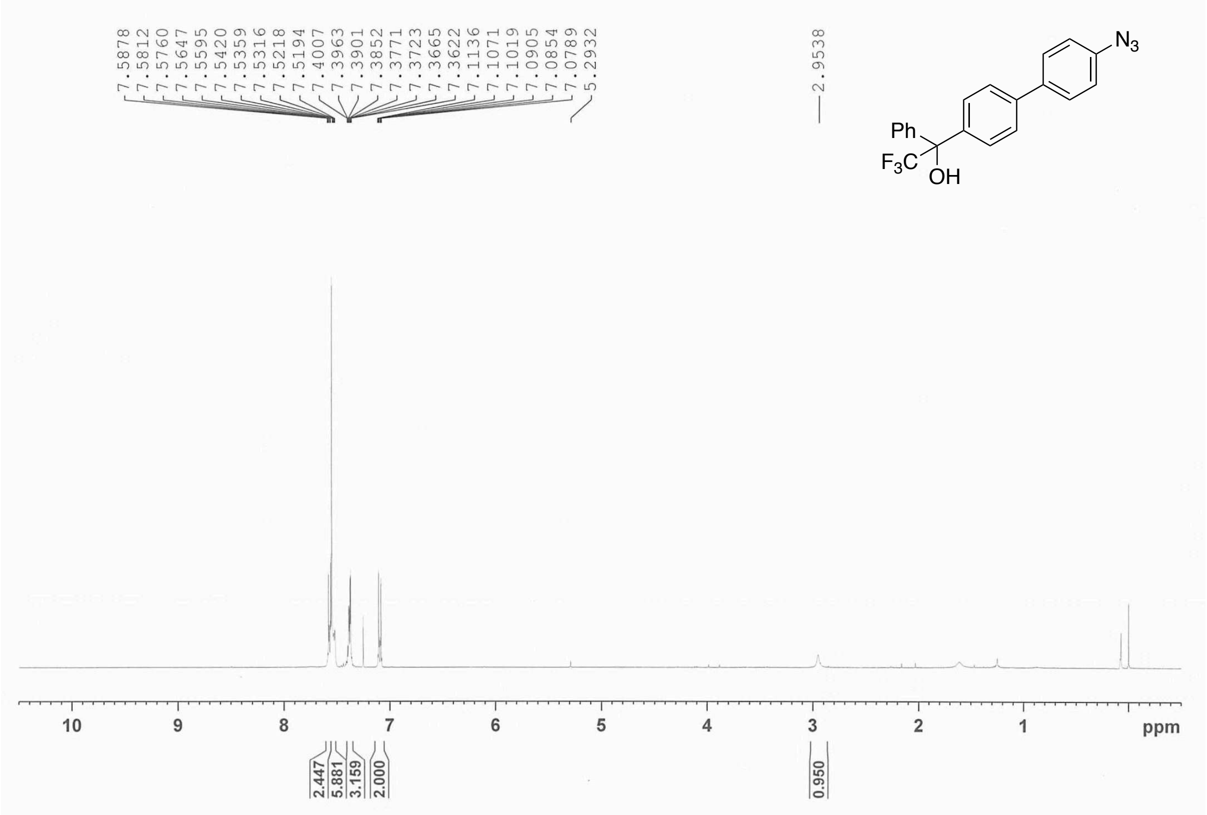

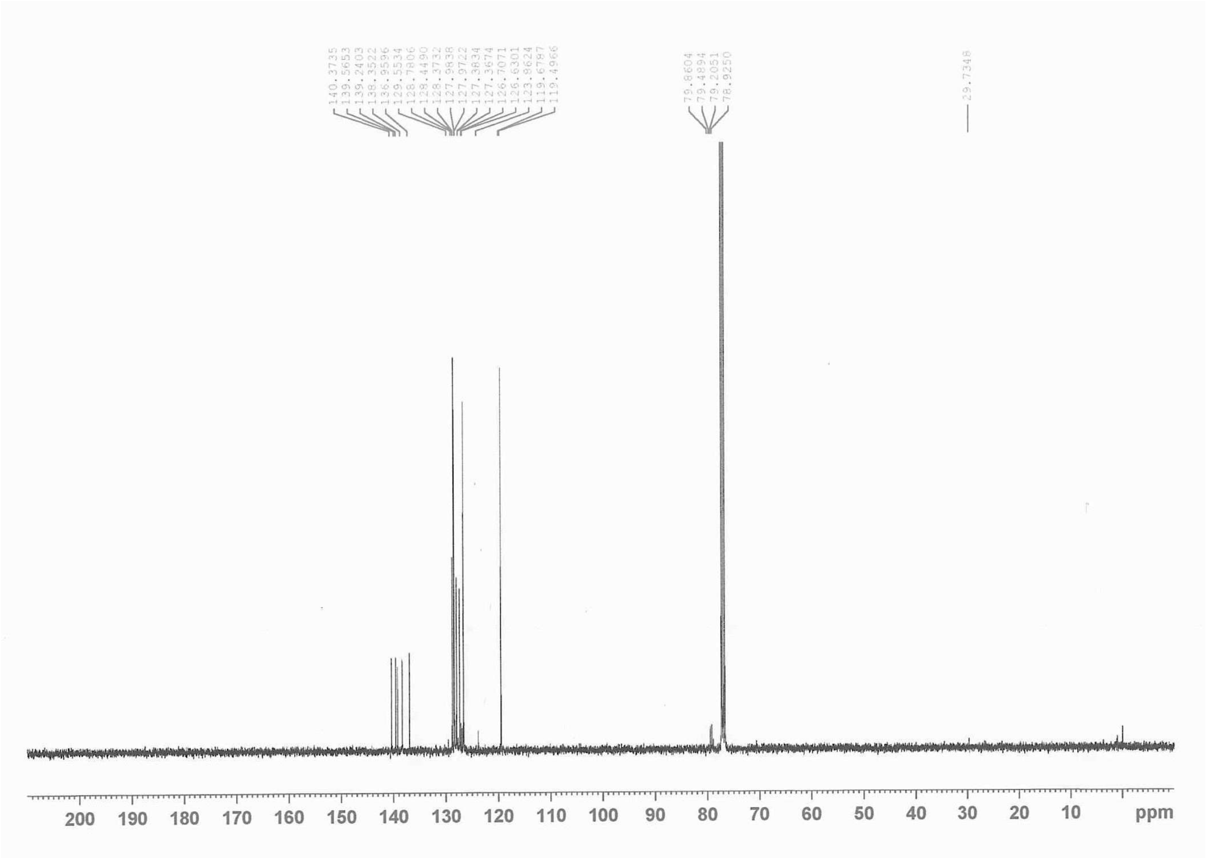

^{1}H NMR (400 MHz) and ^{13}C NMR (101 MHz) spectra of 4-allyl-4'-azido-1,1'-biphenyl (**8h**) ($CDCl_3$)

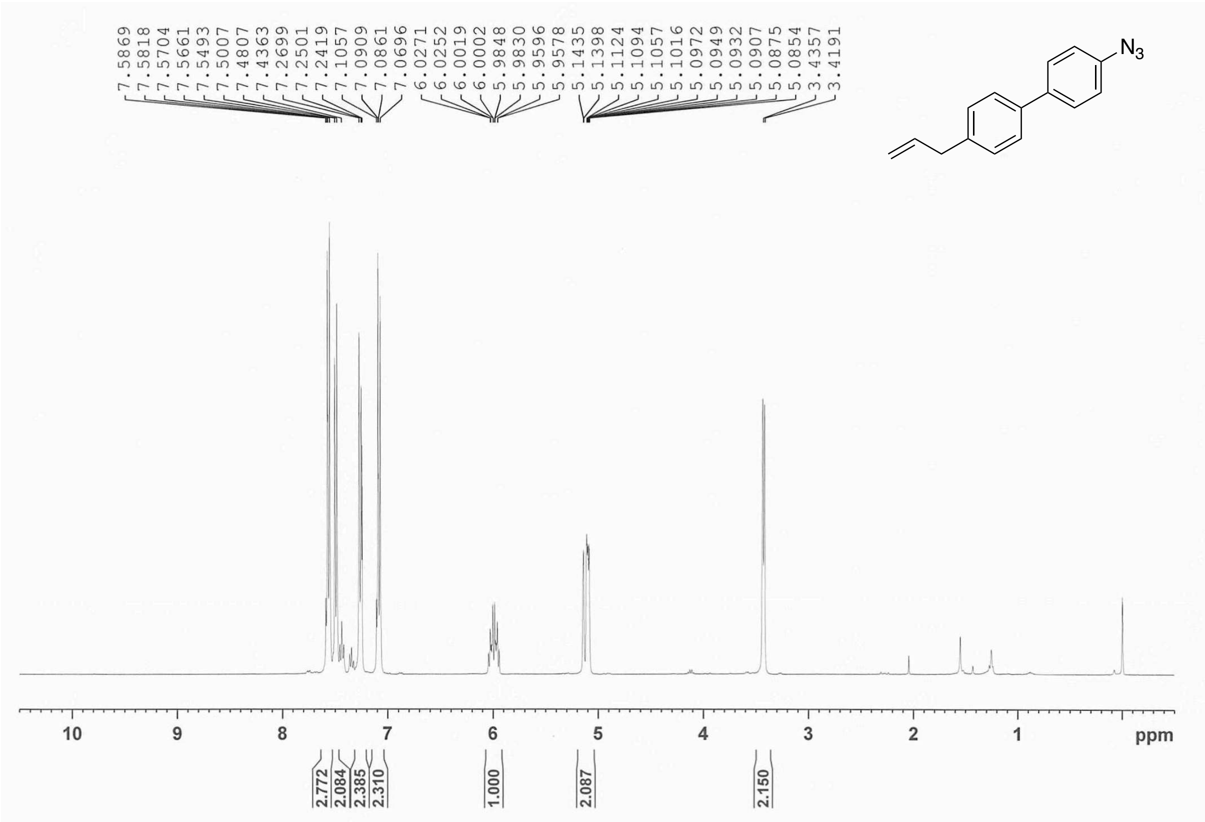

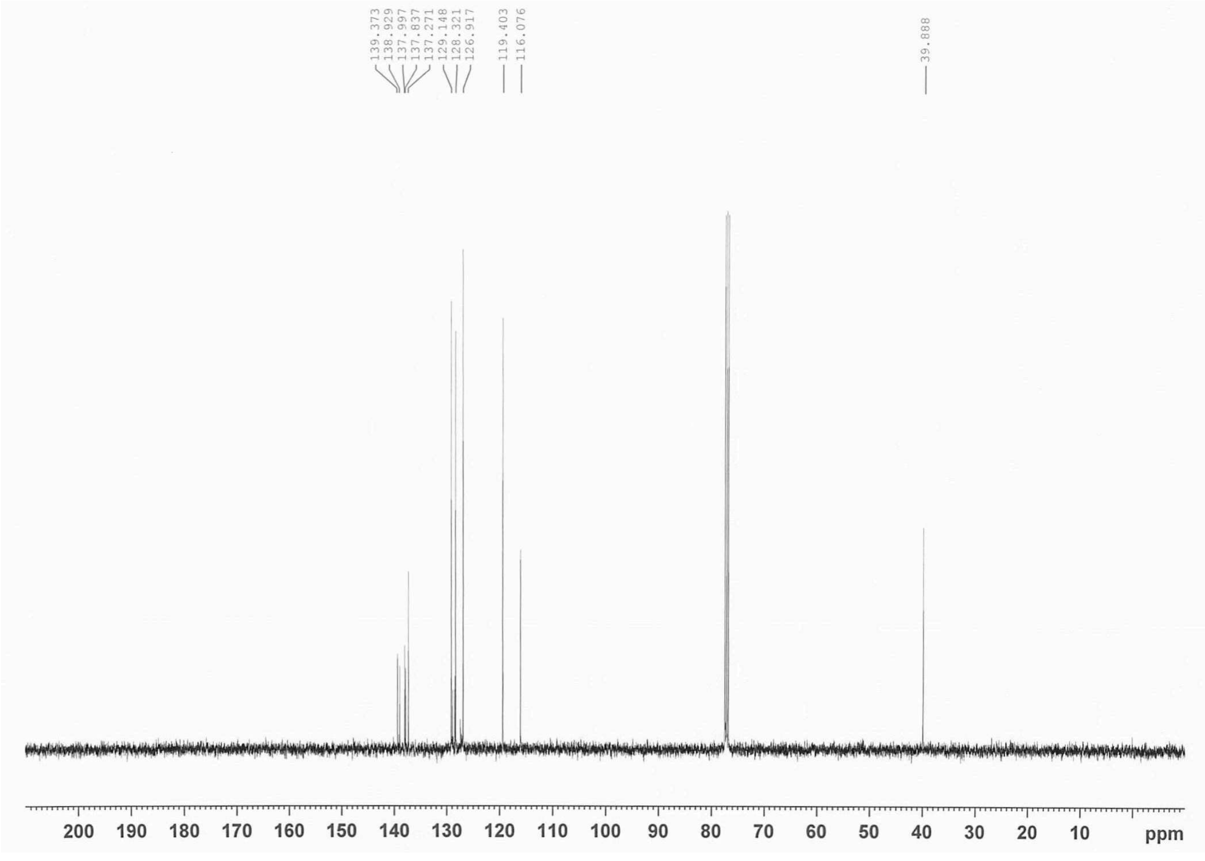

^{1}H NMR (400 MHz) and ^{13}C NMR (101 MHz) spectra of 4'-(4-(*p*-tolyl)-1*H*-1,2,3-triazol-1-yl)-[1,1'-biphenyl]-4-carbaldehyde (**16a**) ($CDCl_3$)

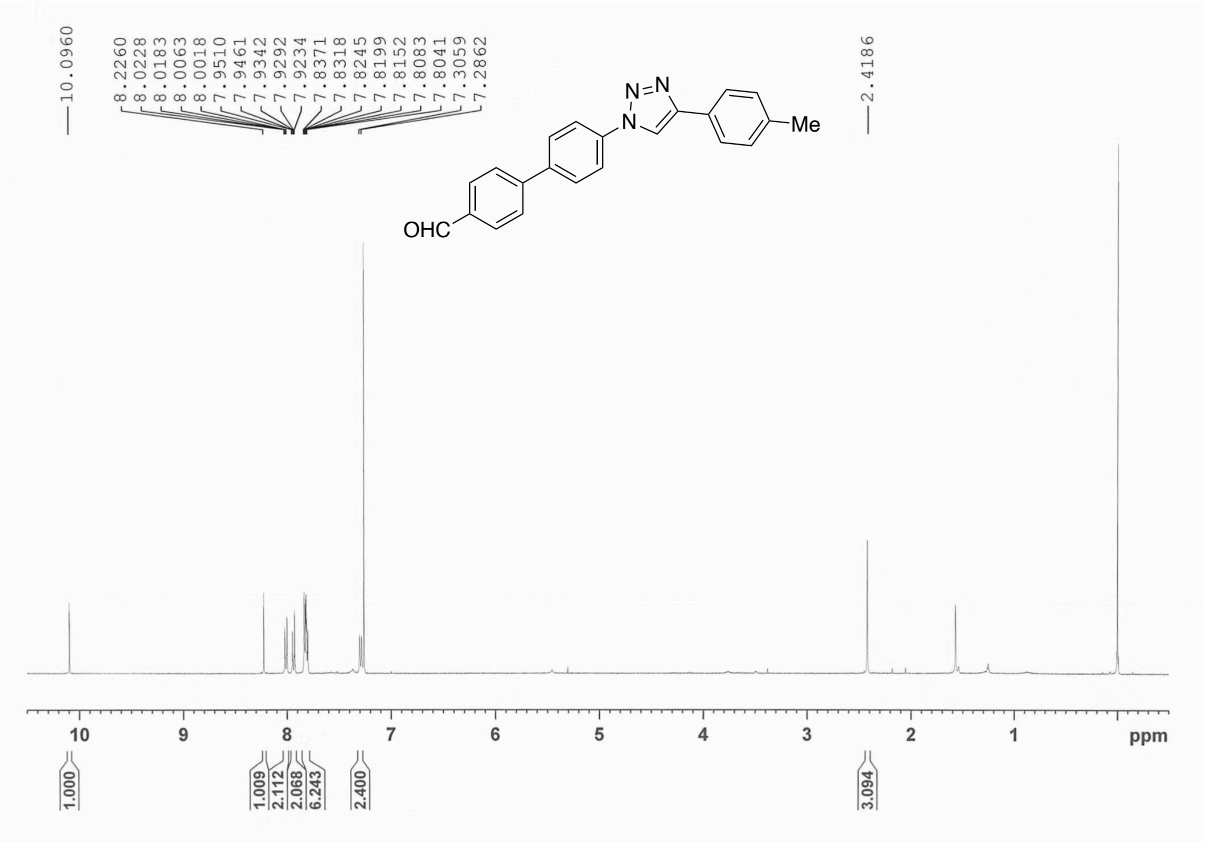

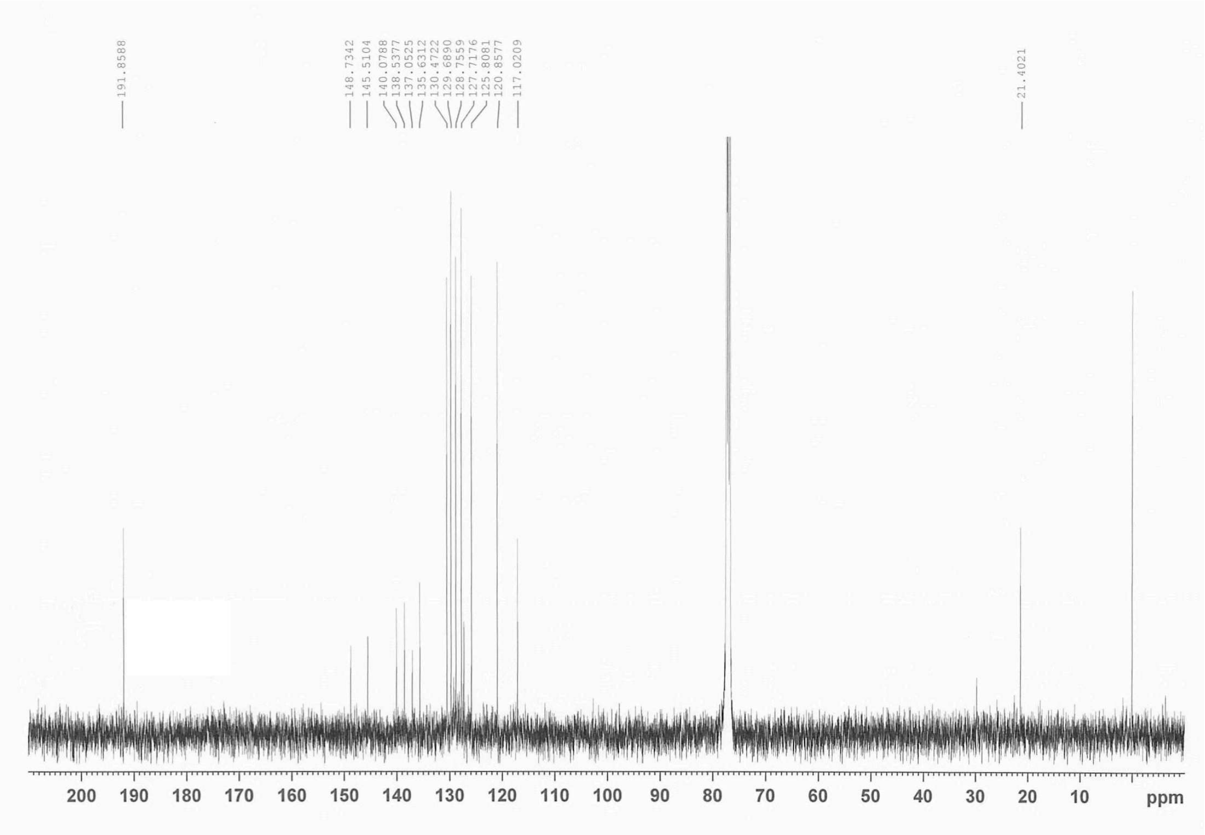

^{1}H NMR (400 MHz) and ^{13}C NMR (101 MHz) spectra of 4'-((5*aS**,6*S**,6*aR**)-6-(hydroxymethyl)-5,5a,6,6a,7,8-hexahydrocyclopropa[5,6]cycloocta[1,2-*d*][1,2,3]triazol-1(4*H*)-yl)-[1,1'-biphenyl]-4-carbaldehyde (**16b**) ($CDCl_3$)

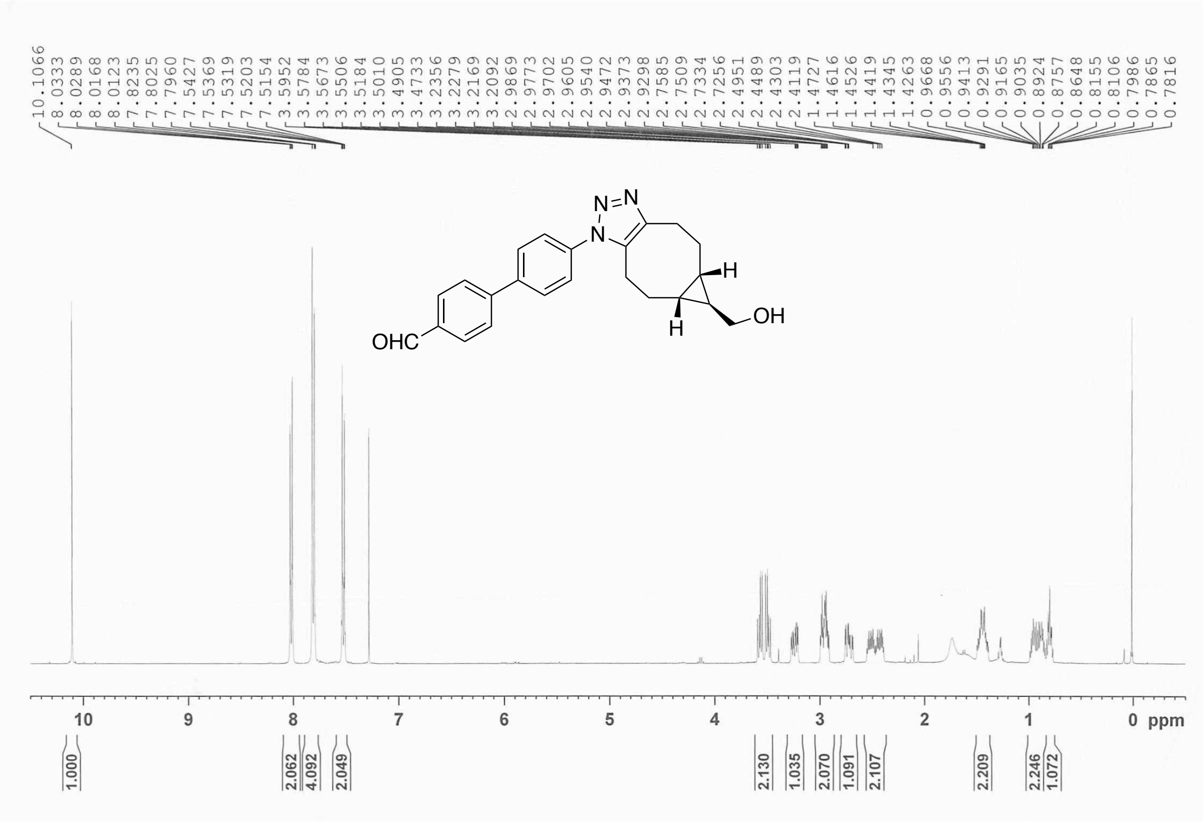

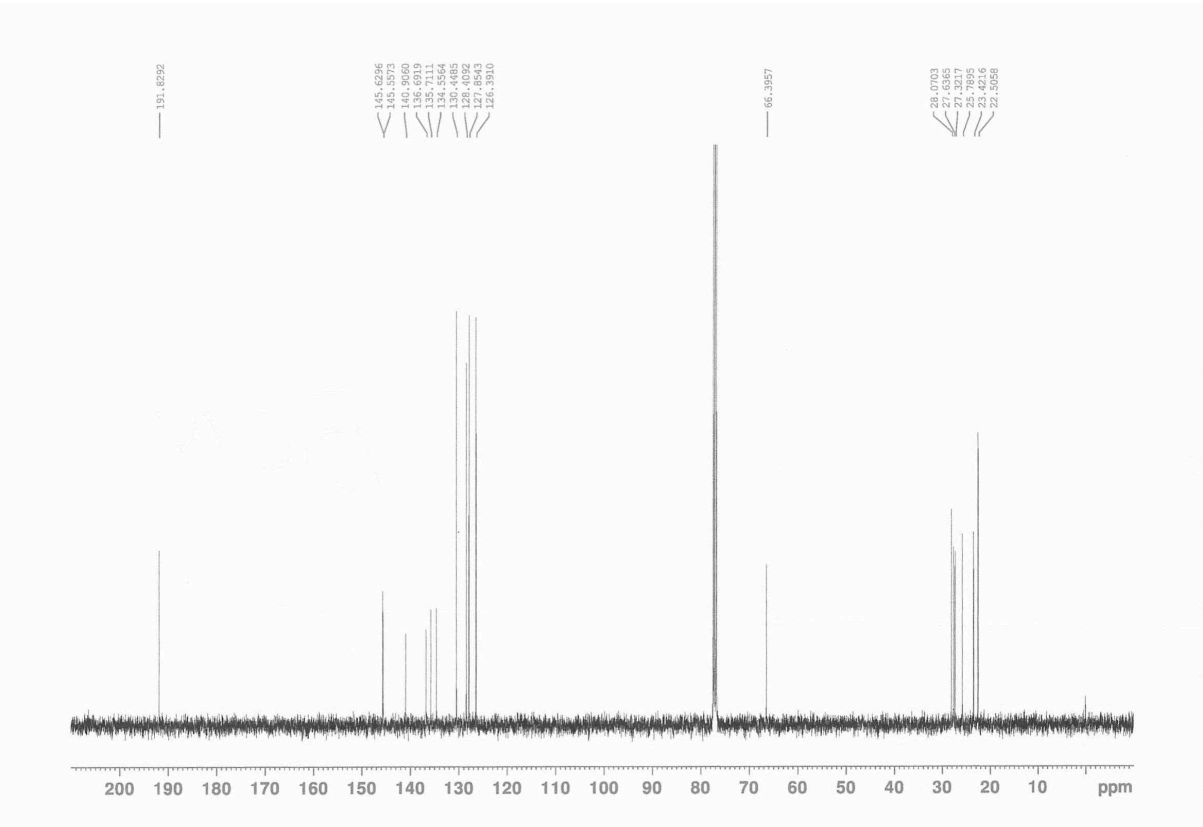

^{1}H NMR (400 MHz) and ^{13}C NMR (101 MHz) spectra of 4'-(1*H*-benzo[*d*][1,2,3]triazol-1-yl)-[1,1'-biphenyl]-4-carbaldehyde (**16c**) ($CDCl_3$)

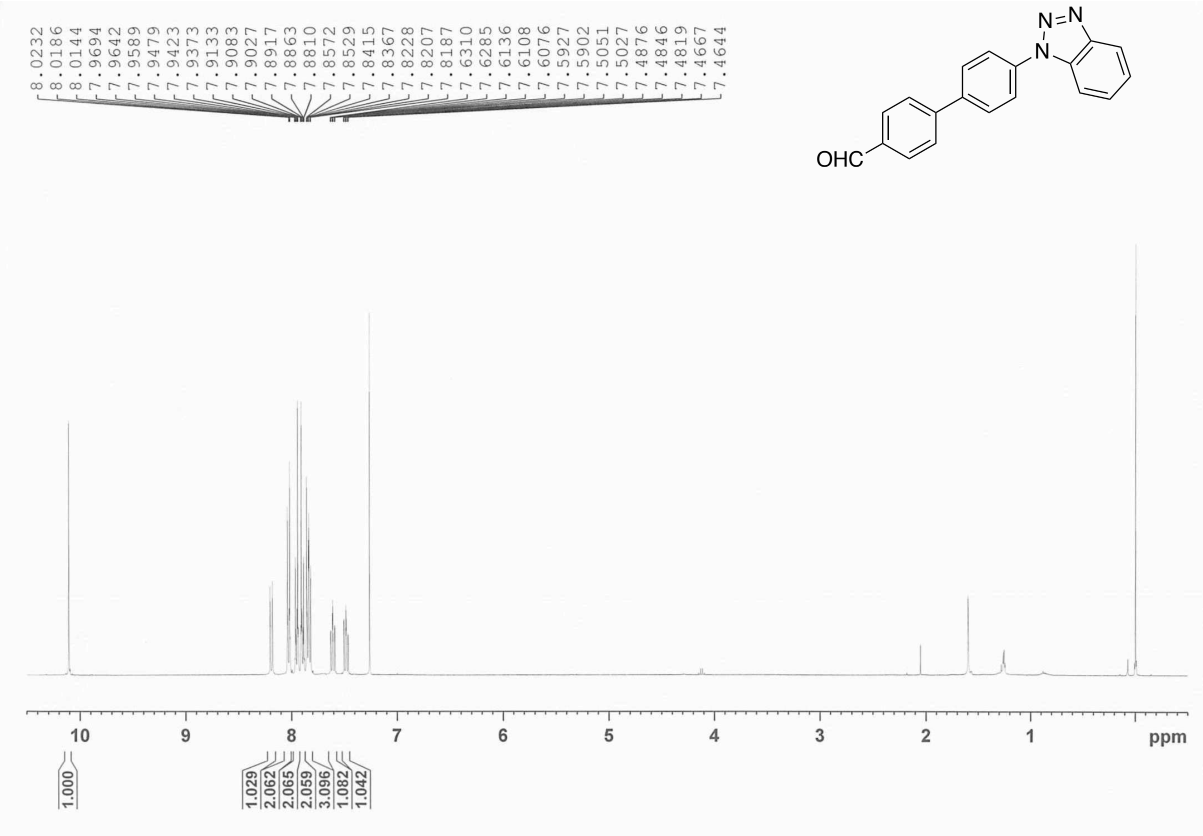

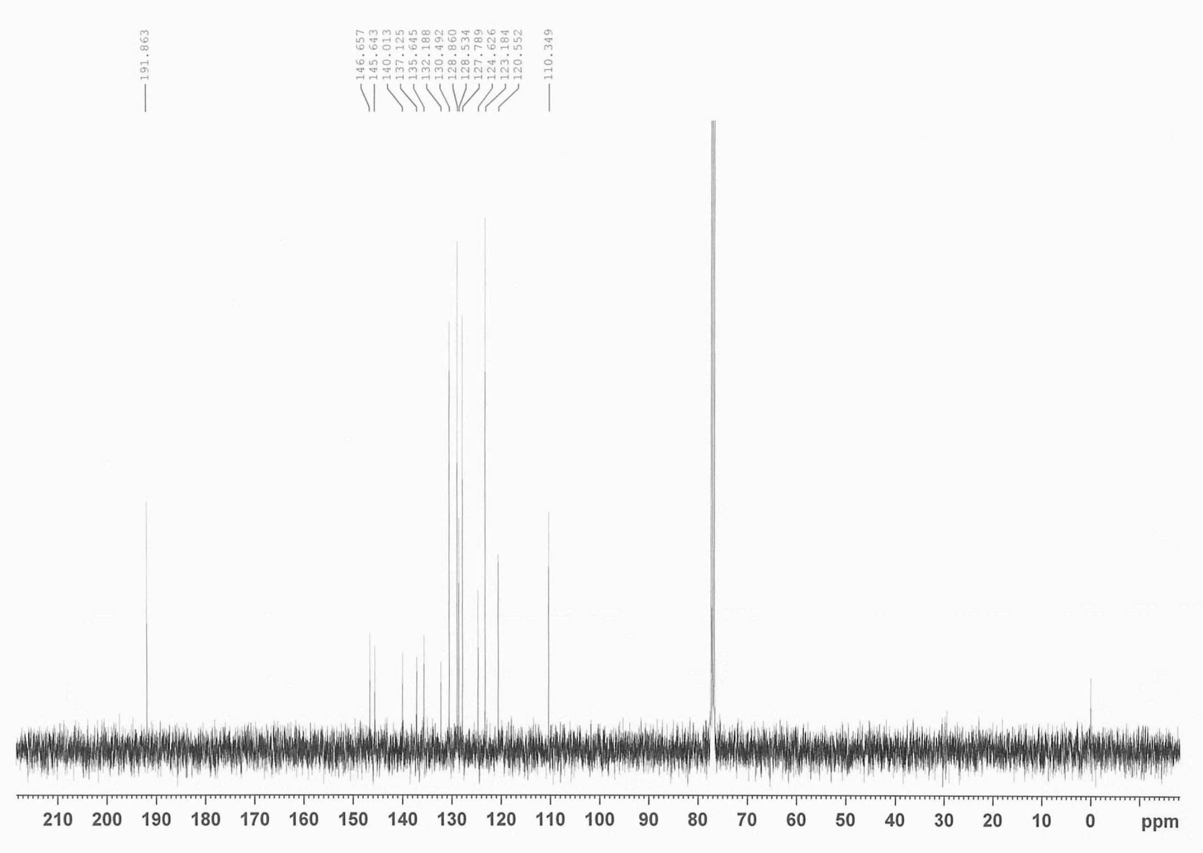

^{1}H NMR (400 MHz) and ^{13}C NMR (101 MHz) spectra of 1-(4'-azido-[1,1'-biphenyl]-4-yl)-*N*-methylmethanamine (**19**) ($CDCl_3$)

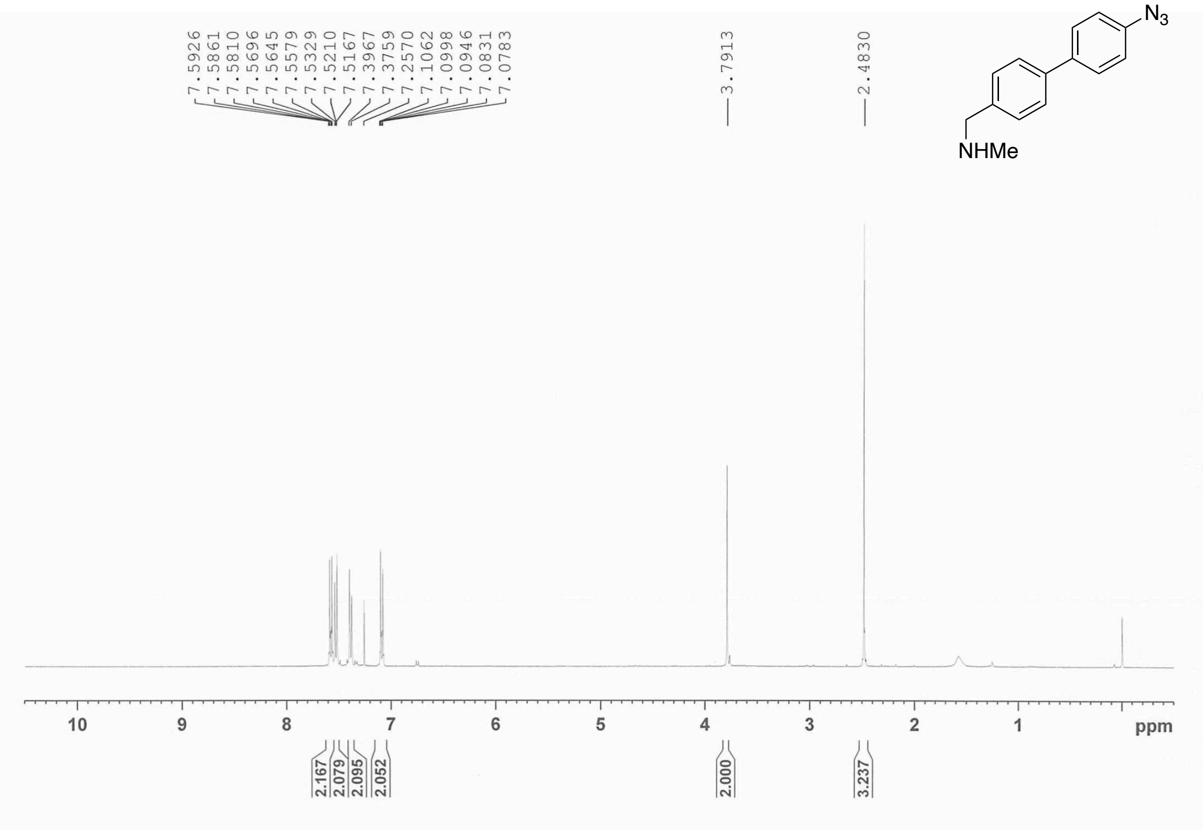

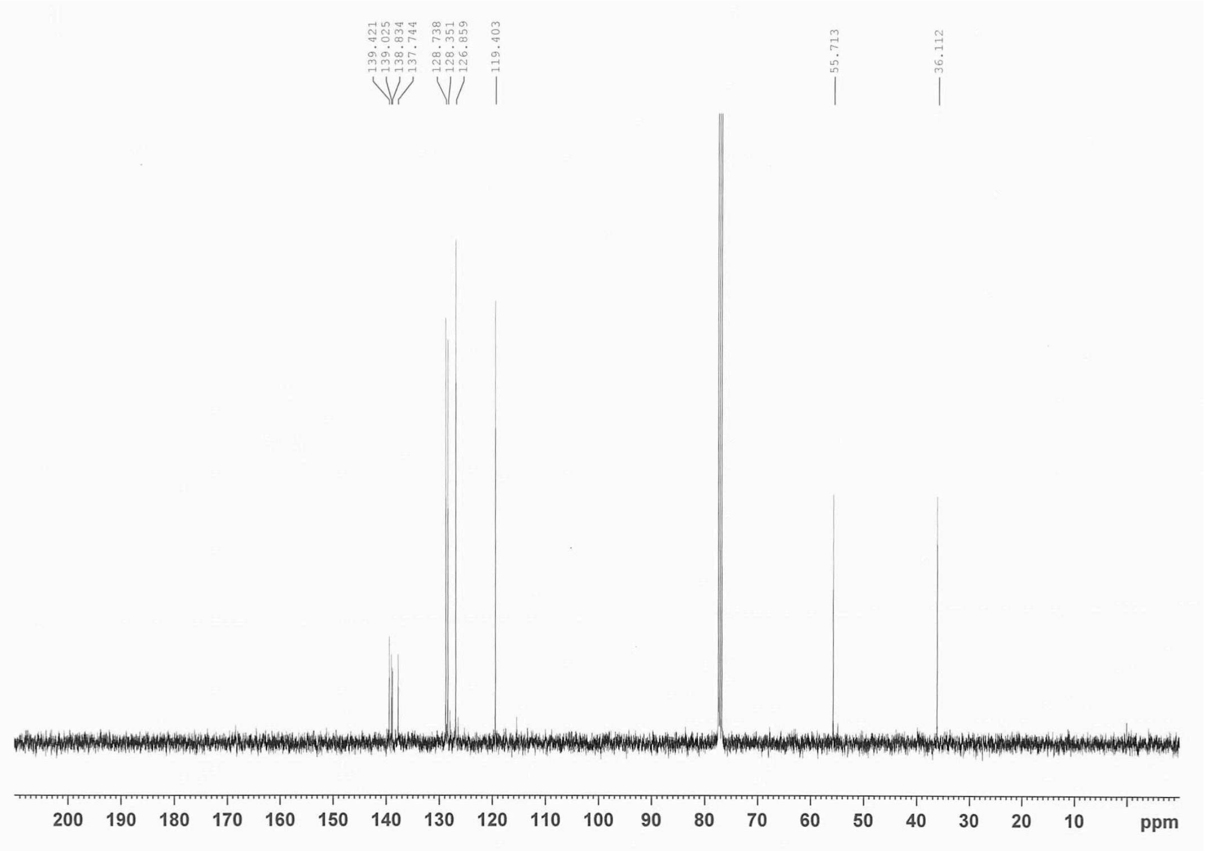

^{1}H NMR (400 MHz) and ^{13}C NMR (101 MHz) spectra of (4'-amino-[1,1'-biphenyl]-4-yl)(phenyl)methanol (**20a**) ($CDCl_3$)

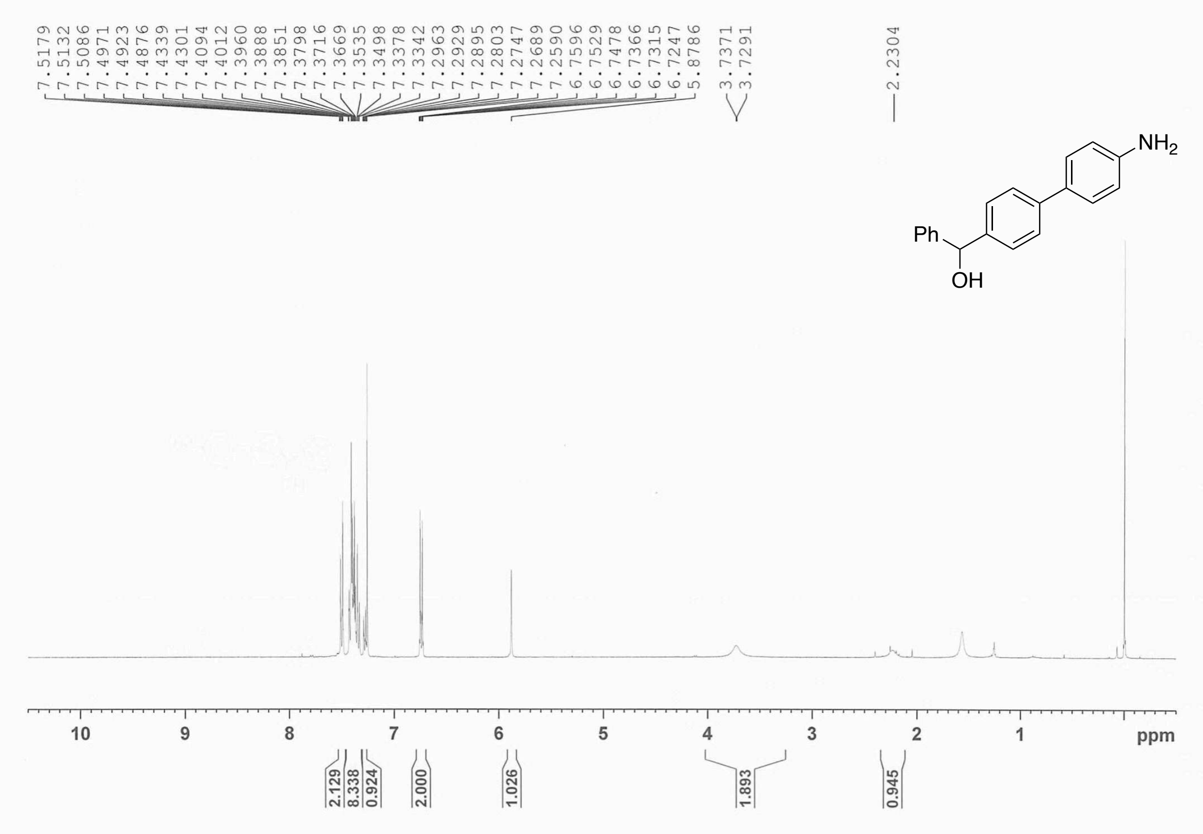

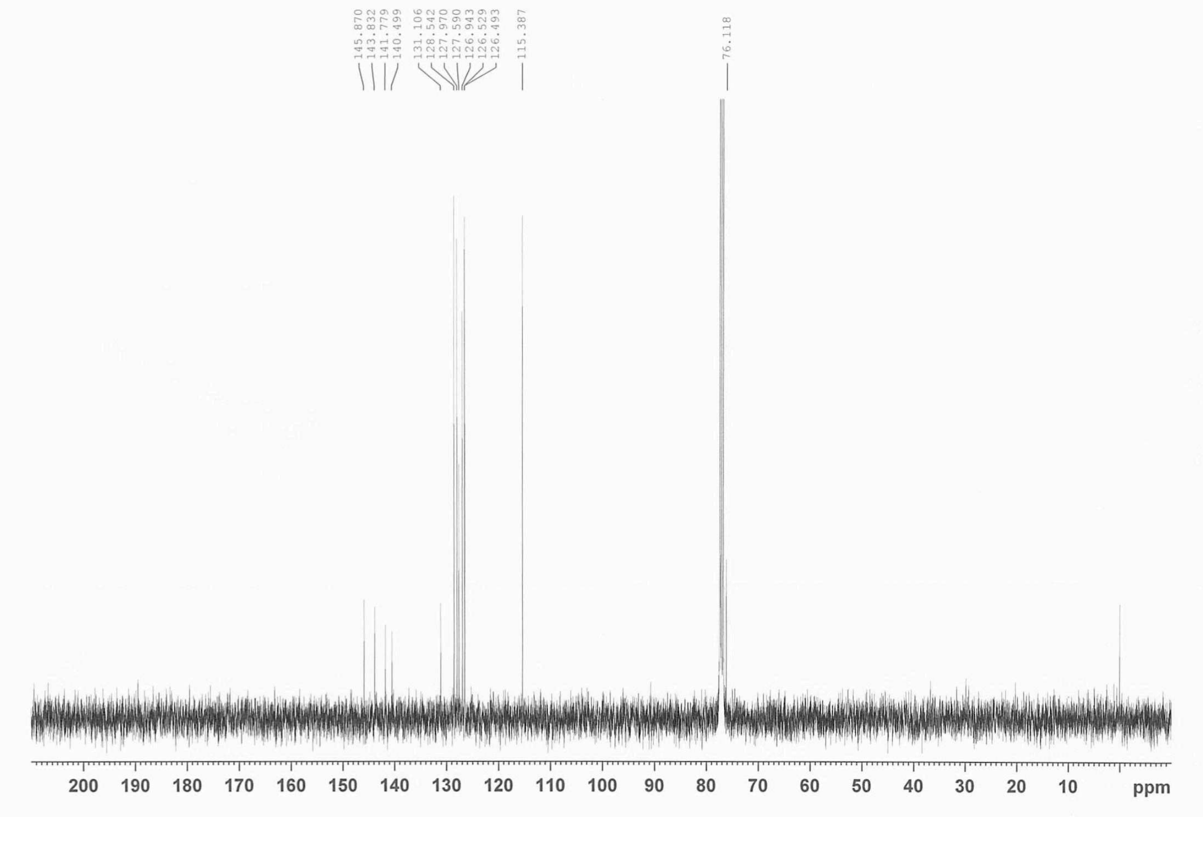

^{1}H NMR (400 MHz) and ^{13}C NMR (101 MHz) spectra of (4-azido-3-methylphenyl)(phenyl)methanol (**S1**) ($CDCl_3$)

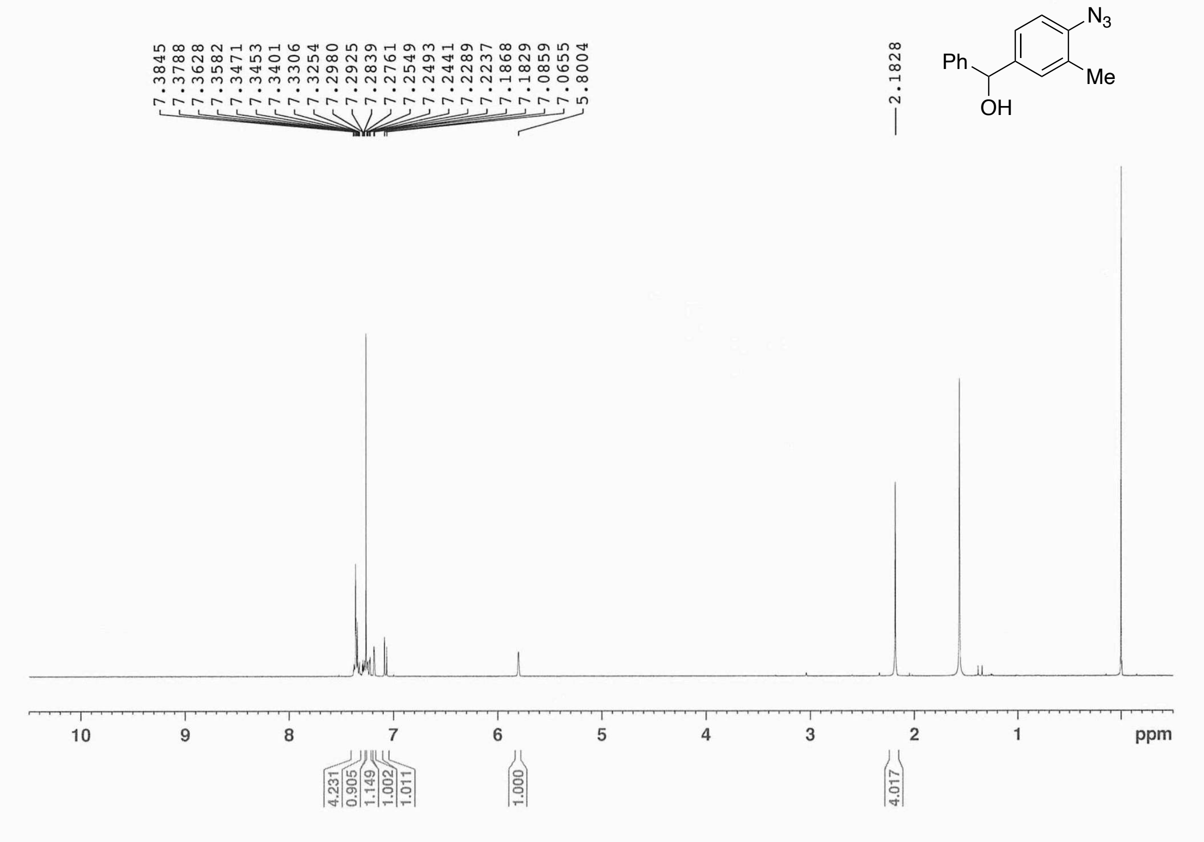

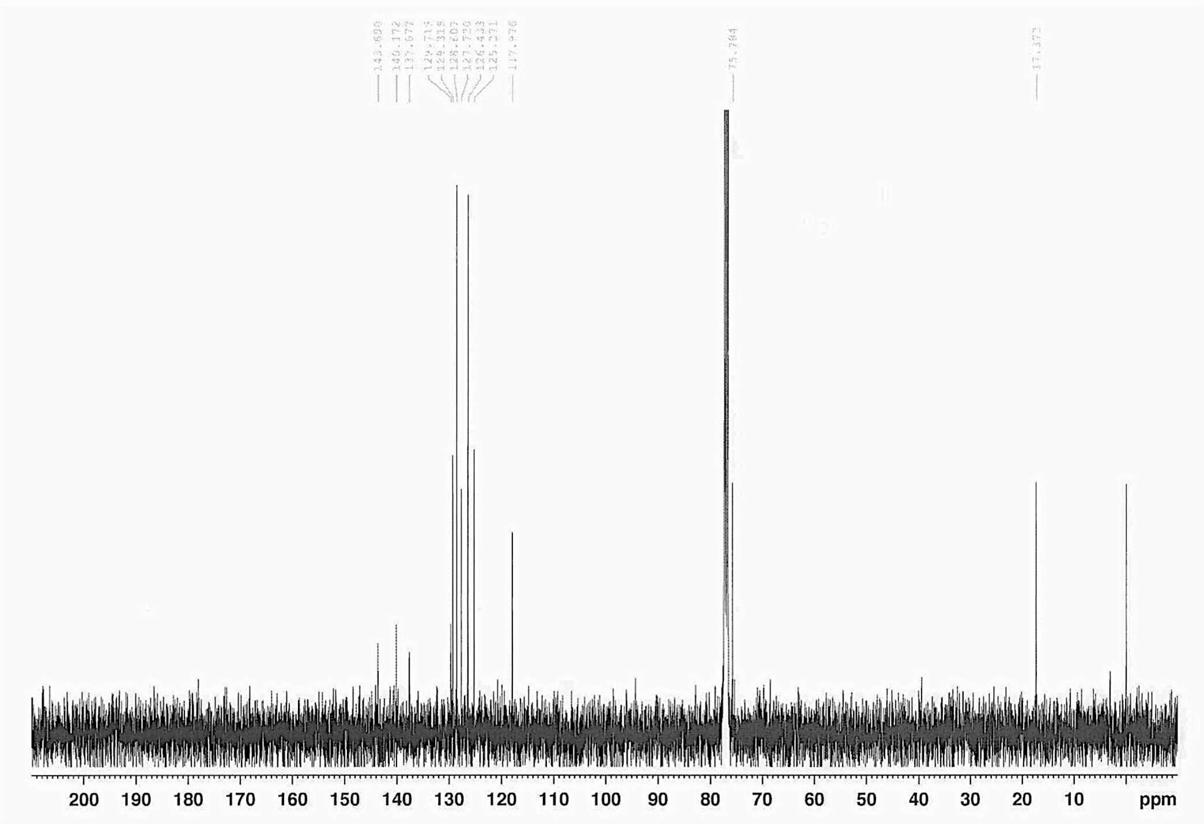

^{1}H NMR (400 MHz) and ^{13}C NMR (101 MHz) spectra of (4-amino-3-methylphenyl)(phenyl)methanol (**20c**) ($CDCl_3$)

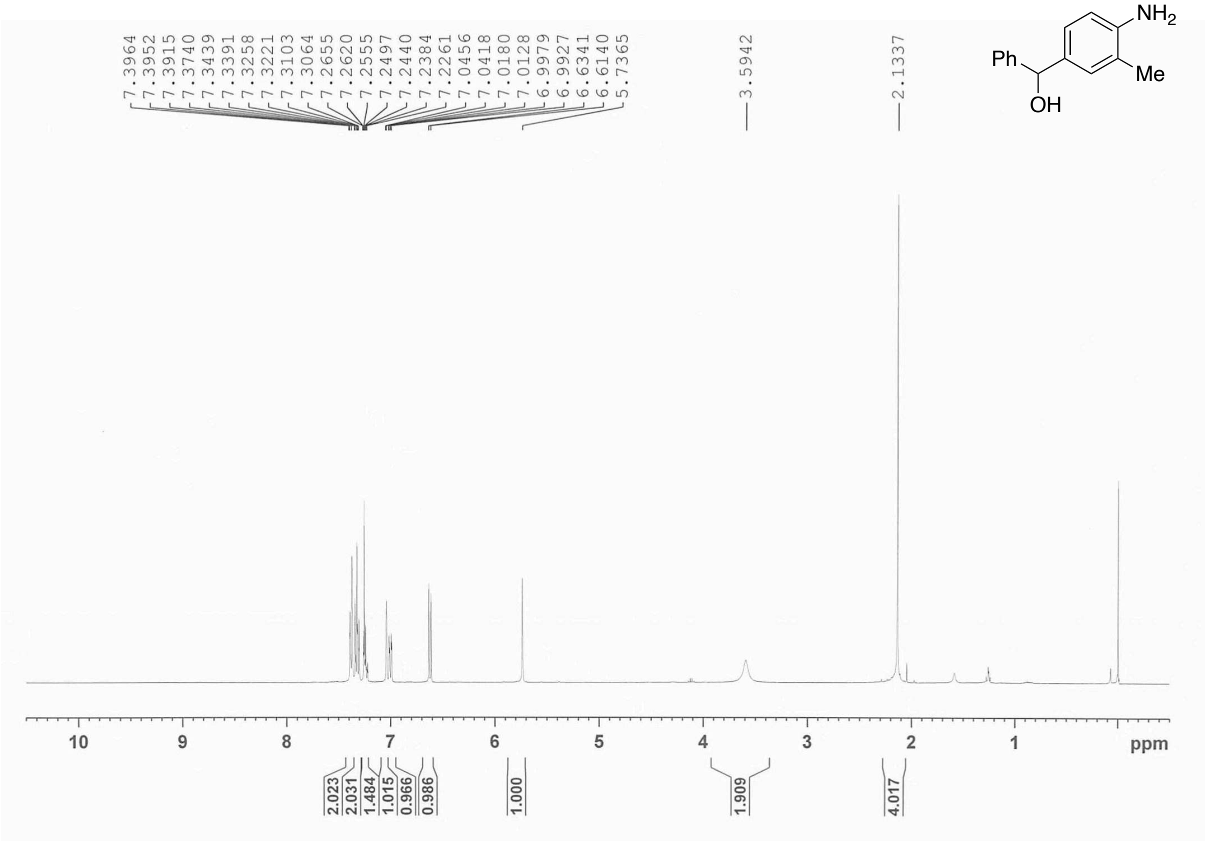

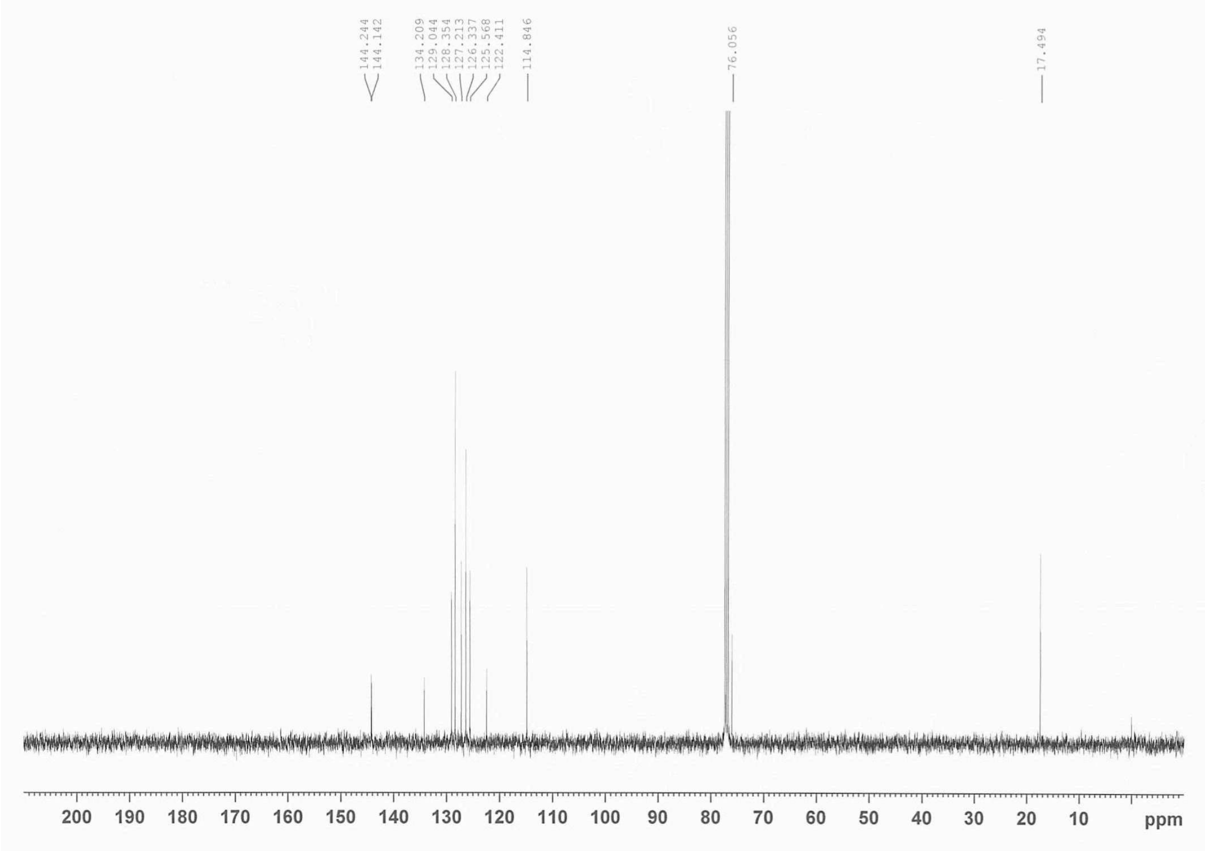

^1H NMR (400 MHz) and ^{13}C NMR (101 MHz) spectra of (3-azido-5-methoxyphenyl)(thiophen-2-yl)methanol (**S2**) ($CDCl_3$)

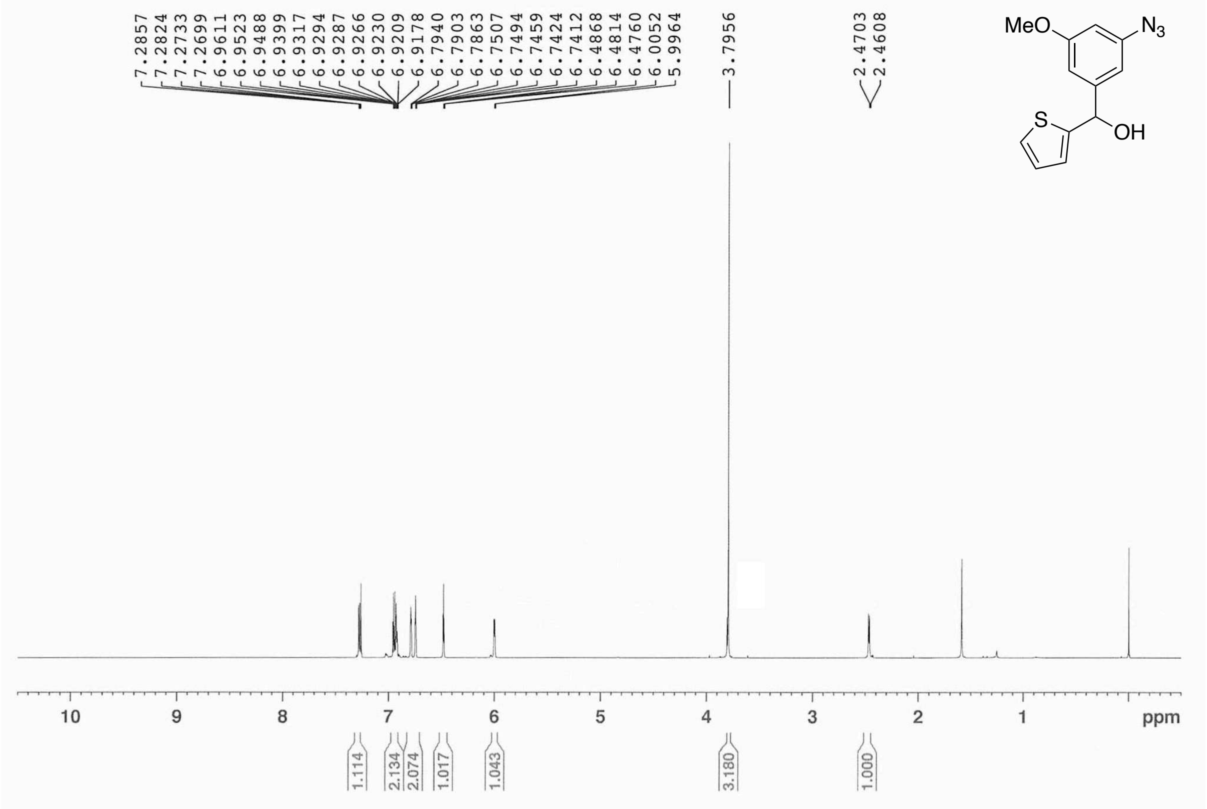

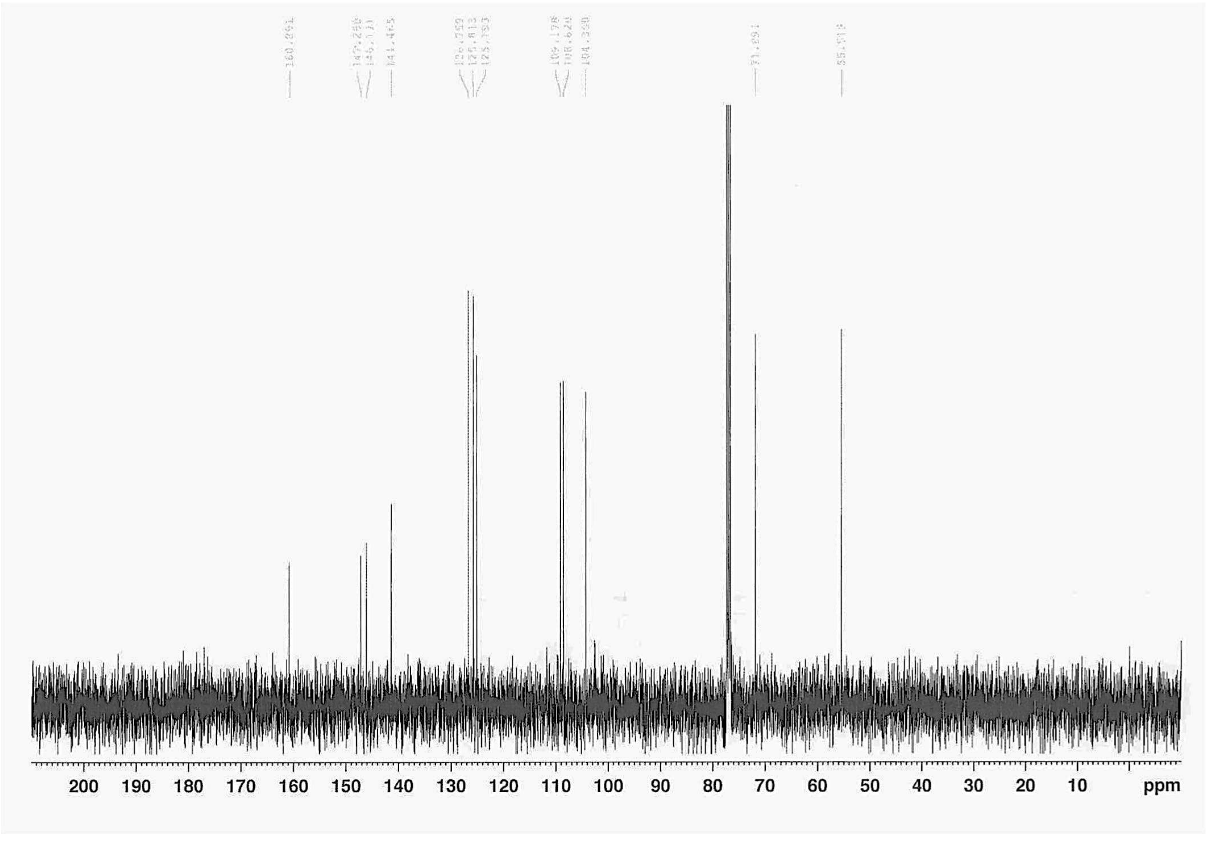

^{1}H NMR (400 MHz) and ^{13}C NMR (101 MHz) spectra of methyl 1-(3-(hydroxy(thiophen-2-yl)methyl)-5-methoxyphenyl)-1*H*-1,2,3-triazole-4-carboxylate (**21a**) ($CDCl_3$)

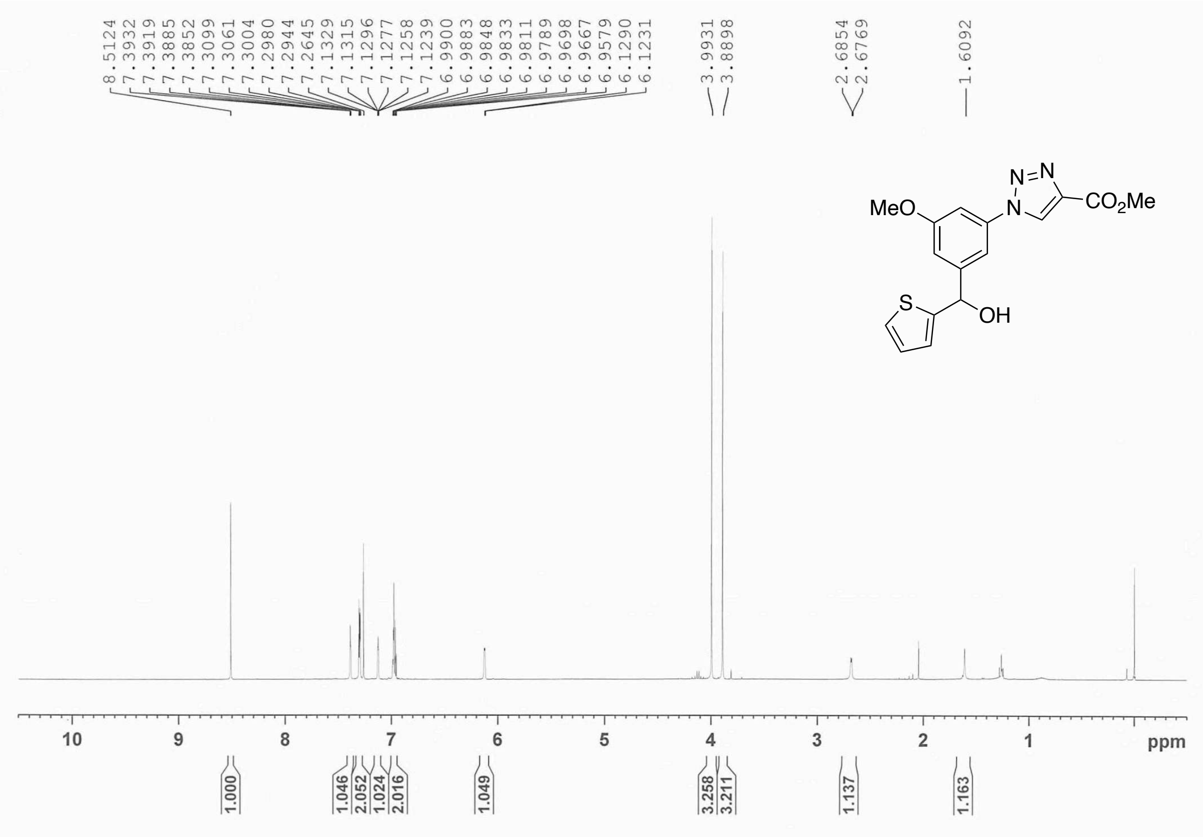

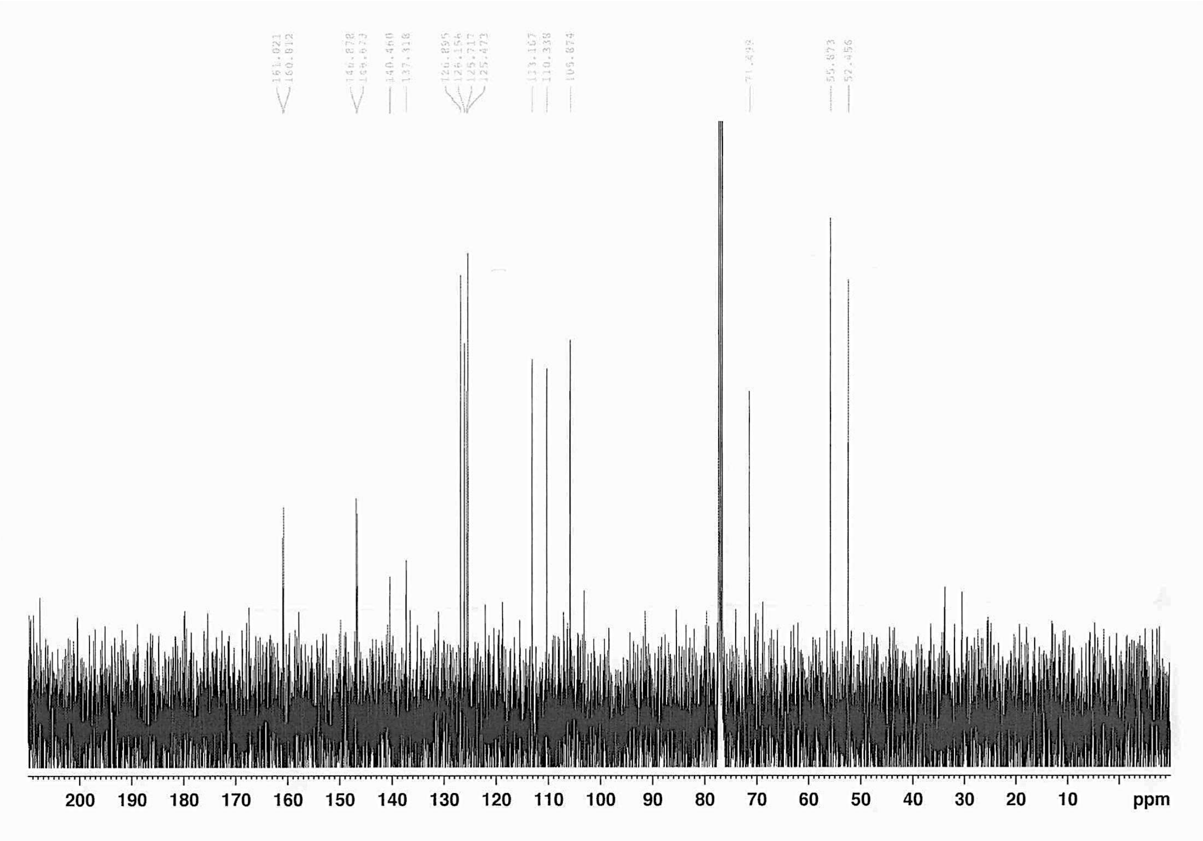

^{1}H NMR (400 MHz) and ^{13}C NMR (101 MHz) spectra of (3-methyl-4-(4-(*p*-tolyl)-1*H*-1,2,3-triazol-1-yl)phenyl)(phenyl)methanol (**21b**) ($CDCl_3$)

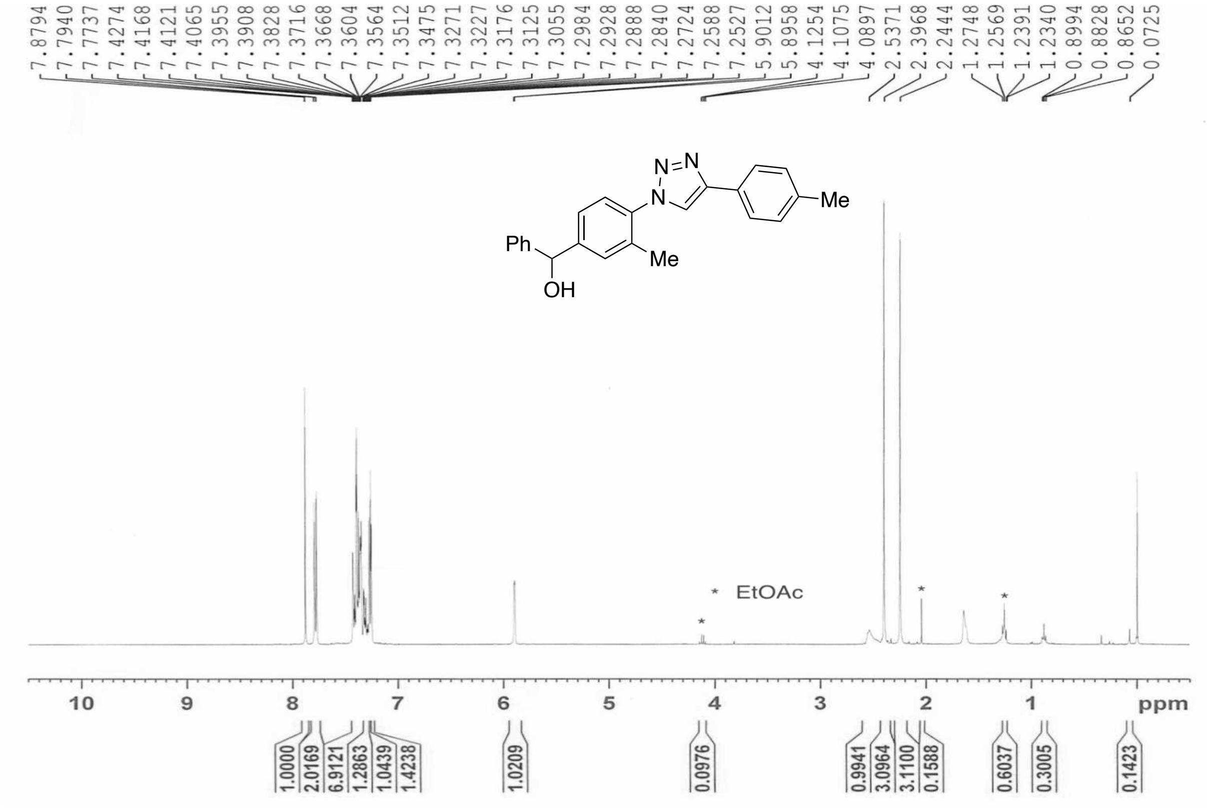

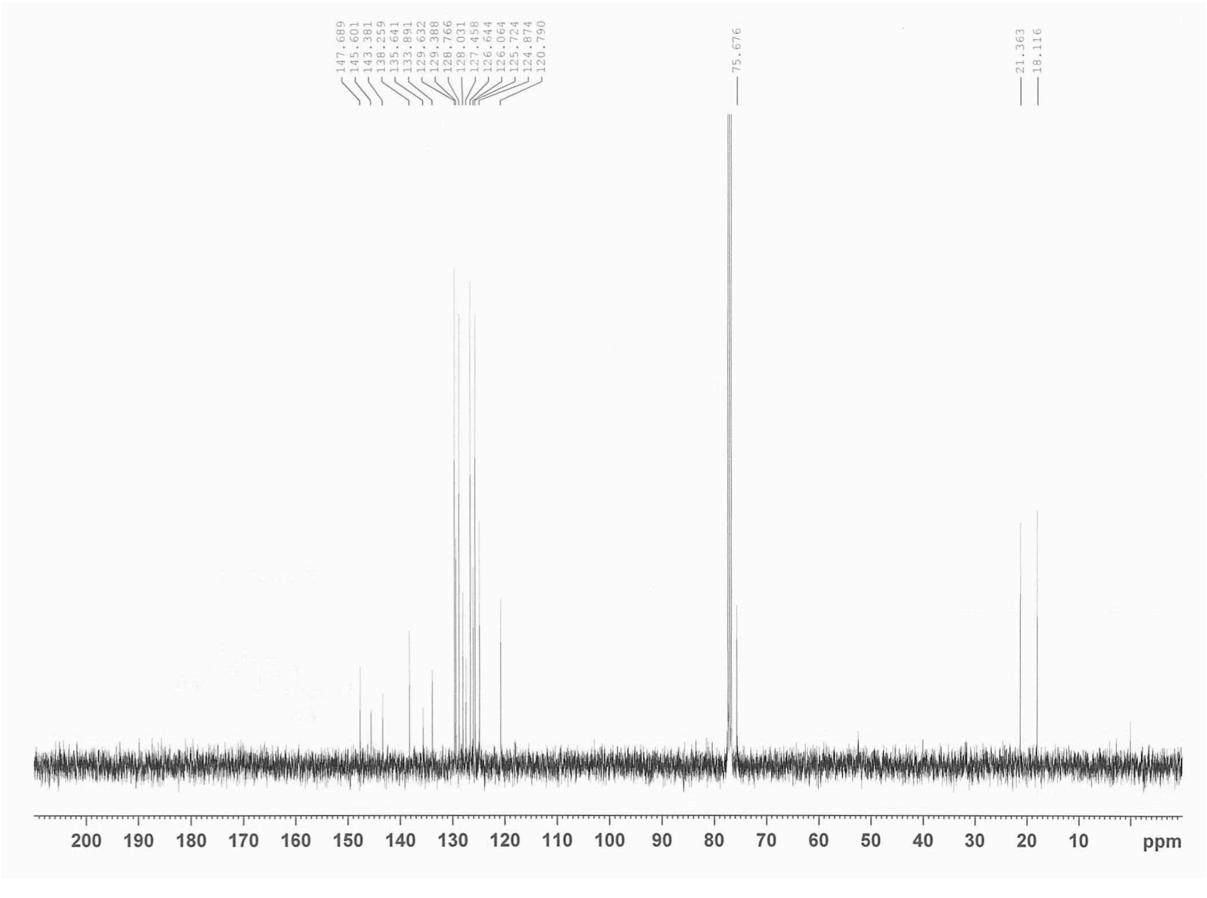

^{1}H NMR (400 MHz) and ^{13}C NMR (101 MHz) spectra of 2-(4-azidophenyl)propan-2-ol (**S3**) ($CDCl_3$)

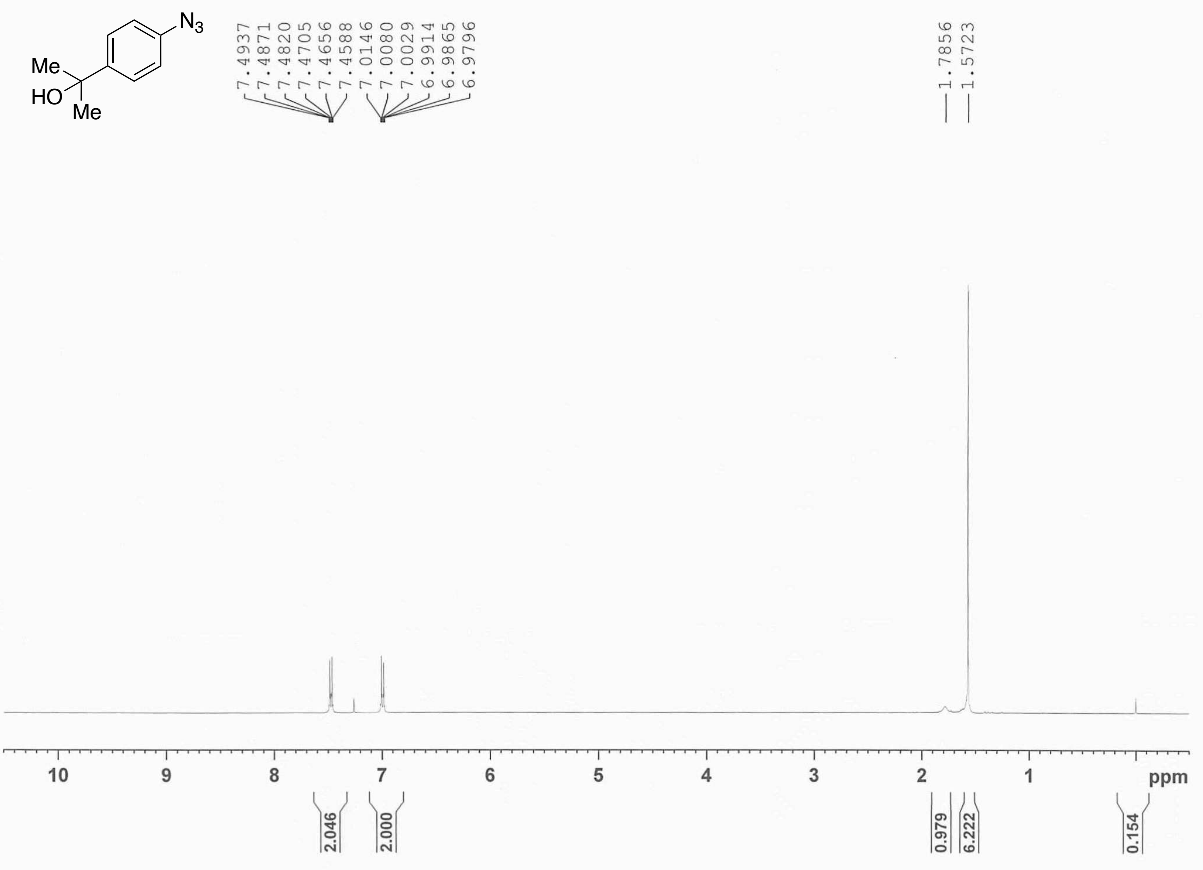

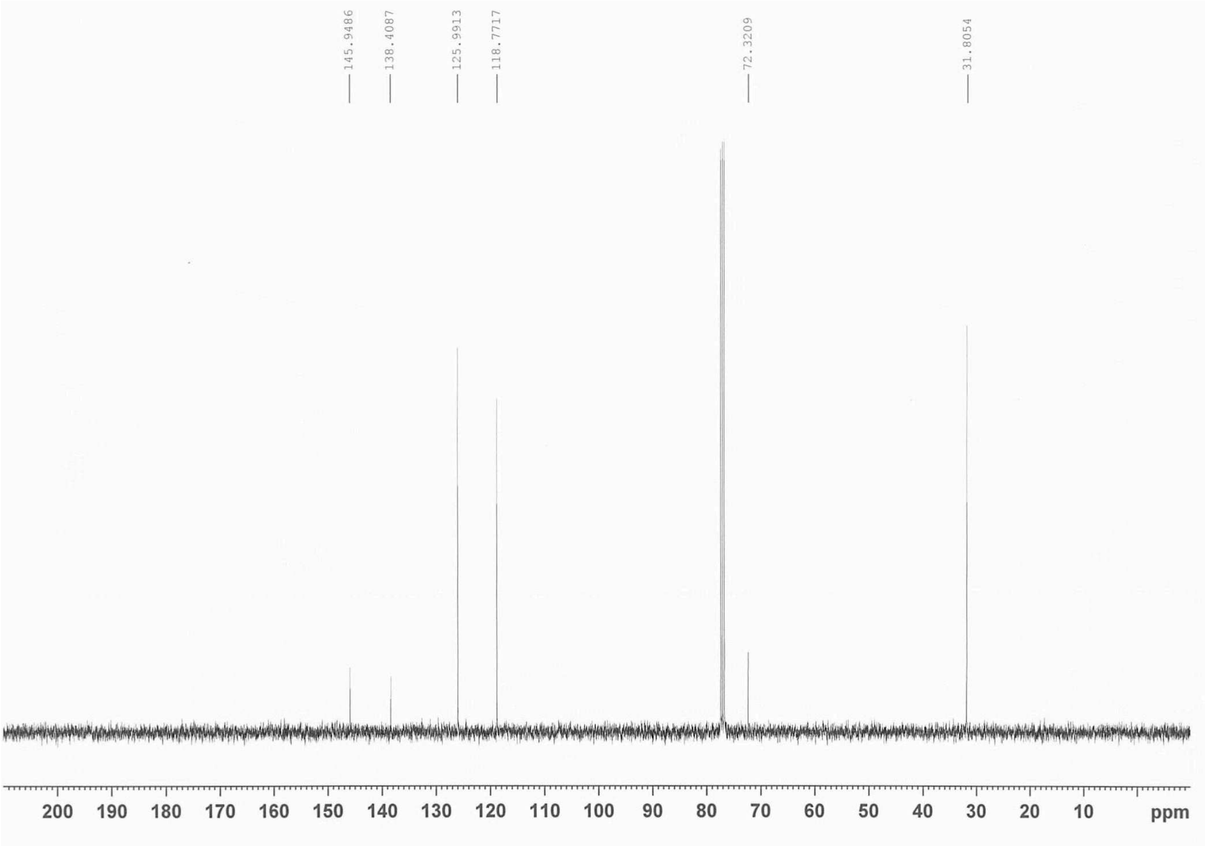

^{1}H NMR (400 MHz) and ^{13}C NMR (101 MHz) spectra of (8*R*,9*S*,13*S*,14*S*,17*S*)-17-(1-(4-(2-hydroxypropan-2-yl)phenyl)-1*H*-1,2,3-triazol-4-yl)-13-methyl-7,8,9,11,12,13,14,15,16,17-decahydro-6*H*-cyclopenta[*a*]phenanthrene-3,17-diol (**21c**) ($CDCl_3$)

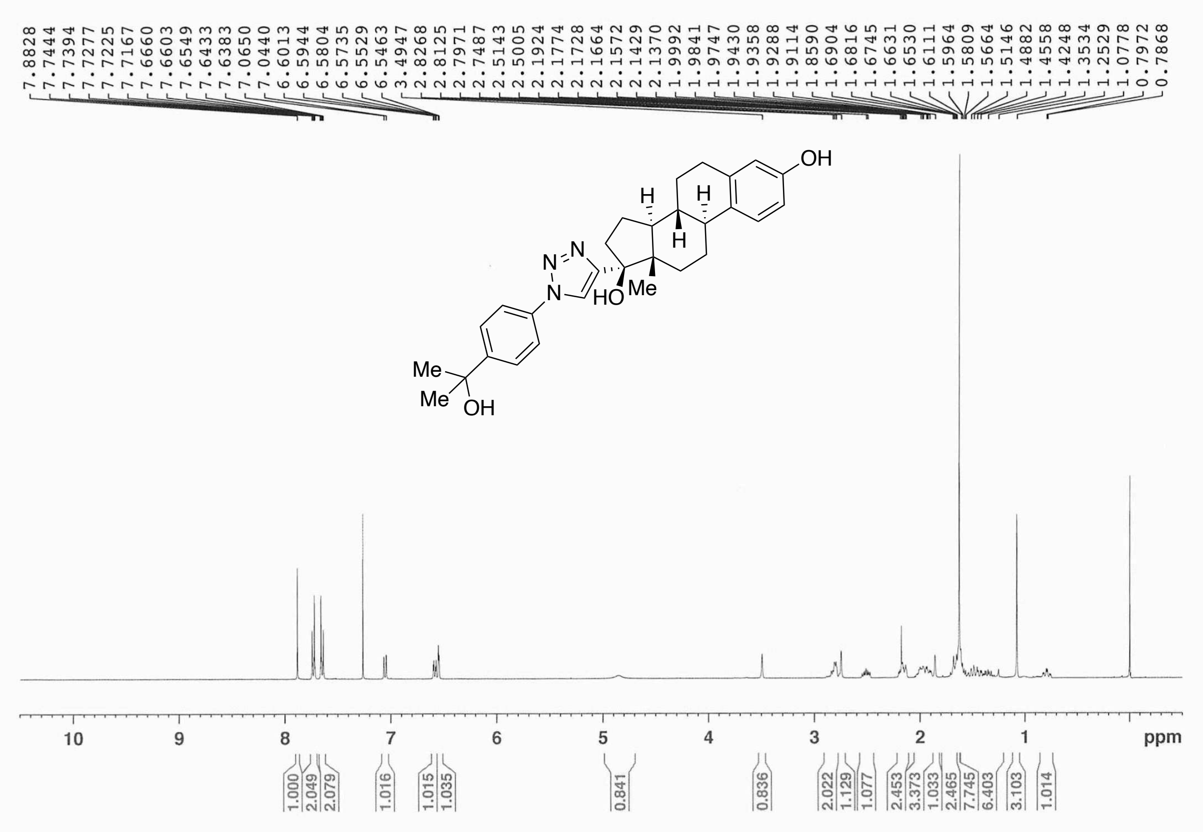

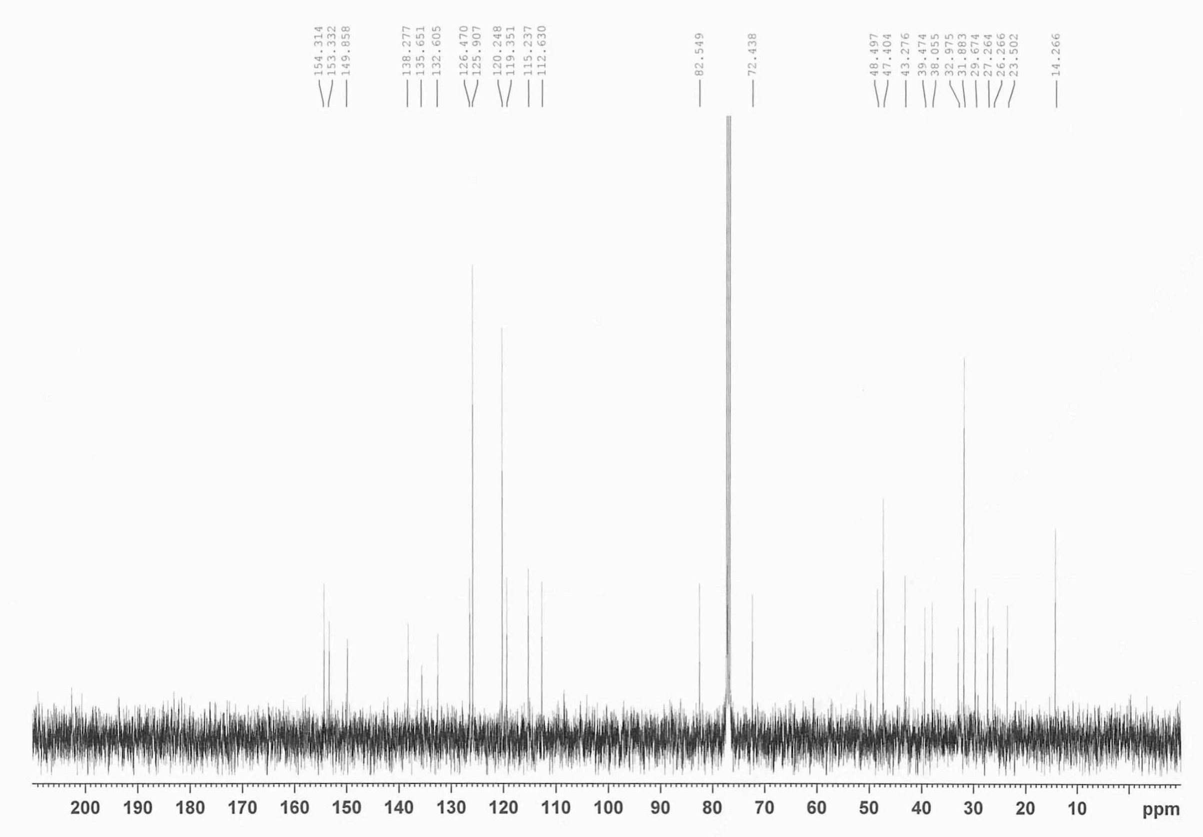

Supplement: Supplementary file 1 [file DataSheet1.PDF]
